# A novel estimator of between-study variance in random-effects models

**DOI:** 10.1186/s12864-020-6500-9

**Published:** 2020-02-11

**Authors:** Nan Wang, Jun Zhang, Li Xu, Jing Qi, Beibei Liu, Yiyang Tang, Yinan Jiang, Liang Cheng, Qinghua Jiang, Xunbo Yin, Shuilin Jin

**Affiliations:** 1 School of Mathematics, Harbin Institute of Technology, Harbin, Heilongjiang, China; 2 Rehabilitation department, Heilongjiang Province Land Reclamation Headquarters General Hospital, Harbin, Heilongjiang, China; 3 College of Computer Science and Technology, Harbin Engineering University, Harbin, Heilongjiang, China; 4 School of Mathematics, Heilongjiang University, Harbin, Heilongjiang, China; 5 Heilongjiang Province Hospital of Chinese Medicine, Harbin, Heilongjiang, China; 6 College of Bioinformatics Science and Technology, Harbin Medical University, Harbin, Heilongjiang, China; 7 School of Life Science and Technology, Harbin Institute of Technology, Harbin, Heilongjiang, China

**Keywords:** Differentially expressed genes, Between-study variance, Random-effects model, Meta-analysis

## Abstract

**Background:**

With the rapid development of high-throughput sequencing technologies, many datasets on the same biological subject are generated. A meta-analysis is an approach that combines results from different studies on the same topic. The random-effects model in a meta-analysis enables the modeling of differences between studies by incorporating the between-study variance.

**Results:**

This paper proposes a moments estimator of the between-study variance that represents the across-study variation. A new random-effects method (DSLD2), which involves two-step estimation starting with the DSL estimate and the $D_{g}^{2}$ in the second step, is presented. The DSLD2 method is compared with 6 other meta-analysis methods based on effect sizes across 8 aspects under three hypothesis settings. The results show that DSLD2 is a suitable method for identifying differentially expressed genes under the first hypothesis. The DSLD2 method is also applied to Alzheimer’s microarray datasets. The differentially expressed genes detected by the DSLD2 method are significantly enriched in neurological diseases.

**Conclusions:**

The results from both simulationes and an application show that DSLD2 is a suitable method for detecting differentially expressed genes under the first hypothesis.

## Background

With the advances of high-throughput experimental technology, a multitude of datasets have been produced and have resulted in several public databases, such as the European Bioinformatics Institute (EBI) and Gene Expression Omnibus database (GEO) [1]. A major challenge is how to re-exploit, re-extract and combine the information from a large number of datasets [2]. A meta-analysis, combining data or results from independent studies on the same topic, is widely applied and the major contribution is discovering disease pathogenesis [3, 4]. The statistical power could be raised through meta-analysis by combining information from individual studies that have small sample sizes [5, 6]. Although many significantly differential gene expression lists are presented, individual conclusions tend to be discordant because of various study designs, individual treatment protocols, limited sample sizes and different genders among the study participants [7]. Meta-analysis is an important method for providing reliable and consistent differentially expressed gene lists by integrating information on the same disease [8]. As meta-analysis methods use available datasets, they are relatively inexpensive [9]. But not all datasets are usually available due to publication bias and outcome reporting bias [10].

Meta-analysis methods based on effect sizes, which contribute to the early diagnosis and treatment of diseases, can be broadly divided into two classes: the fixed-effects model (FEM) and the random-effects model (REM) [11]. The fixed-effects model assumes that all studies in a meta-analysis have the same true effect size [12]. The random-effects model assumes that different studies in a meta-analysis have the different true effect sizes [12]. Meta-analyses were first introduced to microarray data by Rhode et al. (2002) [13] and Choi et al. (2003) [14]. Many meta-analysis methods, including DerSimonian and Laird estimate (DSL) [15], restricted maximum likelihood estimate (RML) and Sidik and Jonkman estimate (SJ) [16], were later applied to microarray studies. Two-step estimate starting with the DSL estimate (DL2) is an iterative estimator. The two-step method DL2 and the iterative Paule and Mandel method are close [17]. The random-effects methods in meta-analysis make possible the modeling of differences and the differences between studies often caused by the study design, sample sizes, sex/gender differences in participants and so on. The between-study variance *τ*^2^ is incorporated by random-effects methods in meta-analyses to estimate the across-study variation [18]. The fixed-effects model in meta-analyses excludes the between-study variance *τ*^2^ from the random-effects model [19].

This paper develops an estimator of the between-study variance $D_{g}^{2}$ which originates from the general moments estimator described by DerSimonian and Kacker (2007). Therefore, a new random-effects method (DSLD2) based on $D_{g}^{2}$ is presented. In subsequent sections, three hypothesis testing frameworks were thoroughly reviewed. We observed the biases and root mean square errors (RMSE) of between study variance $D_{g}^{2}$. The random-effects method based on $D_{g}^{2}$ and other meta-analysis models were applied to simulation datasets of gene expression levels. Then, we compared the DSLD2 method with other meta-analysis methods, including the DSL method, the DSLR2 method, the fixed-effects model, the PM method, the RML method and the SJ method across the following metrics: the false discovery rates (FDRs), accuracy, precision, false positive rate (FPR), sensitivity, precision-recall curve and the receiver operating characteristic curve (ROC). DSLD2 performed well among the meta-analysis methods based on effect sizes under the first hypothesis. We also applied DSLD2 to Alzheimer’s disease. The pathways of differentially expressed genes detected by the DSLD2 method indicate that Alzheimer’s disease is related to the nervous system, which is obvious. The results from both the simulation and the application suggest that DSLD2 is appropriate for identifying differentially expressed genes. In addition, we prove the reasonableness of the between-study variance $D_{g}^{2}$ in Additional file 1.

## Methods

### Underlying hypothesis settings

Statistical hypothesis tests are primarily used in meta-analyses to identify differentially expressed genes, and three common hypothesis testing frameworks are often applied [20]. In the first hypothesis test, targeted biomarkers are differentially expressed genes with non-zero effect sizes in all studies. The null and alternative hypotheses are as follows:

$$H_{0}\!:\!\underset{i=1}{\overset{k}{\cap}}\{\theta_{ig}=0\} \ vs \ H_{A}\!:\!\underset{i=1}{\overset{k}{\cap}}\{\theta_{ig}\not=0\} \text{(The\ first \ hypothesis)} $$

where *θ*_*ig*_ denotes the underlying true effect size for gene *g* in study *i* (*i*=1,2,⋯,*k*), *k* is the number of studies in a meta-analysis. The second hypothesis test aims to determine a differentially expressed gene with non-zero effect sizes in one or more studies. The null and alternative hypotheses are as follows:

$${\begin{aligned} H_{0}:\underset{i=1}{\overset{k}{\cap}}\{\theta_{ig}=0\} \ vs \ H_{A}:\underset{i=1}{\overset{k}{\cup}}\{\theta_{ig}\not=0\} \text{(The\ second \ hypothesis)} \end{aligned}} $$

The third hypothesis test aims to determine a differential gene expression if it has non-zero effect sizes in the majority of studies (half or more). The null and alternative hypotheses are as follows:

$${\begin{aligned} H_{0}\!:\!\sum_{i=1}^{k}I\{\theta_{ig}\not=0\}<r\ vs\ H_{A}: \sum_{i=1}^{k}I\{\theta_{ig}\not=0\}\ge r \text{(The\ third\ hypothesis)} \end{aligned}} $$

where the indicator function is denoted by *I*(.), which takes a value of 0 if *θ*_*ig*_=0 and a value of 1 if *θ*_*ig*_≠0. *r* is the number of studies that we identify a differentially expressed gene in at least *r* studies. *r* is usually set as greater than 0.5*k*. For instance, we can define a differentially expressed gene if it is significant in at least 4 (*r*=4) of 8 studies.

### Meta-analysis methods based on effect sizes

#### Fixed-effects model

The fixed-effects model (FEM) assumes that all studies included in the meta-analysis have the same true effect size and that the difference in the observed effect between combined studies is caused by random error [21]. The observed effect sizes of each study are combined with a simple linear model.

#### Random-effects model

Let *μ*_*g*_ be the overall mean for gene *g*, which is a typical parameter of interest. *y*_*ig*_ denotes the observed effect size for gene *g* in study *i* (*i*=1,2,⋯,*k*). The random-effects model is given by

$$y_{ig}=\mu_{g} + \xi_{ig} + \varepsilon_{ig},\xi_{ig}\sim N\left(0,\tau_{g}^{2}\right),\varepsilon_{ig}\sim N\left(0,\sigma_{ig}^{2}\right) $$

where *ξ*_*ig*_ is the random effect for gene *g* in study *i* and obeys a normal distribution with mean 0 and variance $\tau _{g}^{2}, \sigma _{ig}^{2}$ is the within-study variance representing the sampling error for gene *g* in study *i*, and $\tau _{g}^{2}$ denotes the between-study variance which is the variability between studies. If $\tau _{g}^{2}=0$, then the random-effects model reduces to a fixed-effects model. If $\hat {\sigma }_{ig}^{2} (i=1,2,\cdots,k)$ and $\hat {\tau }_{g}^{2}$ are the estimates of $\sigma _{ig}^{2} (i=1,2,\cdots,k)$ and $\tau _{g}^{2}$, the overall mean *μ*_*g*_ can be estimated by

1 $$ M_{g}^{*}=\frac{\sum_{i=1}^{k}\omega_{ig}^{*}y_{ig}}{\sum_{i=1}^{k}\omega_{ig}^{*}}, \omega_{ig}^{*}=\left(\hat{\tau}_{g}^{2} + \hat{\sigma}_{ig}^{2}\right)^{-1}.  $$

#### DerSimonian and Laird estimate

The between-study variance $\tau _{g}^{2}$ can be estimated by the DerSimonian–Laird (DSL) method

2 $$  \widehat{\tau}_{g}^{2}(DSL)=max\left(0, \frac{Q_{g}-(k-1)}{\sum_{i=1}^{k}\omega_{ig}-\sum_{i=1}^{k}\omega_{ig}^{2}/\sum_{i=1}^{k}\omega_{ig}}\right)  $$

where $Q_{g}=\sum _{i=1}^{k}\omega _{ig}(y_{ig}-M)^{2}, \omega _{ig}=\hat {\sigma }_{ig}^{-2}, M=\sum _{i=1}^{k}\omega _{ig}y_{i}/\sum _{i=1}^{k}\omega _{ig}$ [22]. The estimator is not unbiased, but it is the simplest [23]. The DSL estimator is the most widely used method [24].

#### Two-step estimation starting with the DSL estimate and the $R_{g}^{2}$ in the second step (DSLR2)

DSLR2 is a random-effects model based on the between-study variability $R_{g}^{2}$ [17, 25], which yields

$$ R_{g}^{2}=1-\frac{\sum_{i=1}^{k}\omega_{ig}^{*}(y_{ig}-M_{g}^{*})^{2}}{\sum_{i=1}^{k}\omega_{ig}(y_{ig}-M)^{2}} $$

where $\omega _{ig}^{*}=\left (\hat {\sigma }_{ig}^{2} + \hat {\tau }_{g}^{2}(DSL)\right)^{-1}, M_{g}^{*}=\sum _{i=1}^{k}\omega _{ig}^{*}y_{i}/ \sum _{i=1}^{k}\omega _{ig}^{*}.$

#### Paule and Mandel estimate (PM)

$\hat {\tau }_{g}^{2}(PM)$ is the unique solution of $\sum _{i=1}^{k}\omega _{ig}^{*} \left (y_{ig}-M_{g}^{*}\left (\hat {\tau }_{g}^{2}(PM)\right)\right)-(k-1)=0$, where $\omega _{ig}^{*}=(\hat {\sigma }_{ig}^{2} + \hat {\tau }_{g}^{2}(PM))^{-1}$ [26]. Negative $\hat {\tau }_{g}^{2}(PM)$ estimates truncate to 0. $\hat {\tau }_{g}^{2}(PM)$ is estimated and we can substitute it in $\omega _{ig}^{*}=\left (\hat {\sigma }_{ig}^{2} + \hat {\tau }_{g}^{2}(PM)\right)^{-1}$ to obtain $M_{g}^{*}\left (\hat {\tau }_{g}^{2}(PM)\right) = \sum _{i=1}^{k}\omega _{ig}^{*}y_{i}/\sum _{i=1}^{k}\omega _{ig}^{*}$ [26].

#### Restricted maximum likelihood estimate

The method of restricted maximum likelihood estimate (RML) can be used to calculate the estimators of overall mean value *μ*_*g*_ and between-studies variance $\tau _{g}^{2}$ of a random-effects meta-analysis model [27]. The log-likelihood function based on the linear mixed effects model is

$${\begin{aligned} l_{g}\left(\mu_{g},\tau_{g}^{2}\right)&=-\frac{1}{2}\sum_{i=1}^{k}ln\left(\sigma_{ig}^{2}+\tau_{g}^{2}\right)\\ &\quad -\frac{1}{2}\sum_{i=1}^{k}\frac{(y_{ig}-\mu_{g})^{2}}{\sigma_{ig}^{2}+\tau_{g}^{2}}-\frac{1}{2}ln\left(\sum_{i=1}^{k}\left(\sigma_{ig}^{2}+\tau_{g}^{2}\right)^{-1}\right)\!. \end{aligned}} $$

The log-likelihood can be maximized using the Fisher scoring algorithm to obtain the estimates of *μ*_*g*_ and $\tau _{g}^{2}$. Negative $\tau _{g}^{2}$ estimates are truncated to 0 [27].

#### Sidik and Jonkman estimate

The following two-step estimator of between-study variance $\tau _{g}^{2}$ was proposed by Sidik and Jonkman [28, 29]

$$ \hat{\tau}_{g}^{2}(SJ)=\frac{1}{k-1}\sum_{i=1}^{k}\frac{1}{1+\hat{\sigma}_{ig}^{2}/\hat{\tau}_{0g}^{2}}\left(y_{ig}-M_{g}^{*}(SJ)\right)^{2} $$

where $M_{g}^{*}(SJ)=\sum _{i=1}^{k}\omega _{ig}^{*}y_{ig}/\sum _{i=1}^{k}\omega _{ig}^{*}, \omega _{ig}^{*}=\frac {1}{1+\hat {\sigma }_{ig}^{2}/\hat {\tau }_{0g}^{2}}, \hat {\tau }_{0g}^{2}=max\left \{0.01, \frac {1}{k-1}\sum _{i=1}^{k}(y_{ig}-y_{Ag})^{2}-\frac {1}{k}\sum _{i=1}^{k}\hat {\sigma }_{ig}^{2}\right \}, y_{Ag}=\frac {1}{k}\sum _{i=1}^{k}y_{ig}$.

#### Two-step estimation starting with the DSL estimate and the $D_{g}^{2}$ in the second step (DSLD2)

The main component of the random-effects meta-analysis model is the between-study variability. We develop a between-study variability estimator $D_{g}^{2}$, which estimates the amount of conditional variance in *y*_*ig*_, which yields

3 $$ D_{g}^{2}=\frac{Q_{g}-S_{MM,g}}{\sum_{i=1}^{k}\omega_{ig}-\sum_{i=1}^{k}\omega_{ig}^{2}/\sum_{i=1}^{k}\omega_{ig}}  $$

where $Q_{g}=\sum _{i=1}^{k}\frac {(y_{ig}-M_{g})^{2}}{\hat {\sigma }_{ig}^{2}}, S_{MM,g}=\sum _{i=1}^{k}\frac {(y_{ig}-M_{g}^{*})^{2}}{\hat {\sigma }_{ig}^{2}+\hat {\tau }_{g}^{2}}, M_{g}=\frac {\sum _{i=1}^{k}\omega _{ig}y_{ig}}{\sum _{i=1}^{k}\omega _{ig}}, \omega _{ig}=\hat {\sigma }_{ig}^{-2}$ and $M_{g}^{*}=\frac {\sum _{i=1}^{k}y_{ig}/\left (\hat {\sigma }_{ig}^{2} + \hat {\tau }_{g}^{2}\right)}{\sum _{i=1}^{k}1/\left (\hat {\sigma }_{ig}^{2} + \hat {\tau }_{g}^{2}\right)}$.

Such an estimator of the between-study variance is always greater than 0 and indicates how strong the random effects are. The algorithm of the DSLD2 method is as follows:

- Calculate *Q*_*g*_ and $\hat {\tau }_{g}^{2}$ in Eq. (2),
- Calculate $M_{g}^{*}$ in Eq. (1),
- Calculate $D_{g}^{2}$ in Eq. (3) and
- Replace $\hat {\tau }_{g}^{2}$ with $D_{g}^{2}$ in Eq. (1).

The weights, overall mean estimator, variance of the overall mean estimator, bounds of the confidence interval and *z*-statistics based on the between-study variance $D_{g}^{2}$ can be obtained by

$$ \omega_{ig}\left(D_{g}^{2}\right)=\frac{1}{\hat{\sigma}_{ig}^{2} + D_{ig}^{2}},i=1,2,\cdots,k $$

$$ M_{g}\left(D_{g}^{2}\right)=\frac{\sum_{i=1}^{k}\omega_{ig}\left(D_{g}^{2}\right)y_{ig}}{\sum_{i=1}^{k}\omega_{ig}\left(D_{g}^{2}\right)} $$

$$ Var\left(D_{g}^{2}\right)=\frac{1}{\sum_{i=1}^{k}\omega_{ig}\left(D_{g}^{2}\right)} $$

$$ UL\left(D_{g}^{2}\right)=M_{g}\left(D_{g}^{2}\right) + 1.96*\sqrt{Var\left(D_{g}^{2}\right)} $$

$$ LL\left(D_{g}^{2}\right)=M_{g}\left(D_{g}^{2}\right) - 1.96*\sqrt{Var\left(D_{g}^{2}\right)} $$

and

$$ z_{g}\left(D_{g}^{2}\right)=\frac{M_{g}\left(D_{g}^{2}\right)}{\sqrt{Var\left(D_{g}^{2}\right)}}. $$

## Simulation and application

### Meta-analysis methods used in simulation datasets

Two class simulation datasets were generated to observe the performance of DSLD2 method. The methods used in simulation datasets of gene expression levels were the fixed-effects model (FEM), the random-effects model based on DerSimonian and Laird estimate for $\tau _{g}^{2}$ (DSL), the random-effects model based on the between-study variance estimotor $R_{g}^{2}$ (DSLR2), the random-effects model based on Paule and Mandel estimate for $\tau _{g}^{2}$ (PM), the random-effects model based on the restricted maximum likelihood estimate for $\tau _{g}^{2}$ (RML), the random-effects model based on Sidik and Jonkman estimate for $\tau _{g}^{2}$ (SJ) and the random-effects model based on the between-study variance estimote $D_{g}^{2}$ (DSLD2). We compared the performances of DSLD2 method and other 6 meta-analysis methods based on effect-sizes in histograms, precision, accuracy, the false discovery rates (FDRs), false positive rate (FPR), Matthews correlation coefficient (MCC), sensitivity, receiver operating characteristic curves (ROC) and precision-recall curves under three hypotheses using simulation datasets of gene expression levels. We reported the bias and root mean square error (RMSE) of the between-study variance estimators $D_{g}^{2}$ through Monte Carlo simulation datasets.

### Simulation setting of gene expression levels

A common method was used to produce simulation data for comparing the ability of detecting DE genes among 16 meta-analysis methods under the three hypothesis settings [30]. Five studies were simulated (*k*=1,2,⋯,5). Each study contained 2000 genes and 2*N* samples (2*N*=10,20,60,100,140,180,220). In each study, the first *N* samples were controls, and the last *N* samples were cases. Each sample in each study contained 40 gene clusters (*C*_*g*_=1,2,⋯,40), and each cluster included 20 genes $(\sum I(C_{g}=c)=20,c=1,2,\cdots,40).$ The remaining 1200 genes had 0 gene clusters $(\sum I(C_{g}=0)=1200)$. The first 1000 genes in each study were divided into 5 groups (*k*_*g*_=1,2,3,4,5). The first 200 genes were put into the first group (*k*_*g*_=1). The 201th gene to the 400th gene were put into the second group (*k*_*g*_=2). The 401th gene to the 600th gene were put into the third group (*k*_*g*_=3). The 601th gene to the 800th gene were put into the fourth group (*k*_*g*_=4). The 801th gene to the 1000th gene were put into the fifth group (*k*_*g*_=5). The 1001th gene to the 2000th gene were put into the zeroth group (*k*_*g*_=0). The simulation algorithm is summarized as follows:

- We sampled $\sum _{ck}^{'}\sim W^{-1}(\psi,60)$ for genes in cluster *c* (1≤*c*≤40) and study *k* (1≤*k*≤5), where *ψ*=0.5*I*_20×20_+0.5*J*_20×20_,*I*_20×20_ was the identity matrix, *J*_20×20_ was the matrix in which all elements equal 1, and *W*^−1^ denoted the inverse Wishart distribution. We then standardized $\sum _{ck}^{'}$ into $\sum _{ck}$ with all diagonal elements equaling 1.
- We sampled the expression levels of genes in clusters *c* and *n* as $\left (X_{g_{c1}nk}^{'}, \cdots, X_{g_{c20}nk}^{'}\right)^{T} \sim MVN\left (0, \sum _{ck}\right)$, where 1≤*n*≤2*N*,1≤*c*≤40 and 1≤*k*≤5. The gene expression levels are $g \sim N\left (0,\sigma _{k}^{2}\right)$ for the gene in cluster 0, where $\sigma _{k}^{2} \sim U(0.8,1.2), 1 \leq n \leq 2N$ and 1≤*k*≤5.
- We randomly sampled *δ*_*gk*_∈{0,1} such that $\sum _{k=1}^{5} \delta _{gk}=k_{g} (k_{g}=1,2,\cdots 5)$. When *δ*_*gk*_=1, the gene *g* in study *k* was DE, and we sampled *μ*_*gk*_∼*U*(0.5,3). The expression level of the control samples remained unchanged, and the case samples were $Y_{gnk}=X_{g(n+N)k}^{'} + \mu _{gk} \cdot \delta _{gk}$, where 1≤*g*≤2000,1≤*n*≤*N*, and 1≤*k*≤5.

Thus, the numbers for truly differentially expressed genes were 200, 1000 and 600 under the first hypothesis, the second hypothesis and the third hypothesis, respectively.

### Simulation setting using Monte Carlo method

Let $X_{ijg}^{ctrl}$ and $X_{ijg}^{case}$ be the observations of *g*th iteration for *j*th samples in the *i*th study from a control and a case group. Assume that $X_{ijg}^{ctrl}$ was sampled $N\left (\mu _{i}^{ctrl}, \sigma _{i}^{2}\right)$ and $X_{ijg}^{case}$ was sampled $N\left (\mu _{i}^{case}, \sigma _{i}^{2}\right)$. Let $n_{i}^{ctrl}$ and $n_{i}^{case}$ be the sample sizes in *i*th study. To simplify things, it was set that $n_{i}=n_{i}^{ctrl}=n_{i}^{case}, \sigma _{i}^{2}=10$ and $\mu _{i}^{ctrl}=0$. $\mu _{i}^{case}$ was sampled from *N*(0,*τ*^2^) The following factors were set in the simulations: $k=(5,10,20,40,80), \tau ^{2}=(0.0, 1.0), \overline {n}_{i}=40$ and *g*=1,2,⋯,1000. The values of *n*_*i*_ were sampled from *N*(40,(40/3)^2^). The standardized mean difference (SMD) and the mean difference (MD) were chosen as the effect size measures.

## Results

### Simulation results

The numbers of differentially expressed genes with *p*<0.05 (DE_1_) or FDR<0.05(DE_2_) identified by various meta-analysis models are presented in Table 1 [31]. More differentially expressed genes were identified by the fixed-effects model. The DSLD2 method detected fewer DE genes than the FEM and SJ methods. All methods had normal FDR_1_ levels and FDR_2_ levels except the FEM method. The FDR_2_ of FEM is 0.3808 and greater than other metaanalysis methods. The FDR_1_ value of DSLD2 method was 0.0165, which was greater than that of the DSLR2 methods. However, the FDR_1_ value of the DSLD2 method was smaller than that of the other 5 meta-analysis methods. The FDR_2_ value of the DSLD2 method was 0.0236, which was the smallest among 7 meta-analysis methods based on effect sizes.

**Table 1:** The number of DE genes and FDRs from each method in the simulation data

Method	Simulation: *K*=5,*N*=100,*G*=2000			
	DE_1_(*p*<0.05)	DE_2_(*FDR*<0.05)	FDR_1_	FDR_2_
DSLD2	642	422	0.0165	0.0236
DSLR2	609	425	0.0164	0.0352
DSL	625	426	0.0258	0.0328
PM	621	422	0.0260	0.0331
FEM	1033	969	0.0361	0.3808
RML	620	423	0.0260	0.0330
SJ	696	483	0.0434	0.0641

Note: *K* represents the number of studies on the same or related topic; *N* denotes the number of the samples in every study; *G* represents the number of genes in every sample; DE_1_ represents the number of DE genes with *p*<0.05; DE_2_ represents the number of DE genes for which FDR<0.05. FDR_1_ and FDR_2_ are obtained from Additional file 2; DSLD2 represents the random-effects methods based on $D_{g}^{2}$ proposed in this paper; DSLR2 represents the random-effects method based on *R*^2^; DSL denotes the standard random-effects model; FEM is the fixed-effects model

**Fig. 1:**
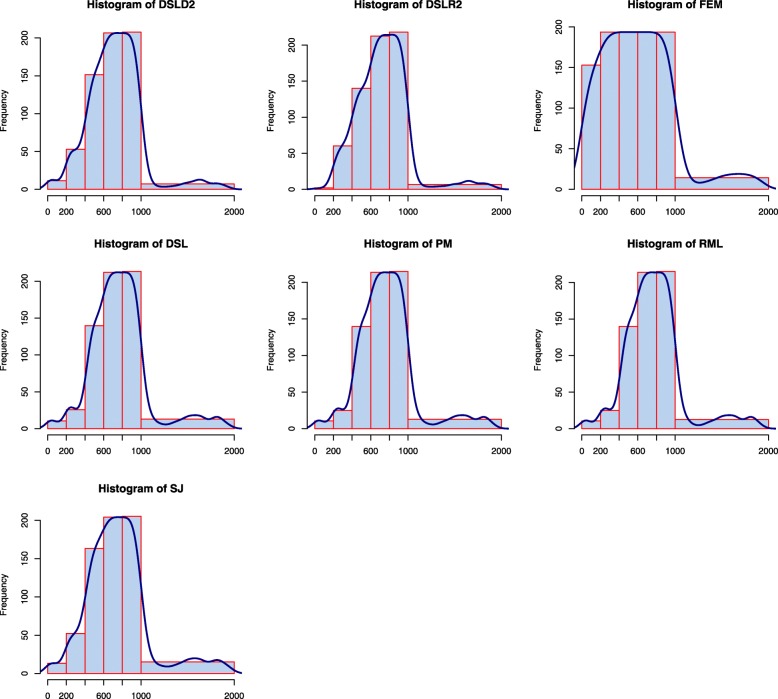
The histograms of DE genes detected by the 7 meta-analysis methods. The DSLD2 method is proposed in this paper. Genes 1 to 200 are differentially expressed in only one study; genes 201 to 400 are differentially expressed in two studies; genes 401 to 600 are differentially expressed in three studies; genes 601 to 800 are differentially expressed in four studies; genes 801 to 1000 are differentially expressed in all studies; and genes 1001 to 2000 are not differentially expressed in any studies

Histograms were constructed to compare the differences in differentially expressed genes (*p*<0.05) among different groups detected by various meta-analysis methods (see Fig. 1). The numbers of studies that were differentially expressed for gene *g* in 1∼200,201∼400,401∼600,601∼800,801∼1000,1001∼2000 were 1, 2, 3, 4, 5 and 0, respectively. The DSLD2 method identified fewer DE genes in group 1, group 2 and group 0 (see Fig. 1). More differentially expressed genes were detected by the DSLD2 method in groups 3, 4 and 5 (see Fig. 1). The DE genes discovered by the DSLD2 method showed an increasing trend, and the differentially expressed genes in group 5 could be completely identified by the DSLD2 method (see Fig. 1). The numbers of DE genes identified by the DSLD2 method in every group were consistent with the data simulation method (see Fig. 1).

**Fig. 2:**
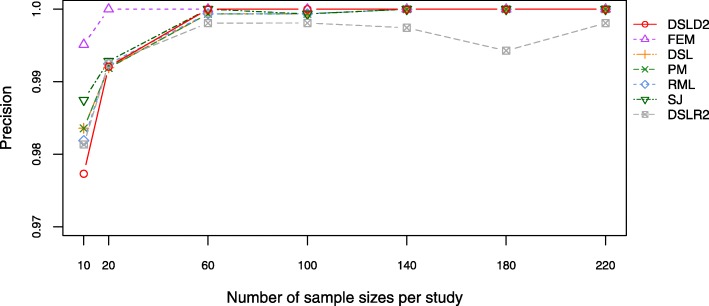
Plot of the precision under the first hypothesis. The DSLD2 method is developed in this paper. The precision values of DSLD2 method go up to 1.0 when numbers of sample sizes per study are larger than 60

Precision is an important descriptor of random errors. Line graphs and tables were constructed to compare the precision among 7 meta-analysis methods (see Fig. 2, Additional file 3: Figures S1-S2 and Additional file 4: Tables S1–S3). The precision of all the methods increased significantly from 10 to 60 samples and fluctuated slightly between 60 and 220 samples under the first hypothesis, the second hypothesis and the third hypothesis (see Fig. 2, Additional file 3: Figures S1–S2). The precision of the DSLD2 method was lower than other methods in 10 studies, however, the precision values of DSLD2 method went up to 1.0 when numbers of sample sizes per study were larger than 60 under the first hypothesis. Under the first hypothesis, the DSLR2 method had the lowest precision among the meta-analysis methods combining effect sizes. Under the second and third hypothesis, the FEM method had the highest precision values among 7 meta-analysis methods combining effect sizes (see Additional file 3: Figures S1, S2 and Additional file 4: Tables S2, S3).

**Fig. 3:**
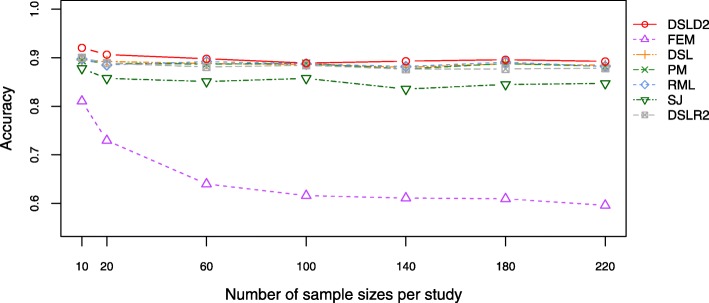
Plot of accuracy under the first hypothesis. The DSLD2 method is proposed in this paper. The accuracy of the DSLD2 method is the highest among that of the 7 meta-analysis methods

Accuracy is a critical descriptor of systematic errors. Among the meta-analysis methods based on effect sizes, DSLD2 had the highest accuracy among 7 meta-analysis methods based on effect sizes under the first hypothesis (see Fig. 3 and Additional file 4: Table S4). Under the first hypothesis, the accuracy of the DSLD2 method experienced a decrease from 10 to 100 samples and tended to be steady between 100 and 220 samples (Fig. 3). The accuracy of FEM method is the lowest among 7 meta-analysis methods based on effect sizes under the first hypothesis (see Fig. 3 and Additional file 4: Table S4). Under the second hypothesis, the accuracy of FEM method was highest among 7 meta-analysis methods (Additional file 3: Figure S3 and Additional file 4: Table S5). Under the third hypothesis, the SJ method had the highest accuracy values among 7 meta-analysis methods when the numbers of sample sizes per study were between 60 and 220 (Additional file 3: Figure S4 and Additional file 4: Table S6).

**Fig. 4:**
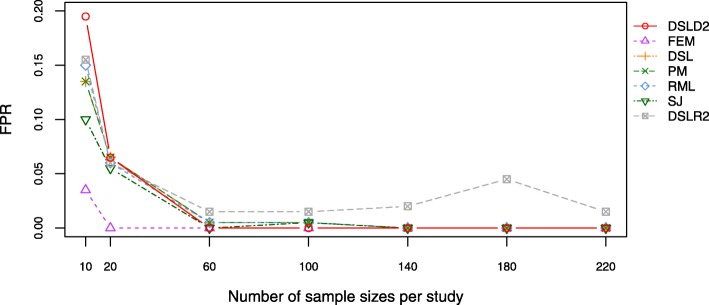
Plot of FPR under the first hypothesis. The DSLD2 method was introduced in this paper. The FPR values of DSLD2 method go down to 0.0 when numbers of sample sizes per study are larger than 60

**Fig. 5:**
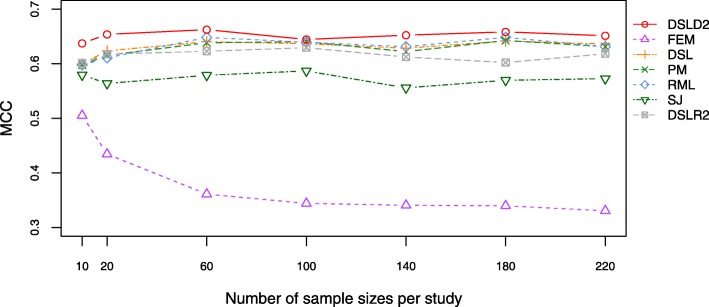
Plot of MCC under the first hypothesis. The DSLD2 method is developed in this paper. The MCC value of the DSLD2 method is the highest among that of the 7 meta-analysis methods

The false positive rate (FPR) is the probability of falsely rejecting the null hypothesis of a test. Under the first hypothesis, DSLD2 had the highest FPR value when number of sample sizes per study was 10 (see Fig. 5 and Additional file 4: Table S7). However, the FPR value of DSLD2 method went down to 0.0 when numbers of sample sizes per study were larger than 60 under the first hypothesis (see Fig. 4 and Additional file 4: Table S7). Under the first hypothesis, the DSLR2 method had the highest FPR values when numbers of sample sizes per study were more than 60 (see Fig. 4 and Additional file 4: Table S7). Under the second and the third hypothesis, the FEM method had the lowest FPR values among 7 meta-analysis methods (see Additional file 3: Figures S5, S6 and Additional file 4: Tables S8, S9).

The Matthews correlation coefficient (MCC), a numerical measure of correlation, indicates a statistical relationship between the predicted and observed binary classifications. An MCC close to 1 denotes perfect prediction. Under the first hypothesis, the DSLD2 method had the highest MCC among the 7 meta-analysis methods based on effect sizes (see Fig. 5 and Additional file 4: Table S10). The FEM method had the lowest MCC values among the 7 meta-analysis methods under the first hypothesis (see Fig. 5 and Additional file 4: Table S10). Under the first hypothesis, the SJ method had the lowest MCC values among the 6 random-effects meta-analysis methods (see Fig. 5 and Additional file 4: Table S10). Under the second, the FEM method had the highest MCC values among the 7 meta-analysis methods (see Additional file 3: Figure S7 and Additional file 4: Table S11). Under the third hypothesis, the SJ method had the highest MCC values among 7 meta-analysis methods based on effect sizes when the numbers of sample sizes per study were between 60 and 220 (see Additional file 3: Figure S8 and Additional file 4: Table S12).

**Fig. 6:**
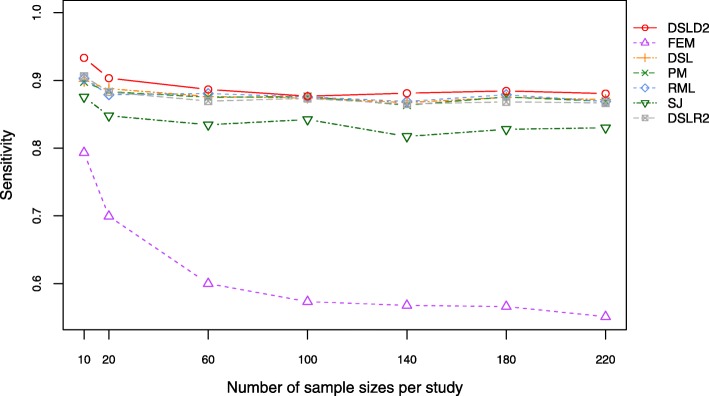
Plot of sensitivity under the first hypothesis. The DSLD2 method is developed in this paper. The sensitivity value of DSLD2 is the highest among that of the 7 meta-analysis methods

Sensitivity is a statistical measure of the performance of binary classification tests. Under the first hypothesis, the DSLD2 method had the highest sensitivity values among the 7 meta-analysis methods based on effect sizes (see Fig. 6 and Additional file 4: Table S13). The FEM method had the lowest sensitivity values among the 7 meta-analysis methods under the first hypothesis (see Fig. 6 and Additional file 4: Table S13). Under the second hypothesis, the 7 meta-analysis methods had close sensitivity curves (see Additional file 3: Figure S9 and Additional file 4: Table S14). Under the third hypothesis, the random-effect meta-analysis methods had close sensitivity curves which are higher than the curve of the FEM method. (see Additional file 3: Figure S10 and Additional file 4: Table S15).

**Fig. 7:**
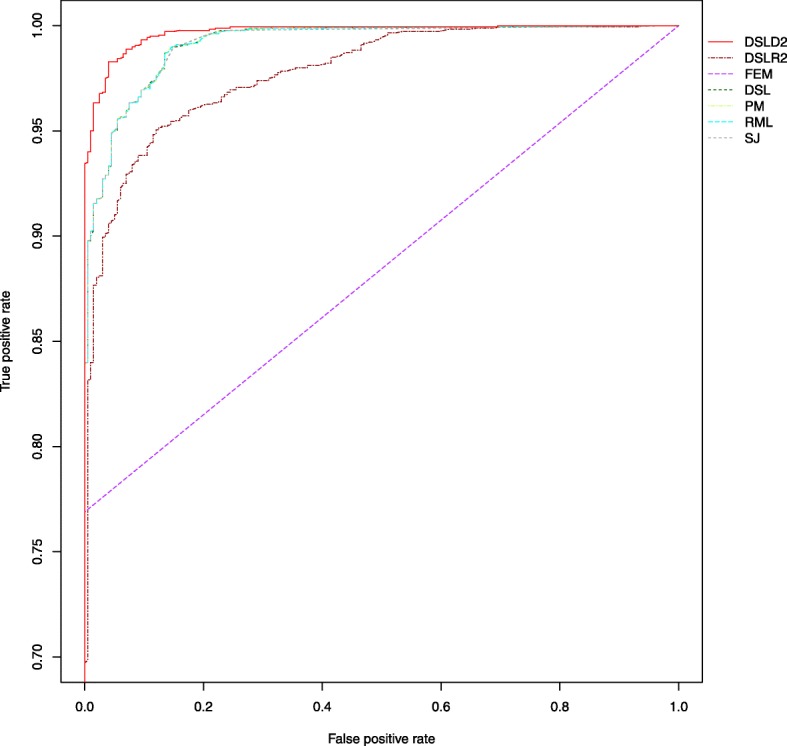
ROC curves of various meta-analysis methods under the first hypothesis. The DSLD2 method is developed in this paper. The ROC curve of DSLD2 is the highest among that of the 7 meta-analysis methods. The sample size of every study is 100

The receiver operating characteristic curve (ROC) is a tool for selecting possibly optimal models, and the area under the curve (AUC) measures how well two diagnostic results can be distinguished. *AUC*∈(0.9,1.0],*AUC*∈(0.7,0.9] and *AUC*∈(0.5,0.7] represent high, moderate and low accuracy, respectively. The DSLD2 method had AUC values of 0.996, 0.940 and 0.979 under the first, second, and third hypotheses, respectively. Under the first hypothesis, the DSLD2 method had the highest roc curve among all 7 meta-analysis methods (see Fig. 7). Under the second hypothesis, the roc curve of the DSLD2 method was the highest among 6 random-effects methods (see Additional file 3: Figure S11). Under the third hypothesis, the roc curve of the DSLD2 method was highest among the 7 meta-abnalysis methods based on effect sizes (see Additional file 3: Figure S12).

**Fig. 8:**
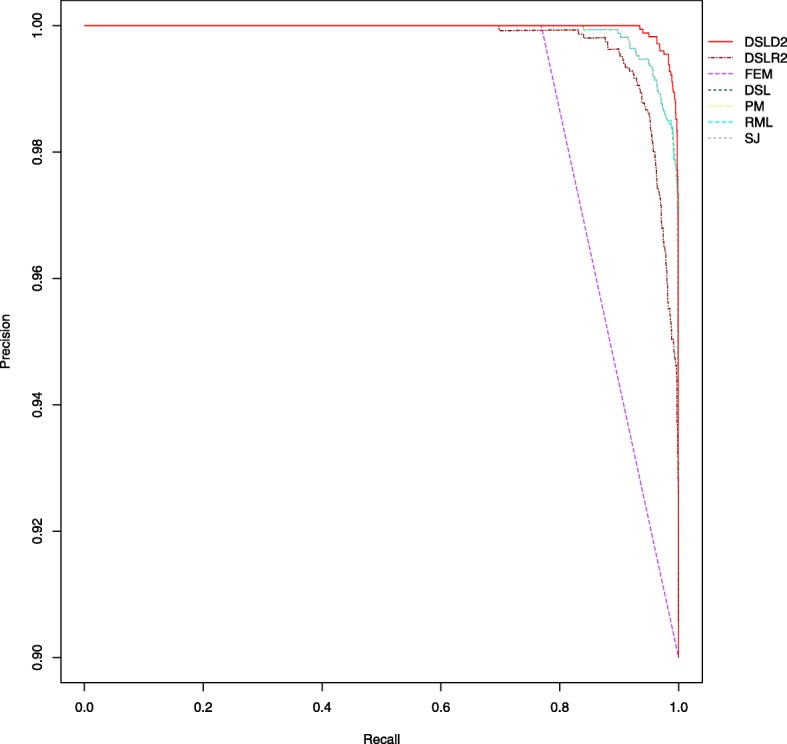
Precision-recall plot of various meta-analysis methods under the first hypothesis. The DSLD2 method is developed in this paper. The precision-recall curve of DSLD2 is the highest among that of 7 meta-analysis methods. The sample size of every study is 100

When the labels are highly imbalanced, ROC-AUC may give pretty good results and be misleading. Precision-recall plots could provide the researcher with a more accurate prediction because they evaluate the proportion of true positives among positive predictions [32]. Under the first hypothesis, the precision-recall curve of DSLD2 method was the highest among seven meta-analysis methods (see Fig. 8). The precision-recall curves of FEM and DSLR2 were lower than other curves under the first hypothesis (see Fig. 8). The DSL, PM, RML and SJ methods had almost the same precision-recall curve under the first hypothesis (see Fig. 8). Under the second hypothesis, the precision-recall curve of DSLD2 method was the highest among the curves of random-effects meta-analysis methods (see Additional file 3: Figure S13). The random-effects meta-analysis methods had close precision-recall curves which are lower than the curve of FEM under the third hypothesis (see Additional file 3: Figure S14).

**Fig. 9:**
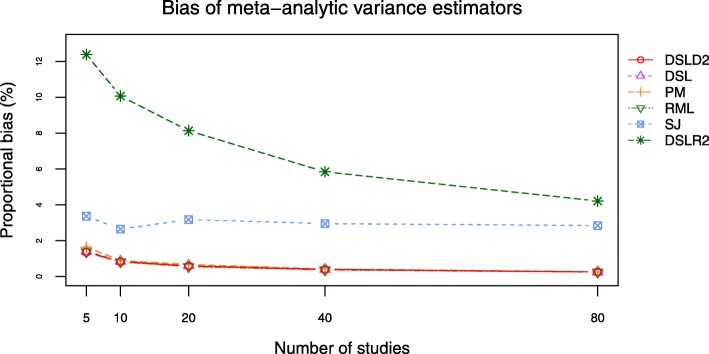
Bias plot of 6 meta-analysis methods when *τ*^2^ is set to 0.0 and SMD is chosen as the effect size measure

**Fig. 10:**
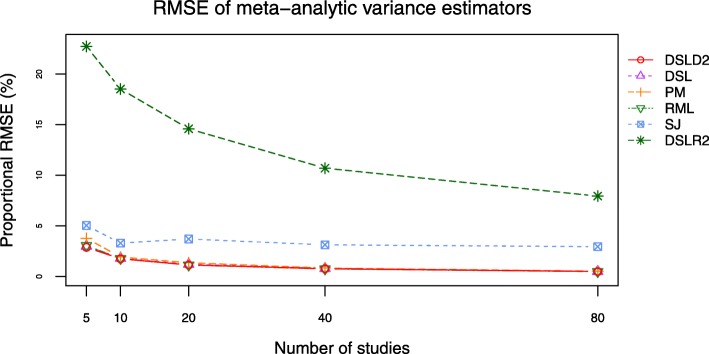
RMSE plot of 6 meta-analysis methods when *τ*^2^ is set to 0.0 and SMD is chosen as the effect size measure

**Fig. 11:**
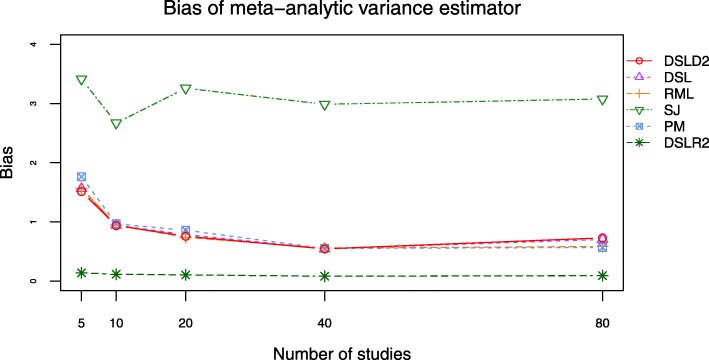
Bias plot of 6 meta-analysis methods when *τ*^2^ is set to 0.0 and MD is chosen as the effect size measure

**Fig. 12:**
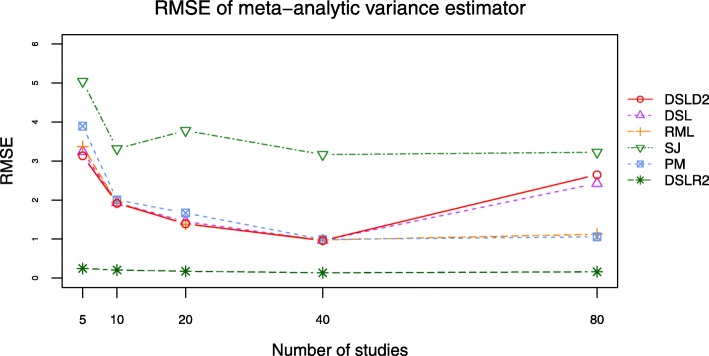
RMSE plot of 6 meta-analysis methods when *τ*^2^ is set to 0.0 and MD is chosen as the effect size measure

Bias and root mean square error (RMSE) are outcomes directly related to the between-study variance estimator $D^{2}_{g}$. The DSLD2, DSL, PM and RML methods had close bias and RMSE curves when *τ*^2^ was set to 0.0 and 1.0 (see Figs. 9, 10, 11 and 12, Additional file 3: Figures S15–S18 and Additional file 4: Tables S16-S23). The bias and RMSE curves of DSLD2, DSL, PM and RML methods were lower than that of SJ and DSLR2 methods when SMD was chosen as the effect size measure and *τ*^2^ was set to 0.0 (see Figs. 9 and 10). The DLSR2 method had the lowest bias and RMSE curves when MD was chosen as the effect size measure and *τ*^2^ was set to 0.0 (see Figs. 11 and 12). The bias and RMSE values of DSLD2, DSL, PM and RML methods were lower than that of the SJ method when MD was chosen as the effect size measure and *τ*^2^ was set to 0.0 (see Figs. 11 and 12). The DSLD2, DSL, PM and RML methods had the close bias and RMSE curves when the between study variance was set to 1.0 (Additional file 3: See Additional file 3: Figures S15–S18).

**Fig. 13:**
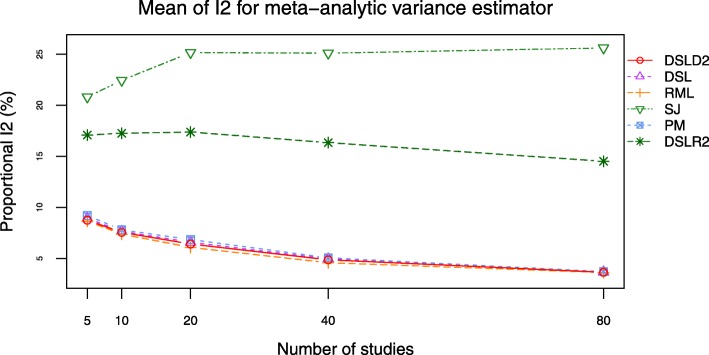
Mean of *I*^2^ plot of 6 meta-analysis methods when *τ*^2^ is set to 0.0 and SMD is chosen as the effect size measure

**Fig. 14:**
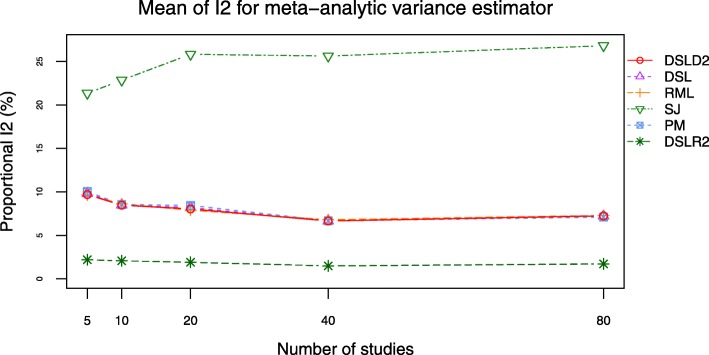
Mean of *I*^2^ plot of 6 meta-analysis methods when *τ*^2^ is set to 0.0 and MD is chosen as the effect size measure

The DSLD2, DSL, PM and RML methods had close mean values of *I*^2^ (see Figs. 13 and 14, Additional file 3: Figures S19–S20 and Additional file 4: Tables S24-S27). The *I*^2^ curves of DSLD2, DSL, PM and RML methods were lower than that of DSLR2 and SJ methods when SMD was chosen as the effect size measure (see Fig. 13 and Additional file 3: Figure S19). The *I*^2^ values of DSLD2, DSL, PM and RML methods were higher than that of DSLR2 method when MD was chosen as the effect size measure (see Fig. 14 and Additional file 3: Figure S20). The SJ method had the highest *I*^2^ curves when MD was chosen as the effect size measure (see Fig. 14 and Additional file 3: Figure S20).

### Application in genomic data

#### Alzheimer’s gene expression datasets

Alzheimer’s disease (AD), a neurodegenerative disease, is common in elderly indiviuals [33]. The incidence of AD has increased and is increasingly diagnosed in younger individuals. However, the etiology of AD is still unknown [34]. In this section, we used the DSLD2 method to analyze Alzheimer’s disease from a genetic perspective. Seven public AD gene expression datasets of the hippocampus from postmortem brain samples were used in this paper. The phenotypic and gene expression data are available through GEO accession numbers GSE36980 [35], GSE29378 [36], GSE84422 [37], GSE1297 [38], GSE5281 [39–41], GSE28146 and GSE48350 [42–48]. After within-study data preprocessing, filtering out genes with very low gene expression and excluding small variation genes, the meta-analysis of the DSLD2 method was conducted on 3257 target genes in 305 subjects (168 AD and 137 controls).

**Fig. 15:**
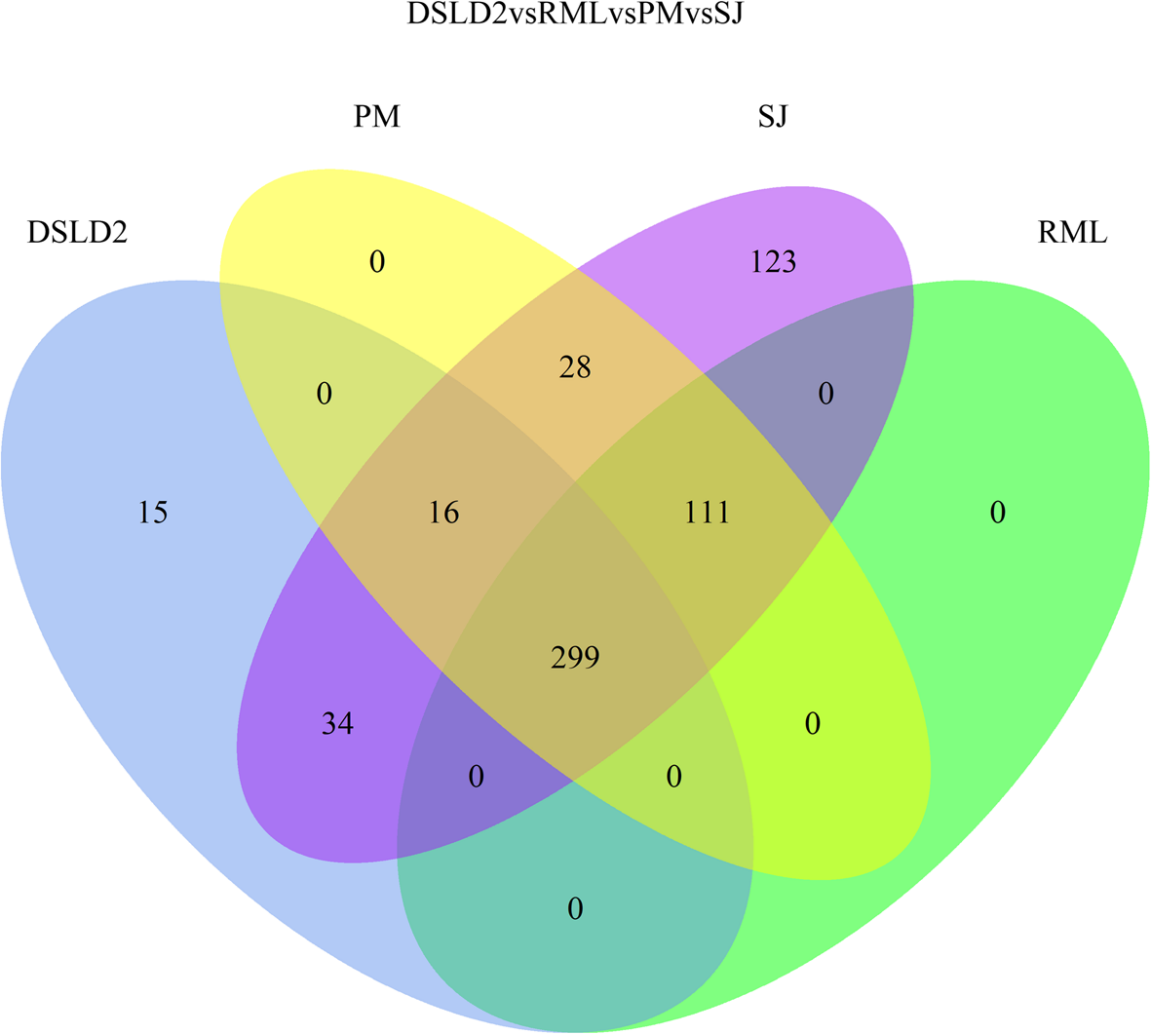
Venn diagram of differentially expressed genes detected by the DSLD2 (introduced in this paper), PM, SJ, and RML methods

A Venn diagram was plotted to compare DE genes (*p*<0.01) detected by the DSLD2, PM, SJ and RML methods. The DSLD2, PM, SJ and RML methods identified 364, 454, 611, 410 significantly DE genes (*p*<0.01), respectively (Fig. 15). The four meta-analysis methods found 299 overlapping DE genes. The DE genes detected by the DSLD2 method were different from DE genes identified by the PM, SJ and RML methods.

To biologically annotate the differentially expressed genes identified by DSLD2, the Kyoto Encyclopedia of Genes and Genomes (KEGG) pathway analysis was performed using over-representation analysis (ORA), and the first ten pathways are listed in Table 2. The differentially expressed genes with *p*<0.001 were significantly enriched in the neurological disease pathways, including the MAPK signaling pathway (hsa04010), the ErbB signaling pathway (hsa04012), *Helicobacter pylori* infection-induced epithelial cell signaling (hsa05120) and the hippo signaling pathway (hsa04392). Many studies have shown that Alzheimer’s disease is closely related to the MAPK signaling pathway. For example, Eun Kyung and Eui-Ju reported that deviation from the control of the MAPK signaling pathway influenced the progression of Alzheimer’s disease [49]. ErbB, a key NRG1 receptor, plays a significant role in the development and plasticity of Alzheimer’s disease. Woo et al. showed that the upregulation of ErbB4 immunoreactivity implicates the development of AD pathology [50]. The relationship between *Helicobacter pylori* infection (Hp-I) and Alzheimer’s disease was investigated by histological diagnosis [51]. Studies have shown that the pathophysiology of AD is influenced by *Helicobacter pylori* infection through many mechanisms [51]. Many studies have suggested that Alzheimer’s disease is related to the hippo signaling pathway [52].

**Table 2:** Pathways of the differentially expressed genes discovered by the DSLD2 method

PathwayID	Pathway name	C	O	E	R	*P*-value	FDR
hsa05212	Pancreatic cancer	66	7	1.22	5.72	0.0002	0.060
hsa04010	MAPK signaling pathway	255	13	4.72	2.75	0.0008	0.062
hsa05200	Pathways in cancer	397	17	7.35	2.31	0.0009	0.062
hsa04012	ErbB signaling pathway	88	7	1.63	4.29	0.0011	0.062
hsa05131	*Shigellosis*	65	6	1.20	4.97	0.0012	0.062
hsa04962	Vasopressin-regulated water reabsorption	44	5	0.81	6.13	0.0012	0.062
hsa05120	Epithelial cell signaling in *Helicobacterpylori* infection	68	6	1.26	4.76	0.0015	0.066
hsa04392	Hippo signaling pathway	29	4	0.53	7.44	0.0018	0.066
hsa01522	Endocrine resistance	98	7	1.81	3.85	0.0021	0.066
hsa04520	Adherens junction	74	6	1.37	4.37	0.0023	0.066

Note: C represents the number of reference genes in the category, O represents the number of genes in the category and also in the gene set, E represents expected number in the category, and R represents the ratio of enrichment

## Discussion and conclusion

This paper proposed a meta-analysis method (DSLD2) based on new between-study variance estimator $D_{g}^{2}$. The biases and RMSE of $D_{g}^{2}$ were lowest among 6 meta analysis methods when *τ*^2^ was set to 0 and SMD was chosen as the effect size measure (see Figs. 9 and 10). The DSLD2, DSL, PM and RML methods had close bias and RMSE values when *τ*^2^ was set to 0 or 1 and SMD or MD was chosen as the effect size measure (see Figs. 9, 10, 11 and 12 and Additional file 3: Figures S15–S18). The *I*^2^ values of DSLD2, DSL, PM and RML methods were close when the *τ*^2^ is set to 0.0 and 1.0 (see Figs. 13 and 14 and Additional file 3: Figures S19–S20).

We applied 7 meta-analysis methods based on effect sizes to simulation datasets of gene expression levels and compared the performance between the DSLD2 method and the other meta-analysis models. The FDR_1_ values of DSLD2 were smaller than that of DSL, PM, FEM, RML and SJ methods (see Table 1). The DSLD2 method had the lowest FDR_2_ values among the 7 meta-analysis methods based on effect sizes (see Table 1).

Under the first hypothesis, the precision, accuracy, sensitivity, FPR and MCC of the DSLD2 method varied greatly from 10 to 20 samples but tended to be stable between 60 and 220 samples (see Figs. 2, 3, 4, 5 and 6). The accuracy, MCC, sensitivity, ROC and precision-recall curve of the DSLD2 method were the highest among the 7 meta-analysis methods (see Figs. 3, 5, 6, 7 and 8). The precision of DSLD2 method wen up to 1.0 when the number of sample sizes per study was larger than 60 (see Fig. 2). The FPR of DSLD2 method wen down to 0.0 when the number of sample sizes per study was larger than 60 (see Fig. 4). The FEM method had the lowest curves of precision, accuracy, sensitivity, FPR and MCC (see Figs. 2, 3, 4, 5 and 6). The curves of precision, accuracy, sensitivity, FPR and MCC for the SJ method was lowest among random-effects meta-analysis methods (see Figs. 2, 3, 4, 5 and 6). The results of this simulation show that DSLD2 is a suitable method for detecting differentially expressed genes under the first hypothesis.

Under the second hypothesis, the DSLD2 and DSLR2 methods had the highest sensitivity values of approximately 1.0 (see Additional file 3: Figure S9). The ROC curve and precision-recall curve of DSLD2 method were the highest among 6 random-effects methods (see Additional file 3: Figures S11 and S13). The FEM method had the highest values of the precision, accuracy, FPR and MCC among 7 meta-analysis methods based on effect sizes (see Additional file 3: Figures S1, S3, S5 and S7).

Under the third hypothesis, the DSLD2 method had the high sensitivity values of approximately 1.0 (see Additional file 3: Figure S10). The ROC curve and precision-recall curve of DSLD2 method were the highest among 6 random-effects methods (see Additional file 3: Figures S12 and S14). The SJ method had the highest values of accuracy and MCC when number of sample sizes per study was between 60 to 220 (see Additional file 3: Figures S4 and S8). The FEM method had the highest precision values and the lowest FPR values (see Additional file 3: Figures S2 and S6).

We also applied the DSLD2 method to microarray data of Alzheimer’s disease. The differentially expressed genes with *p*<0.01 were significantly enriched in the neurological disease pathways, including the MAPK signaling pathway, the ErbB signaling pathway, *Helicobacter pylori* infection-induced epithelial cell signaling and the hippo signaling pathway. Moreover, many previous studies suggest that Alzheimer’s disease is related to pathways that DSLD2 discovered [49–52].

## Supplementary information

**Additional file 1** Proofs. Additional file 1 proves that the between-study variance $D_{g}^{2}$ is greater than 0 and $D_{g}^{2}\left (au^{2}\right)$ increases with *τ*^2^.

**Additional file 2** Supplementary methods. Additional file 2 gives the calculation processes of the false discovery rate, the precision, the accuracy, the false positive rate, the sensitivity and the Matthews correlation coefficient.

**Additional file 3** Supplementary figures. **Figure S1** Plot of the precision under the second hypothesis. **Figure S2** Plot of the precision under the third hypothesis. **Figure S3** Plot of the accuracy under the second hypothesis. **Figure S4** Plot of the accuracy under the third hypothesis. **Figure S5** Plot of the FPR under the second hypothesis. **Figure S6** Plot of the FPR under the third hypothesis. **Figure S7** Plot of the MCC under the second hypothesis. **Figure S8** Plot of the MCC under the third hypothesis. **Figure S9** Plot of the sensitivity under the second hypothesis. **Figure S10** Plot of the sensitivity under the third hypothesis. **Figure S11** Plot of the ROC curve and the AUC value under the second hypothesis. **Figure S12** Plot of the ROC under the third hypothesis. The DSLD2 method is developed in this paper. **Figure S13** Precision-recall plot under the second hypothesis. **Figure S14** Precision-recall plot under the third hypothesis. **Figure S15** Bias plot of 6 meta-analysis methods when *τ*^2^ is set to 1.0 and SMD is chosen as the effect size measure. **Figure S16** RMSE plot of 6 meta-analysis methods when *τ*^2^ is set to 1.0 and SMD is chosen as the effect size measure. **Figure S17** Bias plot of 6 meta-analysis methods when *τ*^2^ is set to 1.0 and MD is chosen as the effect size measure. **Figure S18** RMSE plot of 6 meta-analysis methods when *τ*^2^ is set to 1.0 and MD is chosen as the effect size measure. **Figure S19** Mean of *I*^2^ plot of 6 meta-analysis methods when *τ*^2^ is set to 1.0 and SMD is chosen as the effect size measure. **Figure S20** Mean of *I*^2^ plot of 6 meta-analysis methods when *τ*^2^ is set to 1.0 and MD is chosen as the effect size measure.

**Additional file 4** Tables. Additional file 4 is the tables of the precision, accuracy, FPR, MCC and sensitivity under three hypothesis and tables of bias, RMSE and mean of *I*^2^.

**Additional file 5** The r code. Additional file 5 is the code of the simulation setting for gene expression levels, the code of the simulation data using Monte Carlo method and the R code of DSLD2 method.

## Data Availability

The simulation datasets has been made publicly available (https://github.com/andwisdom/A-novel-estimator-of-between-study-variance-in-random-effects-models). The code of simulation datasets is shared in the article’s supplementary information files. The AD datasets analysised during the current study are available under entry numbers GSE36980, GSE29378, GSE84422, GSE1297, GSE5281, GSE28146 and GSE48350 from the GEO database in NCBI (http://www.ncbi.nlm.gov/geo). The R code used in this manuscript is public available in Additional file 5.

## Abbreviations

AD: Alzheimer’s disease
AUC: The area under the receiver operating characteristic curve
DSL: DerSimonian and Laird estimate
DSLD2: Two-step estimation starting with the DSL estimate and the $D_{g}^{2}$ in the second step
DSLR2: Two-step estimation starting with the DSL estimate and the *R*^2^ in the second step
EBI: European Bioinformatics Institute
FDR: False discovery rate
FEM: Fixed-effects model
FPR: False positive rate
GEO: Gene Expression Omnibus database
*I*^2^: Heterogeneity statistic *I*^2^
PM: Paule and Mandel estimate
REM: Random-effects model
RML: Restricted maximum likelihood estimate
RMSE: Root mean square error
ROC: Receiver operating characteristic curve
SJ: Sidik and Jonkman estimate
