# Supplementary material for: A novel estimator of between-study variance in random-effects models
Source: BMC Genomics. 2020 Feb 11;21:149. doi: 10.1186/s12864-020-6500-9 (PMC7014785; doi:10.1186/s12864-020-6500-9)
Supplement: Supplementary file 1 — Additional file 1 Proofs. Additional file 1 proves that the between-study variance $D_{g}^{2}$Dg2 is greater than 0 and $D_{g}^{2}\left (au^{2}\right)$Dg2τ2 increases with τ2. [file 12864_2020_6500_MOESM1_ESM.docx]

Additional file 1: Proofs

Nan Wang^1†^, Jun Zhang^2†^, Li Xu^3†^, Jing Qi^1^, Beibei Liu^1^, Yiyang Tang^4^, Yinan Jiang^5^, Liang Cheng^6^, Qinghua Jiang^7^, Xunbo Yin^1^ and Shuilin Jin^1*^

1. Department of Mathematics, Harbin Institute of Technology, Harbin, Heilongjiang, China

2. College of Computer Science and Technology, Harbin Engineering University, Harbin, China

3. School of Life Science and Technology, Harbin Institute of Technology, Harbin, China

† Equally contributed to the work

* To whom all correspondence should be addressed

*Corresponding author:

Shuilin Jin

School of Mathematics, Harbin Institute of Technology, Harbin, Heilongjiang, China

E-mail: jinsl@hit.edu.cn

This paper proposed a new estimator of between-study variance ($D^{2}$) and proved that this estimator met the general conditions of the between-study variance. The between-study variance $D^{2}$ which indicates the strength of random effects is greater than 0. If $D^{2}$ is small, the random effect is weak because the reduction of the variance due to random effects is small. Furthermore, the random-effects model simplifies to the fixed-effects model if $D^{2}$ is close to 0. However, the value of $D^{2}$ is far away from 0 indicating that the variance of random effects is very large.

1. Proof that $D^{2}(\hat{\tau}^{2})$ increases with $\hat{\tau}^{2}$for the meta-analysis model defined in equation (3).

Now we prove that the derivative of $D^{2}(\hat{\tau}^{2})$ with respect to $\hat{\tau}^{2}$is greater than 0. The derivative of $D^{2}(\hat{\tau}^{2})$ is

$$\begin{matrix} \frac{dD^{2}(\hat{\tau}^{2})}{d(\hat{\tau}^{2})}= -A\cdot\frac{dS_{MM}}{d(\hat{\tau}^{2})} \\ = -A\cdot\frac{d(\sum_{i=1}^{n} \frac{(y_{i}-M(\hat{\tau}^{2}))^{2}}{(\sigma_{i}^{2}+\hat{\tau}^{2})})}{d(\hat{\tau}^{2})} \\ = A\cdot\sum_{i=1}^{n} \frac{(y_{i}-M(\hat{\tau}^{2}))^{2}}{(\sigma_{i}^{2}+\hat{\tau}^{2})^{2}}+A\cdot\sum_{i=1}^{n} \frac{(y_{i}-M(\hat{\tau}^{2}))}{(\sigma_{i}^{2}+\hat{\tau}^{2})^{2}}\cdot\frac{dM(\hat{\tau}^{2})}{d(\hat{\tau}^{2})}, \end{matrix}$$

where $A=\frac{1}{\sum_{i=1}^{n} \frac{1}{\sigma_{i}^{2}}-\frac{\sum_{i=1}^{n} (1/\sigma_{i}^{2})^{2}}{\sum_{i=1}^{n} 1/\sigma_{i}^{2}}}>0.$ But the second term vanishes^[1]^. This implies that the derivative is nonnegative and $D^{2}(\hat{\tau}^{2})$ increases with $\hat{\tau}^{2}$.

1. Proof that $D^{2}\geq0$ in equation (3).

We know that $\frac{dS_{MM}}{d\hat{\tau}^{2}}\leq0$ from 1 and $S_{MM}(\hat{\tau}^{2})\leq S_{MM}(0)=S_{WLS}$.

So$D^{2}(\hat{\tau}^{2})=S_{WLS.}-S_{MM}(\hat{\tau}^{2})\geq0.$

**Reference**

1. Demidenko, E. *et al*. Random effects coefficient of determination for mixed and meta-analysis models. Commun Stat Theory Methods **41**, 953-969 (2012)
