# Supplementary material for: A novel estimator of between-study variance in random-effects models
Source: BMC Genomics. 2020 Feb 11;21:149. doi: 10.1186/s12864-020-6500-9 (PMC7014785; doi:10.1186/s12864-020-6500-9)
Supplement: Supplementary file 2 — Additional file 2 Supplementary methods. Additional file 2 gives the calculation processes of the false discovery rate, the precision, the accuracy, the false positive rate, the sensitivity and the Matthews correlation coefficient. [file 12864_2020_6500_MOESM2_ESM.docx]

Additional file 2

Nan Wang^1†^, Jun Zhang^2†^, Li Xu^3†^, Jing Qi^1^, Beibei Liu^1^, Yiyang Tang^4^, Yinan Jiang^5^, Liang Cheng^6^, Qinghua Jiang^7^, Xunbo Yin^1^ and Shuilin Jin^1*^

1. Department of Mathematics, Harbin Institute of Technology, Harbin, Heilongjiang, China

2. College of Computer Science and Technology, Harbin Engineering University, Harbin, China

3. School of Life Science and Technology, Harbin Institute of Technology, Harbin, China

† Equally contributed to the work

* To whom all correspondence should be addressed

*Corresponding author:

Shuilin Jin

School of Mathematics, Harbin Institute of Technology, Harbin, Heilongjiang, China

E-mail:jinsl@hit.edu.cn

**Evaluations of methods in simulation studies**

**False discovery rate**

Meta-analysis methods could be classified into three complementary hypothesis settings: the first hypothesis: identifying differentially expressed genes with non-zero effect sizes in all studies, the second hypothesis: identifying a differentially expressed gene in one or more studies and the third hypothesis: identifying a differentially expressed gene in all studies. Two types of were used to compare the performance of between DSLD2 methods and other meta-analysis models ([1]). targets on and targets on

where $I(.)$ denoted indicative function.

**Precision**

Precision was implemented to evaluate the power of tests and we also compared results of DSLD2 with the results in a previous study ([2]). Precision was significant genes correctly identified as being the truly differentially expressed genes over the total of significant genes:

$$\text{Precision}_{\text{The first hypothesis}}=\frac{\sum_{g} I(k_{g} =k\text{ and gene }g\text{ is detected})}{\text{\#\{genes detected\} }},$$

$$\text{Precision}_{\text{The second hypothesis}}=\frac{\sum_{g} I(k_{g} \geq r\text{ and gene }g\text{ is detected})}{\text{\#\{genes detected\} }},$$

$$\text{Precision}_{\text{The third hypothesis}}=\frac{\sum_{g} I(k_{g}>1\text{ and gene }g\text{ is detected})}{\text{\#\{genes detected\} }}.$$

where $k$ represents the number of studies.

**Accuracy**

We also measured accuracy of DSLD2 and other already existing meta-analysis methods ([3]). Accuracy was defined as the proportion of the significant genes and not significant genes correctly identified by meta-analysis methods over the total number of evaluated genes:

$$\text{Accuracy}_{ \text{The first hypothesis}}=\frac{\sum_{g} I(k_{g}=k\text{ and gene }g\text{ is detected})+\sum_{g} I(k_{g}<k\text{ and gene }g\text{ is not detected})}{\text{\#\{genes\} }},$$

$$\text{Accuracy}_{\text{The second hypothesis}}=\frac{\sum_{g} I(k_{g}\geq r\text{ and gene }g\text{ is detected})+\sum_{g} I(k_{g}\text{<}r\text{ and gene }g\text{ is not detected})}{\text{\#\{genes\} }}\text{, }$$

$$\text{Accuracy}_{\text{The third hypothesis}}=\frac{\sum_{g} I(k_{g}\geq\text{1 and gene }g\text{ is detected})+\sum_{g} I(k_{g}=0\text{ and gene }g\text{ is not detected})}{\text{\#\{genes\} }}.$$

**False positive rates**

We also compared the false positive rates (FPRs) of DSLD2 and other meta-analysis methods. The FPR was the proportion of not truly differentially expressed genes but identified as differentially expressed genes by meta-analysis methods over the total number of not truly DE genes ([4]):

$$\text{FPR}_{ \text{The first hypothesis}}=\frac{\sum_{g} I(k_{g}<k\text{ and gene }g\text{ is detected})}{\sum_{g} I(k_{g}<\mathrm{k})\text{ }},$$

$$\text{FPR}_{\text{The second hypothesis}}=\frac{\sum_{g} I(k_{g}<r\text{ and gene }g\text{ is detected})}{\sum_{g} I(k_{g}<\mathrm{k})\text{ }}\text{, and}$$

$$\text{FPR}_{\text{The third hypothesis}}=\frac{\sum_{g} I(k_{g}<0\text{ and gene }g\text{ is detected})}{\sum_{g} I(k_{g}<\mathrm{k})\text{ }}.$$

**Sensitivity**

Sensitivity (also called the true positive rate, or probability of detection in some fields) measured the proportion of significant genes correctly identified by meta-analysis methods over the total number of truly differentially expressed genes ([5]):

$$\text{Sensitivity}_{ \text{The first hypothesis}}=\frac{\sum_{g} I(k_{g}=k\text{ and gene }g\text{ is detected})}{\sum_{g} I(k_{g}=\mathrm{k})\text{ }},$$

$$\text{Sensitivity}_{\text{The second hypothesis}}=\frac{\sum_{g} I(k_{g}\geq r\text{ and gene }g\text{ is detected})}{\sum_{g} I(k_{g}\geq r)\text{ }}\text{, and}$$

$$\text{Sensitivity}_{\text{The third hypothesis}}=\frac{\sum_{g} I(k_{g}\geq\text{1 and gene }g\text{ is detected})}{\sum_{g} I(k_{g}\geq1)\text{ }}.$$

**Matthew’s correlation coefficient**

Matthew’s correlation coefficient (MCC) was also measured. The MCC is a correlation coefficient between the observed and predicted binary classifications and the MCC close to one represents a perfect prediction ([6]):

$$\text{MCC}_{ \text{The first hypothesis}}=\frac{\begin{matrix} \left\{\left\{\sum_{g}^{G} I(t_{g}=k\text{ and gene }g\text{ is detected})\right\}\cdot\left\{\sum_{g}^{G} I(t_{g}<k\text{ and gene }g\text{ is not detected})\right\}\right\} \\ -\left\{\left\{\sum_{g}^{G} I(t_{g}<k\text{ and gene }g\text{ is detected})\right\}\cdot\left\{\sum_{g}^{G} I(t_{g}=k\text{ and gene }g\text{ is not detected})\right\}\right\} \end{matrix}}{\sqrt{\sum_{g}^{G} I(t_{g}=\mathrm{k})\text{ }\sum_{g}^{G} I(t_{g}<\mathrm{k})\#\{\text{genes detected\} }\#\{\text{genes not detected\}}}},$$

$$\text{MCC}_{\text{The second hypothesis}}=\frac{\begin{matrix} \left\{\left\{\sum_{g}^{G} I(t_{g}\geq r\text{ and gene }g\text{ is detected})\right\}\cdot\left\{\sum_{g}^{G} I(t_{g}<r\text{ and gene }g\text{ is not detected})\right\}\right\} \\ -\left\{\left\{\sum_{g}^{G} I(t_{g}<r\text{ and gene }g\text{ is detected})\right\}\cdot\left\{\sum_{g}^{G} I(t_{g}\geq r\text{ and gene }g\text{ is not detected})\right\}\right\} \end{matrix}}{\sqrt{\sum_{g}^{G} I(t_{g}\geq r)\text{ }\sum_{g}^{G} I(t_{g}<r)\#\{\text{genes detected\} }\#\{\text{genes not detected\}}}}\text{, }$$

$$\text{MCC}_{\text{The third hypothesis}}=\frac{\begin{matrix} \left\{\left\{\sum_{g}^{G} I(t_{g}\geq\text{1 and gene }g\text{ is detected})\right\}\cdot\left\{\sum_{g}^{G} I(t_{g}=0\text{ and gene }g\text{ is not detected})\right\}\right\} \\ -\left\{\left\{\sum_{g}^{G} I(t_{g}=0\text{ and gene }g\text{ is detected})\right\}\cdot\left\{\sum_{g}^{G} I(t_{g}\geq\text{1 and gene }g\text{ is not detected})\right\}\right\} \end{matrix}}{\sqrt{\sum_{g}^{G} I(t_{g}\geq1)\text{ }\sum_{g}^{G} I(t_{g}=0)\#\{\text{genes detected\} }\#\{\text{genes not detected\}}}}.$$

**The receiver operating characteristic (ROC) curves and the resulting area under the curve (AUC)**

The ROC curve describes the ability of meta-analysis methods identifying differentially expressed genes with the change of the critical value and the best critical value can be chosen from the ROC curves. The area under the ROC curve (AUC) could evaluate the accuracy of the tests. The AUC measures how well a parameter can distinguish between two diagnostic groups, where $AUC\in(0.5,0.7],AUC\in(0.7,0.9]$ and $AUC\in(0.9,1.0]$ represent low, moderate and high accuracy, respectively ([6]).

**Reference**

1. Song, C. and Tseng, G C. Hypothesis setting and order statistic for robust genomic meta-analysis. Ann Appl Stat **8**, 777 (2014)
2. Law, C. W. *et al.* Voom: precision weights unlock linear model analysis tools for RNA-seq read counts. Genome Biol **15**, R29 (2014)
3. Swets, J. A. Measuring the accuracy of diagnostic systems. Science **240**, 1285–1293 (1988)
4. Matthews, B. W. Comparison of the predicted and observed secondary structure of T4 phage lysozyme. Biochimicaet Biophysica Acta (BBA) **405**, 442-451 (1975)
5. Siangphoe, U. and Archer, K. J. Estimation of random effects and identifying heterogeneous genes in meta-analysis of gene expression studies. Brief Bioinform **18**, 602-618 (2016)
6. Yang, X. *et al*. Similarities of ordered gene lists. J Bioinform Comput Biol **4**, 693–708 (2006)
