# Supplementary material for: A novel estimator of between-study variance in random-effects models
Source: BMC Genomics. 2020 Feb 11;21:149. doi: 10.1186/s12864-020-6500-9 (PMC7014785; doi:10.1186/s12864-020-6500-9)
Supplement: Supplementary file 3 — Additional file 3 Supplementary figures. Figure S1 Plot of the precision under the second hypothesis. Figure S2 Plot of the precision under the third hypothesis. Figure S3 Plot of the accuracy under the second hypothesis. Figure S4 Plot of the accuracy under the third hypothesis. Figure S5 Plot of the FPR under the second hypothesis. Figure S6 Plot of the FPR under the third hypothesis. Figure S7 Plot of the MCC under the second hypothesis. Figure S8 Plot of the MCC under the third hypothesis. Figure S9 Plot of the sensitivity under the second hypothesis. Figure S10 Plot of the sensitivity under the third hypothesis. Figure S11 Plot of the ROC curve and the AUC value under the second hypothesis. Figure S12 Plot of the ROC under the third hypothesis. The DSLD2 method is developed in this paper. Figure S13 Precision-recall plot under the second hypothesis. Figure S14 Precision-recall plot under the third hypothesis. Figure S15 Bias plot of 6 meta-analysis methods when τ2 is set to 1.0 and SMD is chosen as the effect size measure. Figure S16 RMSE plot of 6 meta-analysis methods when τ2 is set to 1.0 and SMD is chosen as the effect size measure. Figure S17 Bias plot of 6 meta-analysis methods when τ2 is set to 1.0 and MD is chosen as the effect size measure. Figure S18 RMSE plot of 6 meta-analysis methods when τ2 is set to 1.0 and MD is chosen as the effect size measure. Figure S19 Mean of I2 plot of 6 meta-analysis methods when τ2 is set to 1.0 and SMD is chosen as the effect size measure. Figure S20 Mean of I2 plot of 6 meta-analysis methods when τ2 is set to 1.0 and MD is chosen as the effect size measure. [file 12864_2020_6500_MOESM3_ESM.zip › Additional file 3.docx]

Additional file 3

Nan Wang^1†^, Jun Zhang^2†^, Li Xu^3†^, Jing Qi^1^, Beibei Liu^1^, Yiyang Tang^4^, Yinan Jiang^5^, Liang Cheng^6^, Qinghua Jiang^7^, Xunbo Yin^1^ and Shuilin Jin^1*^

1. Department of Mathematics, Harbin Institute of Technology, Harbin, Heilongjiang, China

2. College of Computer Science and Technology, Harbin Engineering University, Harbin, China

3. School of Life Science and Technology, Harbin Institute of Technology, Harbin, China

† Equally contributed to the work

* To whom all correspondence should be addressed

*Corresponding author:

Shuilin Jin

School of Mathematics, Harbin Institute of Technology, Harbin, Heilongjiang, China

E-mail:jinsl@hit.edu.cn

**Figure S1. Plot of precision under the second hypothesis.**

**Figure S2. Plot of precision under the third hypothesis.**

**Figure S3. Plot of accuracy under the second hypothesis.**

**Figure S4. Plot of accuracy under the third hypothesis.**

**Figure S5. Plot of FPR under the second hypothesis.**

**Figure S6. Plot of FPR under the third hypothesis.**

**Figure S7. Plot of MCC under the second hypothesis.**

**Figure S8. Plot of MCC under the third hypothesis.**

**Figure S9. Plot of sensitivity under the second hypothesis.**

**Figure S10. Plot of sensitivity under the third hypothesis.**

**Figure S11. Plots of ROC under the second hypothesis.**

**Figure S12. Plots of ROC under the third hypothesis.**

**Figure S13. Precision-recall plot under the second hypothesis.**

**Figure S14.** **Precision-recall plot under the third hypothesis.**

**Figure S15. Bias plot of 6 meta-analysis methods when** $\boldsymbol{\tau}^{\boldsymbol{2}}$ **is set to 1.0 and SMD is chosen as the effect size measure.**

**Figure S16. RMSE plot of 6 meta-analysis methods when** $\boldsymbol{\tau}^{\boldsymbol{2}}$ **is set to 1.0 and SMD is chosen as the effect size measure.**

**Figure S17. Bias plot of 6 meta-analysis methods when** $\boldsymbol{\tau}^{\boldsymbol{2}}$ **is set to 1.0 and MD is chosen as the effect size measure.**

**Figure S18. RMSE plot of 6 meta-analysis methods when** $\boldsymbol{\tau}^{\boldsymbol{2}}$ **is set to 1.0 and MD is chosen as the effect size measure.**

**Figure S19. Mean of** $\boldsymbol{I}^{\boldsymbol{2}}$ **plot of 6 meta-analysis methods when** $\boldsymbol{\tau}^{\boldsymbol{2}}$ **is set to 1.0 and SMD is chosen as the effect size measure.**

**Figure S20. Mean of** $\boldsymbol{I}^{\boldsymbol{2}}$ **plot of 6 meta-analysis methods when** $\boldsymbol{\tau}^{\boldsymbol{2}}$ **is set to 1.0 and MD is chosen as the effect size measure.**
