# Supplementary figures and images for: A novel estimator of between-study variance in random-effects models
Source: BMC Genomics. 2020 Feb 11;21:149. doi: 10.1186/s12864-020-6500-9 (PMC7014785; doi:10.1186/s12864-020-6500-9)

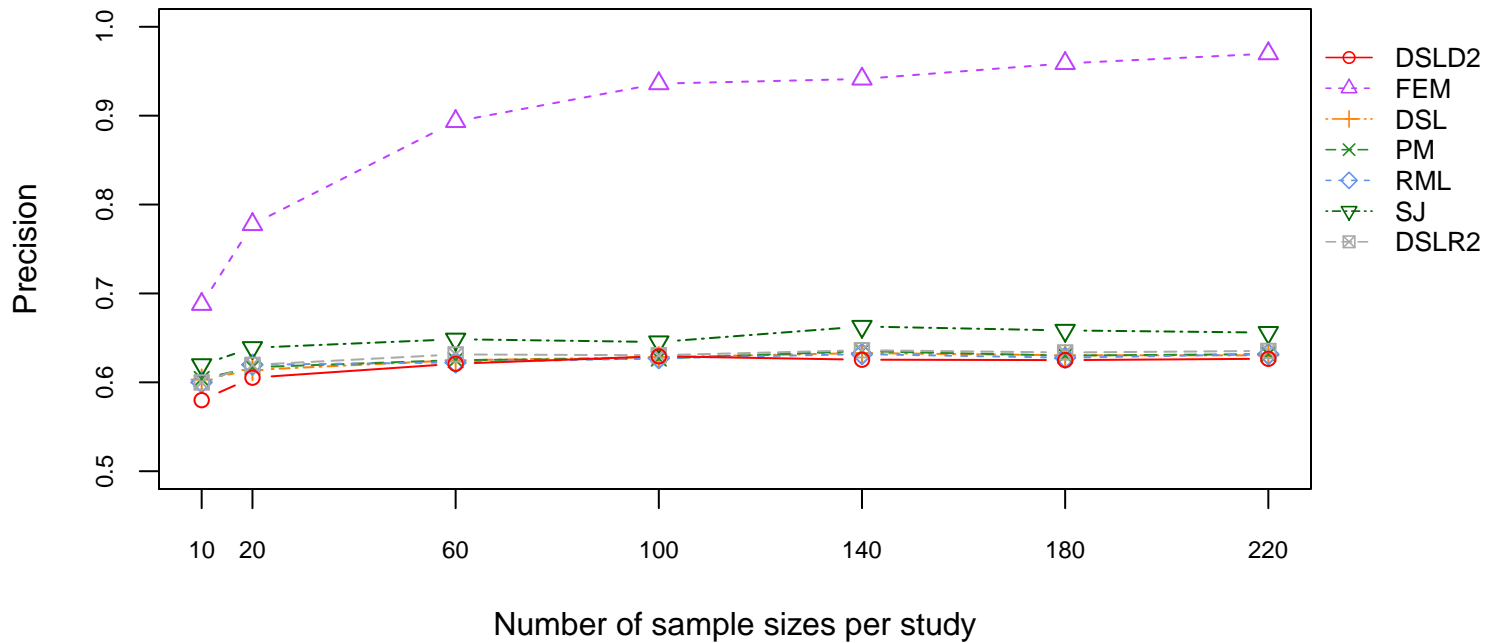

Supplement: Supplementary file 3 — Additional file 3 Supplementary figures. Figure S1 Plot of the precision under the second hypothesis. Figure S2 Plot of the precision under the third hypothesis. Figure S3 Plot of the accuracy under the second hypothesis. Figure S4 Plot of the accuracy under the third hypothesis. Figure S5 Plot of the FPR under the second hypothesis. Figure S6 Plot of the FPR under the third hypothesis. Figure S7 Plot of the MCC under the second hypothesis. Figure S8 Plot of the MCC under the third hypothesis. Figure S9 Plot of the sensitivity under the second hypothesis. Figure S10 Plot of the sensitivity under the third hypothesis. Figure S11 Plot of the ROC curve and the AUC value under the second hypothesis. Figure S12 Plot of the ROC under the third hypothesis. The DSLD2 method is developed in this paper. Figure S13 Precision-recall plot under the second hypothesis. Figure S14 Precision-recall plot under the third hypothesis. Figure S15 Bias plot of 6 meta-analysis methods when τ2 is set to 1.0 and SMD is chosen as the effect size measure. Figure S16 RMSE plot of 6 meta-analysis methods when τ2 is set to 1.0 and SMD is chosen as the effect size measure. Figure S17 Bias plot of 6 meta-analysis methods when τ2 is set to 1.0 and MD is chosen as the effect size measure. Figure S18 RMSE plot of 6 meta-analysis methods when τ2 is set to 1.0 and MD is chosen as the effect size measure. Figure S19 Mean of I2 plot of 6 meta-analysis methods when τ2 is set to 1.0 and SMD is chosen as the effect size measure. Figure S20 Mean of I2 plot of 6 meta-analysis methods when τ2 is set to 1.0 and MD is chosen as the effect size measure. [file 12864_2020_6500_MOESM3_ESM.zip › Figure S1.pdf]

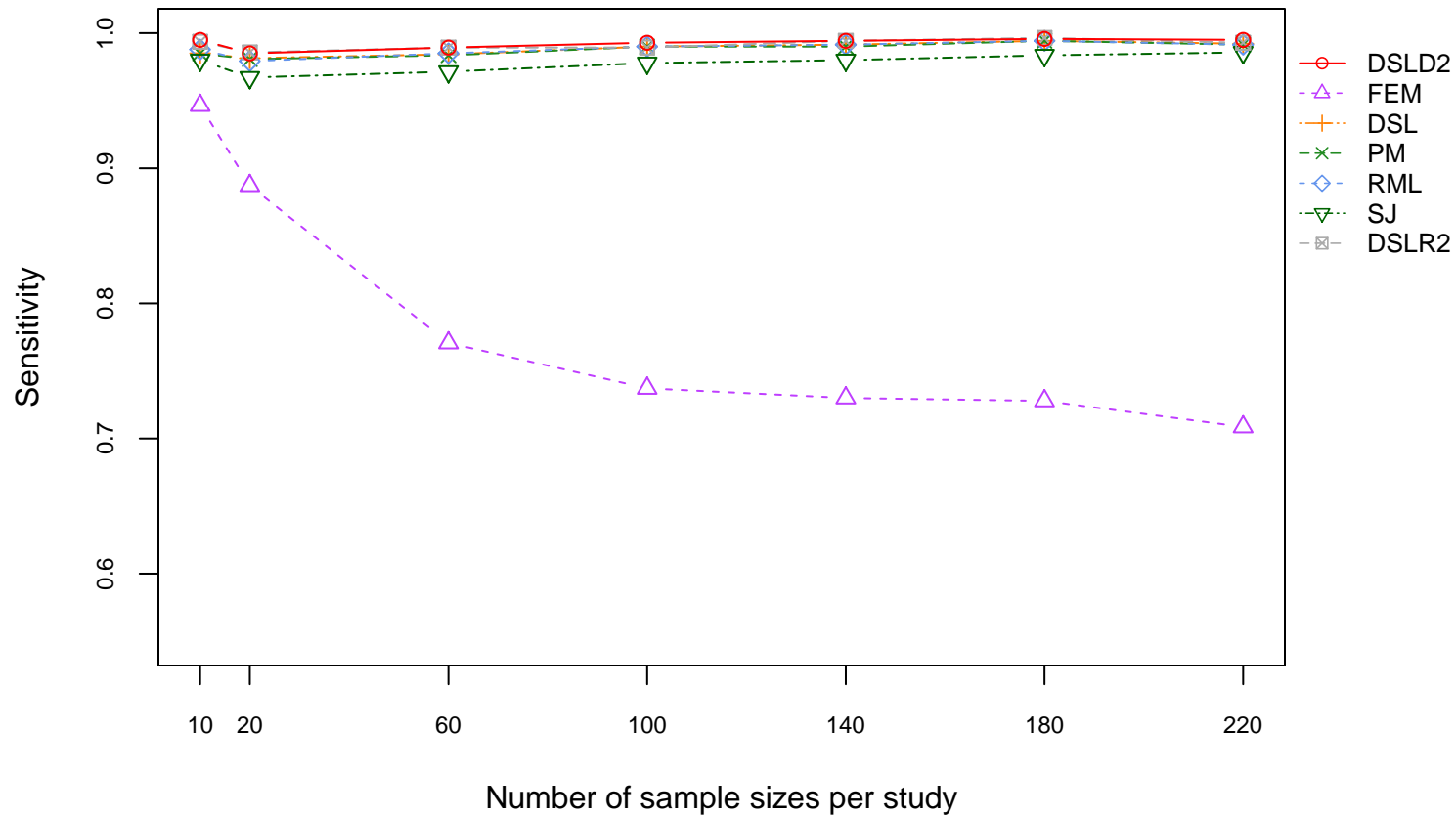

Supplement: Supplementary file 3 — Additional file 3 Supplementary figures. Figure S1 Plot of the precision under the second hypothesis. Figure S2 Plot of the precision under the third hypothesis. Figure S3 Plot of the accuracy under the second hypothesis. Figure S4 Plot of the accuracy under the third hypothesis. Figure S5 Plot of the FPR under the second hypothesis. Figure S6 Plot of the FPR under the third hypothesis. Figure S7 Plot of the MCC under the second hypothesis. Figure S8 Plot of the MCC under the third hypothesis. Figure S9 Plot of the sensitivity under the second hypothesis. Figure S10 Plot of the sensitivity under the third hypothesis. Figure S11 Plot of the ROC curve and the AUC value under the second hypothesis. Figure S12 Plot of the ROC under the third hypothesis. The DSLD2 method is developed in this paper. Figure S13 Precision-recall plot under the second hypothesis. Figure S14 Precision-recall plot under the third hypothesis. Figure S15 Bias plot of 6 meta-analysis methods when τ2 is set to 1.0 and SMD is chosen as the effect size measure. Figure S16 RMSE plot of 6 meta-analysis methods when τ2 is set to 1.0 and SMD is chosen as the effect size measure. Figure S17 Bias plot of 6 meta-analysis methods when τ2 is set to 1.0 and MD is chosen as the effect size measure. Figure S18 RMSE plot of 6 meta-analysis methods when τ2 is set to 1.0 and MD is chosen as the effect size measure. Figure S19 Mean of I2 plot of 6 meta-analysis methods when τ2 is set to 1.0 and SMD is chosen as the effect size measure. Figure S20 Mean of I2 plot of 6 meta-analysis methods when τ2 is set to 1.0 and MD is chosen as the effect size measure. [file 12864_2020_6500_MOESM3_ESM.zip › Figure S10.pdf]

True positive rate

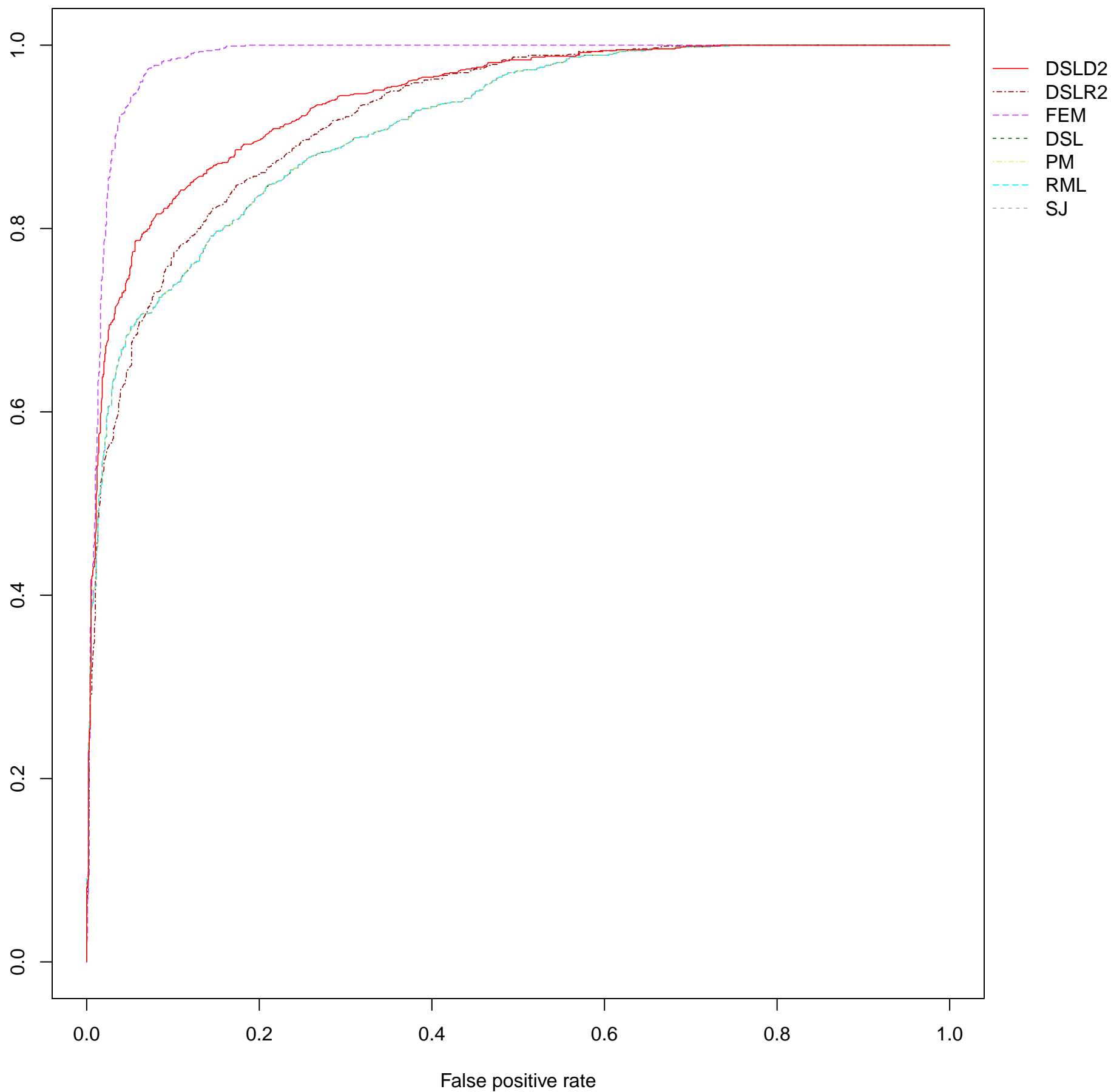

Supplement: Supplementary file 3 — Additional file 3 Supplementary figures. Figure S1 Plot of the precision under the second hypothesis. Figure S2 Plot of the precision under the third hypothesis. Figure S3 Plot of the accuracy under the second hypothesis. Figure S4 Plot of the accuracy under the third hypothesis. Figure S5 Plot of the FPR under the second hypothesis. Figure S6 Plot of the FPR under the third hypothesis. Figure S7 Plot of the MCC under the second hypothesis. Figure S8 Plot of the MCC under the third hypothesis. Figure S9 Plot of the sensitivity under the second hypothesis. Figure S10 Plot of the sensitivity under the third hypothesis. Figure S11 Plot of the ROC curve and the AUC value under the second hypothesis. Figure S12 Plot of the ROC under the third hypothesis. The DSLD2 method is developed in this paper. Figure S13 Precision-recall plot under the second hypothesis. Figure S14 Precision-recall plot under the third hypothesis. Figure S15 Bias plot of 6 meta-analysis methods when τ2 is set to 1.0 and SMD is chosen as the effect size measure. Figure S16 RMSE plot of 6 meta-analysis methods when τ2 is set to 1.0 and SMD is chosen as the effect size measure. Figure S17 Bias plot of 6 meta-analysis methods when τ2 is set to 1.0 and MD is chosen as the effect size measure. Figure S18 RMSE plot of 6 meta-analysis methods when τ2 is set to 1.0 and MD is chosen as the effect size measure. Figure S19 Mean of I2 plot of 6 meta-analysis methods when τ2 is set to 1.0 and SMD is chosen as the effect size measure. Figure S20 Mean of I2 plot of 6 meta-analysis methods when τ2 is set to 1.0 and MD is chosen as the effect size measure. [file 12864_2020_6500_MOESM3_ESM.zip › Figure S11.pdf]

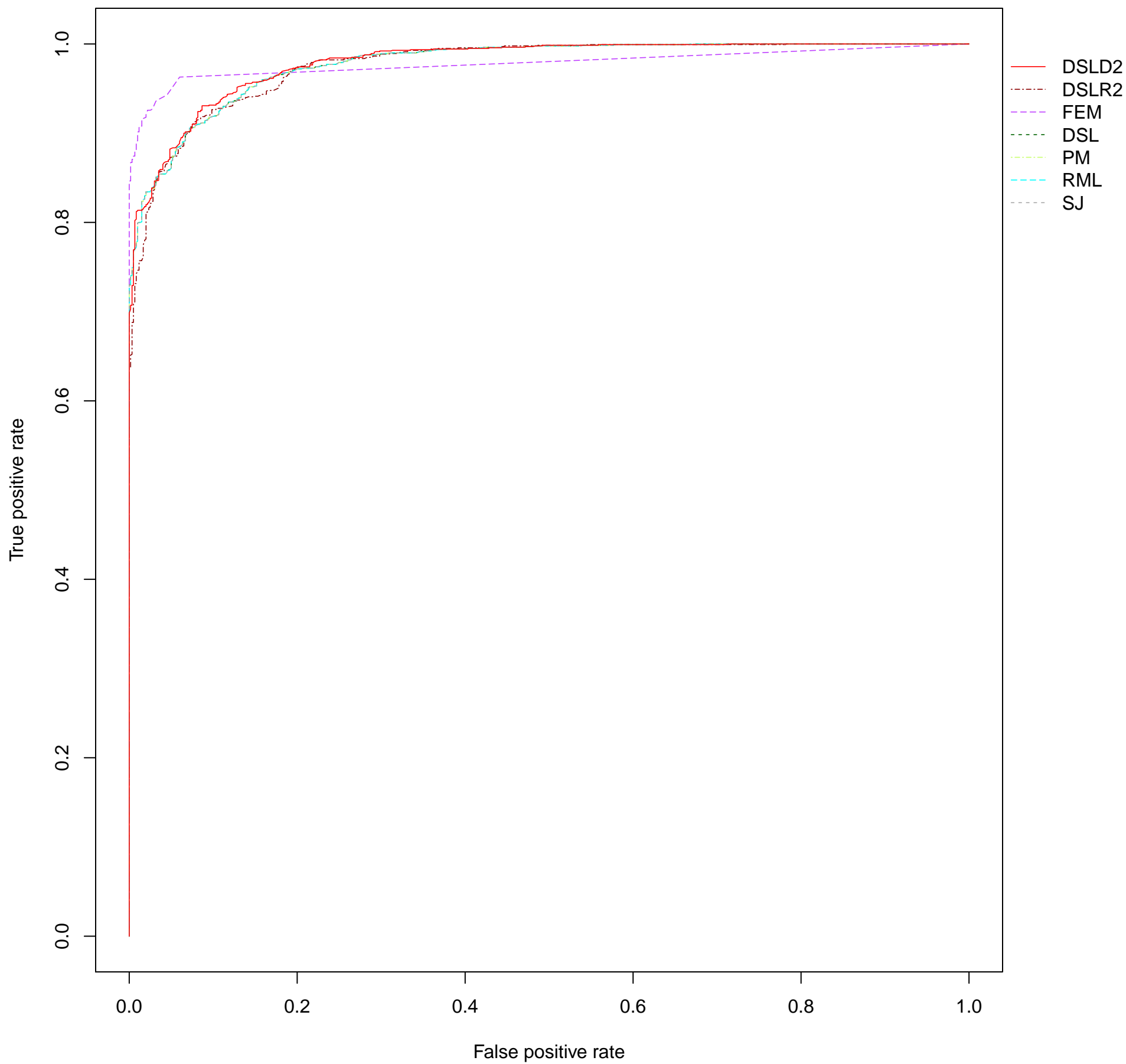

Supplement: Supplementary file 3 — Additional file 3 Supplementary figures. Figure S1 Plot of the precision under the second hypothesis. Figure S2 Plot of the precision under the third hypothesis. Figure S3 Plot of the accuracy under the second hypothesis. Figure S4 Plot of the accuracy under the third hypothesis. Figure S5 Plot of the FPR under the second hypothesis. Figure S6 Plot of the FPR under the third hypothesis. Figure S7 Plot of the MCC under the second hypothesis. Figure S8 Plot of the MCC under the third hypothesis. Figure S9 Plot of the sensitivity under the second hypothesis. Figure S10 Plot of the sensitivity under the third hypothesis. Figure S11 Plot of the ROC curve and the AUC value under the second hypothesis. Figure S12 Plot of the ROC under the third hypothesis. The DSLD2 method is developed in this paper. Figure S13 Precision-recall plot under the second hypothesis. Figure S14 Precision-recall plot under the third hypothesis. Figure S15 Bias plot of 6 meta-analysis methods when τ2 is set to 1.0 and SMD is chosen as the effect size measure. Figure S16 RMSE plot of 6 meta-analysis methods when τ2 is set to 1.0 and SMD is chosen as the effect size measure. Figure S17 Bias plot of 6 meta-analysis methods when τ2 is set to 1.0 and MD is chosen as the effect size measure. Figure S18 RMSE plot of 6 meta-analysis methods when τ2 is set to 1.0 and MD is chosen as the effect size measure. Figure S19 Mean of I2 plot of 6 meta-analysis methods when τ2 is set to 1.0 and SMD is chosen as the effect size measure. Figure S20 Mean of I2 plot of 6 meta-analysis methods when τ2 is set to 1.0 and MD is chosen as the effect size measure. [file 12864_2020_6500_MOESM3_ESM.zip › Figure S12.pdf]

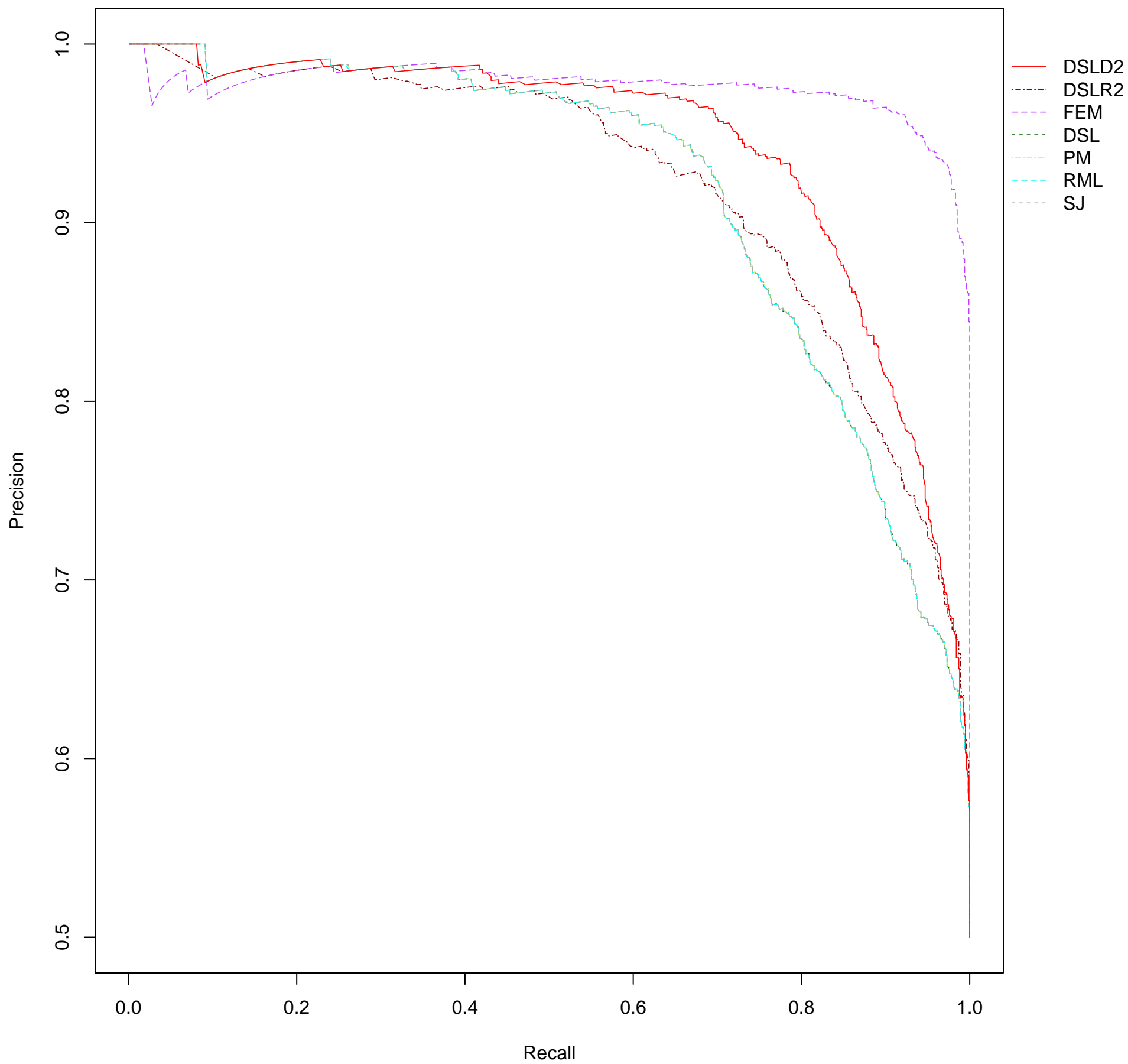

Supplement: Supplementary file 3 — Additional file 3 Supplementary figures. Figure S1 Plot of the precision under the second hypothesis. Figure S2 Plot of the precision under the third hypothesis. Figure S3 Plot of the accuracy under the second hypothesis. Figure S4 Plot of the accuracy under the third hypothesis. Figure S5 Plot of the FPR under the second hypothesis. Figure S6 Plot of the FPR under the third hypothesis. Figure S7 Plot of the MCC under the second hypothesis. Figure S8 Plot of the MCC under the third hypothesis. Figure S9 Plot of the sensitivity under the second hypothesis. Figure S10 Plot of the sensitivity under the third hypothesis. Figure S11 Plot of the ROC curve and the AUC value under the second hypothesis. Figure S12 Plot of the ROC under the third hypothesis. The DSLD2 method is developed in this paper. Figure S13 Precision-recall plot under the second hypothesis. Figure S14 Precision-recall plot under the third hypothesis. Figure S15 Bias plot of 6 meta-analysis methods when τ2 is set to 1.0 and SMD is chosen as the effect size measure. Figure S16 RMSE plot of 6 meta-analysis methods when τ2 is set to 1.0 and SMD is chosen as the effect size measure. Figure S17 Bias plot of 6 meta-analysis methods when τ2 is set to 1.0 and MD is chosen as the effect size measure. Figure S18 RMSE plot of 6 meta-analysis methods when τ2 is set to 1.0 and MD is chosen as the effect size measure. Figure S19 Mean of I2 plot of 6 meta-analysis methods when τ2 is set to 1.0 and SMD is chosen as the effect size measure. Figure S20 Mean of I2 plot of 6 meta-analysis methods when τ2 is set to 1.0 and MD is chosen as the effect size measure. [file 12864_2020_6500_MOESM3_ESM.zip › Figure S13.pdf]

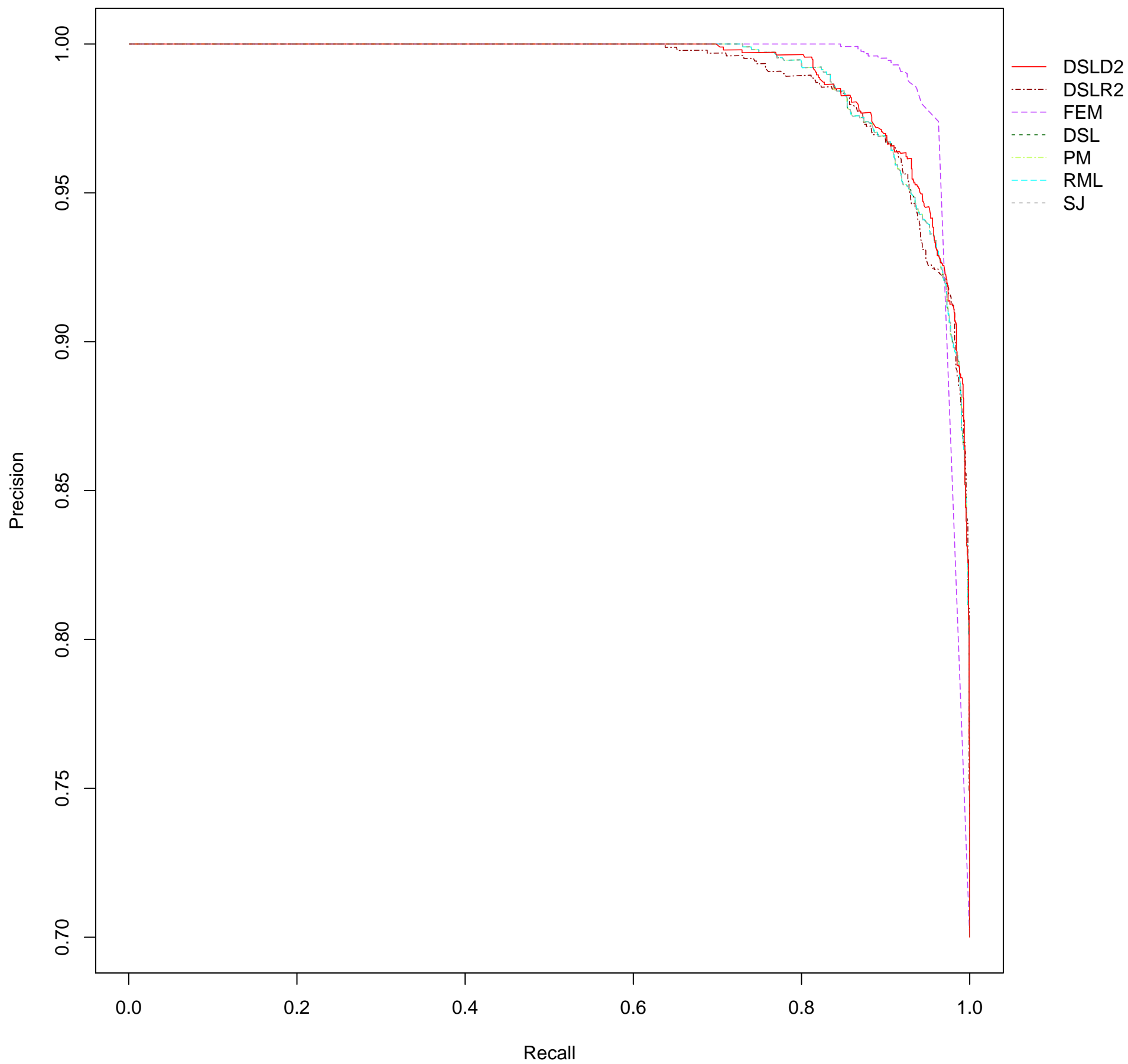

Supplement: Supplementary file 3 — Additional file 3 Supplementary figures. Figure S1 Plot of the precision under the second hypothesis. Figure S2 Plot of the precision under the third hypothesis. Figure S3 Plot of the accuracy under the second hypothesis. Figure S4 Plot of the accuracy under the third hypothesis. Figure S5 Plot of the FPR under the second hypothesis. Figure S6 Plot of the FPR under the third hypothesis. Figure S7 Plot of the MCC under the second hypothesis. Figure S8 Plot of the MCC under the third hypothesis. Figure S9 Plot of the sensitivity under the second hypothesis. Figure S10 Plot of the sensitivity under the third hypothesis. Figure S11 Plot of the ROC curve and the AUC value under the second hypothesis. Figure S12 Plot of the ROC under the third hypothesis. The DSLD2 method is developed in this paper. Figure S13 Precision-recall plot under the second hypothesis. Figure S14 Precision-recall plot under the third hypothesis. Figure S15 Bias plot of 6 meta-analysis methods when τ2 is set to 1.0 and SMD is chosen as the effect size measure. Figure S16 RMSE plot of 6 meta-analysis methods when τ2 is set to 1.0 and SMD is chosen as the effect size measure. Figure S17 Bias plot of 6 meta-analysis methods when τ2 is set to 1.0 and MD is chosen as the effect size measure. Figure S18 RMSE plot of 6 meta-analysis methods when τ2 is set to 1.0 and MD is chosen as the effect size measure. Figure S19 Mean of I2 plot of 6 meta-analysis methods when τ2 is set to 1.0 and SMD is chosen as the effect size measure. Figure S20 Mean of I2 plot of 6 meta-analysis methods when τ2 is set to 1.0 and MD is chosen as the effect size measure. [file 12864_2020_6500_MOESM3_ESM.zip › Figure S14.pdf]

Bias of meta-analytic variance estimator

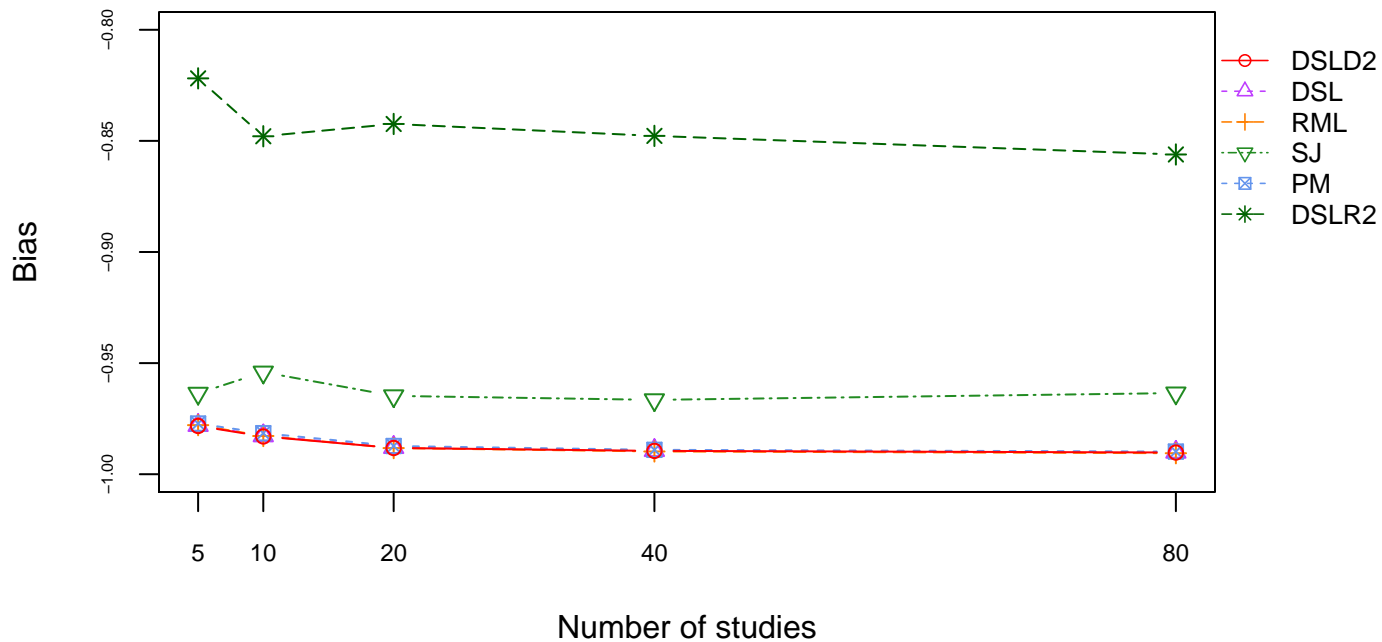

Supplement: Supplementary file 3 — Additional file 3 Supplementary figures. Figure S1 Plot of the precision under the second hypothesis. Figure S2 Plot of the precision under the third hypothesis. Figure S3 Plot of the accuracy under the second hypothesis. Figure S4 Plot of the accuracy under the third hypothesis. Figure S5 Plot of the FPR under the second hypothesis. Figure S6 Plot of the FPR under the third hypothesis. Figure S7 Plot of the MCC under the second hypothesis. Figure S8 Plot of the MCC under the third hypothesis. Figure S9 Plot of the sensitivity under the second hypothesis. Figure S10 Plot of the sensitivity under the third hypothesis. Figure S11 Plot of the ROC curve and the AUC value under the second hypothesis. Figure S12 Plot of the ROC under the third hypothesis. The DSLD2 method is developed in this paper. Figure S13 Precision-recall plot under the second hypothesis. Figure S14 Precision-recall plot under the third hypothesis. Figure S15 Bias plot of 6 meta-analysis methods when τ2 is set to 1.0 and SMD is chosen as the effect size measure. Figure S16 RMSE plot of 6 meta-analysis methods when τ2 is set to 1.0 and SMD is chosen as the effect size measure. Figure S17 Bias plot of 6 meta-analysis methods when τ2 is set to 1.0 and MD is chosen as the effect size measure. Figure S18 RMSE plot of 6 meta-analysis methods when τ2 is set to 1.0 and MD is chosen as the effect size measure. Figure S19 Mean of I2 plot of 6 meta-analysis methods when τ2 is set to 1.0 and SMD is chosen as the effect size measure. Figure S20 Mean of I2 plot of 6 meta-analysis methods when τ2 is set to 1.0 and MD is chosen as the effect size measure. [file 12864_2020_6500_MOESM3_ESM.zip › Figure S15.pdf]

RMSE of meta-analytic variance estimator

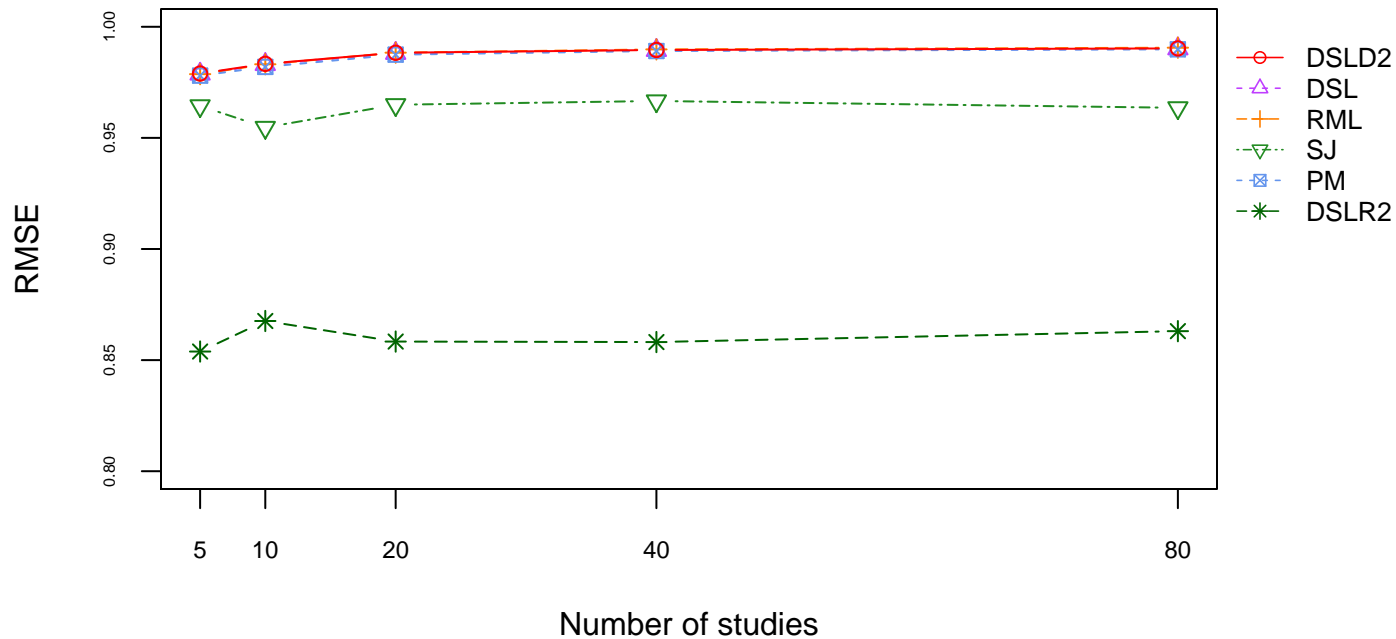

Supplement: Supplementary file 3 — Additional file 3 Supplementary figures. Figure S1 Plot of the precision under the second hypothesis. Figure S2 Plot of the precision under the third hypothesis. Figure S3 Plot of the accuracy under the second hypothesis. Figure S4 Plot of the accuracy under the third hypothesis. Figure S5 Plot of the FPR under the second hypothesis. Figure S6 Plot of the FPR under the third hypothesis. Figure S7 Plot of the MCC under the second hypothesis. Figure S8 Plot of the MCC under the third hypothesis. Figure S9 Plot of the sensitivity under the second hypothesis. Figure S10 Plot of the sensitivity under the third hypothesis. Figure S11 Plot of the ROC curve and the AUC value under the second hypothesis. Figure S12 Plot of the ROC under the third hypothesis. The DSLD2 method is developed in this paper. Figure S13 Precision-recall plot under the second hypothesis. Figure S14 Precision-recall plot under the third hypothesis. Figure S15 Bias plot of 6 meta-analysis methods when τ2 is set to 1.0 and SMD is chosen as the effect size measure. Figure S16 RMSE plot of 6 meta-analysis methods when τ2 is set to 1.0 and SMD is chosen as the effect size measure. Figure S17 Bias plot of 6 meta-analysis methods when τ2 is set to 1.0 and MD is chosen as the effect size measure. Figure S18 RMSE plot of 6 meta-analysis methods when τ2 is set to 1.0 and MD is chosen as the effect size measure. Figure S19 Mean of I2 plot of 6 meta-analysis methods when τ2 is set to 1.0 and SMD is chosen as the effect size measure. Figure S20 Mean of I2 plot of 6 meta-analysis methods when τ2 is set to 1.0 and MD is chosen as the effect size measure. [file 12864_2020_6500_MOESM3_ESM.zip › Figure S16.pdf]

Bias of meta-analytic variance estimator

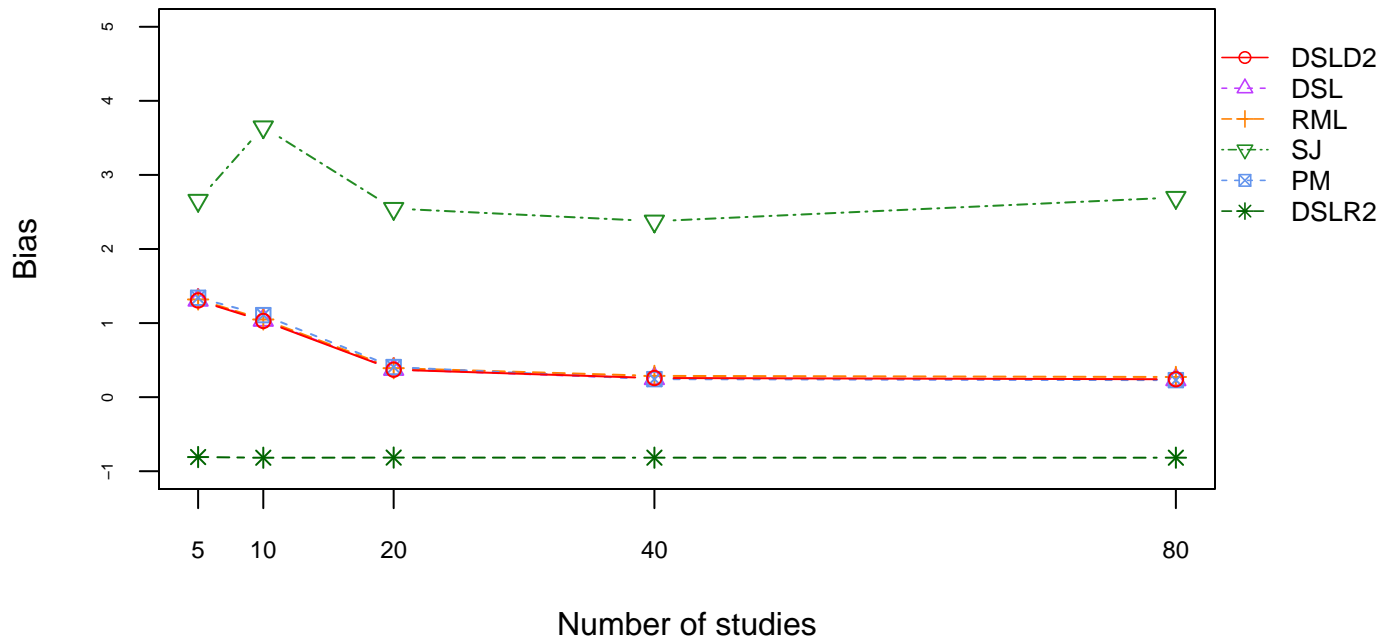

Supplement: Supplementary file 3 — Additional file 3 Supplementary figures. Figure S1 Plot of the precision under the second hypothesis. Figure S2 Plot of the precision under the third hypothesis. Figure S3 Plot of the accuracy under the second hypothesis. Figure S4 Plot of the accuracy under the third hypothesis. Figure S5 Plot of the FPR under the second hypothesis. Figure S6 Plot of the FPR under the third hypothesis. Figure S7 Plot of the MCC under the second hypothesis. Figure S8 Plot of the MCC under the third hypothesis. Figure S9 Plot of the sensitivity under the second hypothesis. Figure S10 Plot of the sensitivity under the third hypothesis. Figure S11 Plot of the ROC curve and the AUC value under the second hypothesis. Figure S12 Plot of the ROC under the third hypothesis. The DSLD2 method is developed in this paper. Figure S13 Precision-recall plot under the second hypothesis. Figure S14 Precision-recall plot under the third hypothesis. Figure S15 Bias plot of 6 meta-analysis methods when τ2 is set to 1.0 and SMD is chosen as the effect size measure. Figure S16 RMSE plot of 6 meta-analysis methods when τ2 is set to 1.0 and SMD is chosen as the effect size measure. Figure S17 Bias plot of 6 meta-analysis methods when τ2 is set to 1.0 and MD is chosen as the effect size measure. Figure S18 RMSE plot of 6 meta-analysis methods when τ2 is set to 1.0 and MD is chosen as the effect size measure. Figure S19 Mean of I2 plot of 6 meta-analysis methods when τ2 is set to 1.0 and SMD is chosen as the effect size measure. Figure S20 Mean of I2 plot of 6 meta-analysis methods when τ2 is set to 1.0 and MD is chosen as the effect size measure. [file 12864_2020_6500_MOESM3_ESM.zip › Figure S17.pdf]

RMSE of meta-analytic variance estimator

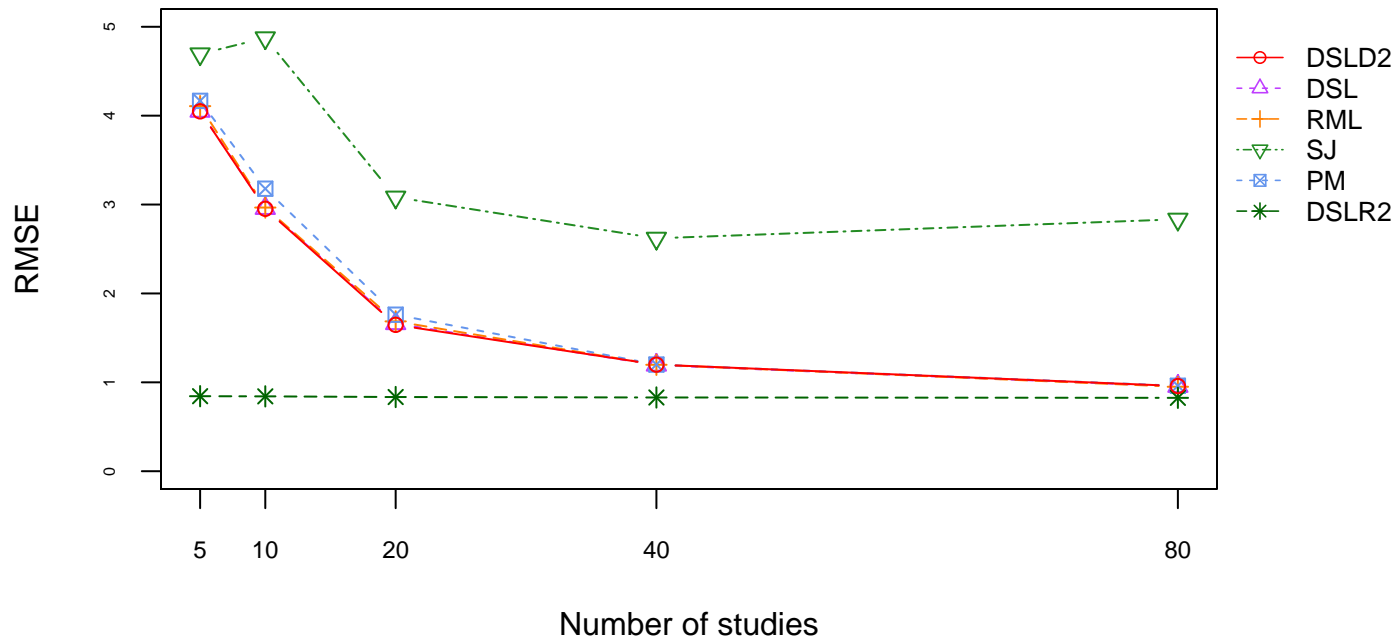

Supplement: Supplementary file 3 — Additional file 3 Supplementary figures. Figure S1 Plot of the precision under the second hypothesis. Figure S2 Plot of the precision under the third hypothesis. Figure S3 Plot of the accuracy under the second hypothesis. Figure S4 Plot of the accuracy under the third hypothesis. Figure S5 Plot of the FPR under the second hypothesis. Figure S6 Plot of the FPR under the third hypothesis. Figure S7 Plot of the MCC under the second hypothesis. Figure S8 Plot of the MCC under the third hypothesis. Figure S9 Plot of the sensitivity under the second hypothesis. Figure S10 Plot of the sensitivity under the third hypothesis. Figure S11 Plot of the ROC curve and the AUC value under the second hypothesis. Figure S12 Plot of the ROC under the third hypothesis. The DSLD2 method is developed in this paper. Figure S13 Precision-recall plot under the second hypothesis. Figure S14 Precision-recall plot under the third hypothesis. Figure S15 Bias plot of 6 meta-analysis methods when τ2 is set to 1.0 and SMD is chosen as the effect size measure. Figure S16 RMSE plot of 6 meta-analysis methods when τ2 is set to 1.0 and SMD is chosen as the effect size measure. Figure S17 Bias plot of 6 meta-analysis methods when τ2 is set to 1.0 and MD is chosen as the effect size measure. Figure S18 RMSE plot of 6 meta-analysis methods when τ2 is set to 1.0 and MD is chosen as the effect size measure. Figure S19 Mean of I2 plot of 6 meta-analysis methods when τ2 is set to 1.0 and SMD is chosen as the effect size measure. Figure S20 Mean of I2 plot of 6 meta-analysis methods when τ2 is set to 1.0 and MD is chosen as the effect size measure. [file 12864_2020_6500_MOESM3_ESM.zip › Figure S18.pdf]

Mean of I2 for meta-analytic variance estimator

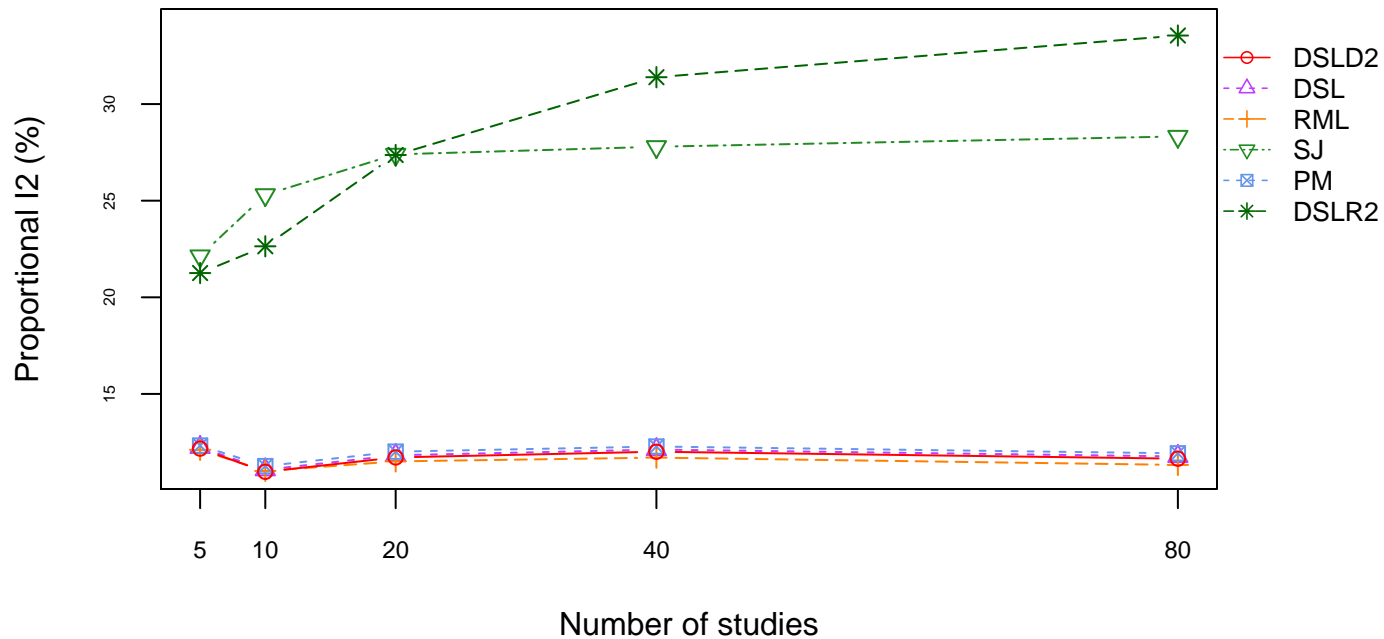

Supplement: Supplementary file 3 — Additional file 3 Supplementary figures. Figure S1 Plot of the precision under the second hypothesis. Figure S2 Plot of the precision under the third hypothesis. Figure S3 Plot of the accuracy under the second hypothesis. Figure S4 Plot of the accuracy under the third hypothesis. Figure S5 Plot of the FPR under the second hypothesis. Figure S6 Plot of the FPR under the third hypothesis. Figure S7 Plot of the MCC under the second hypothesis. Figure S8 Plot of the MCC under the third hypothesis. Figure S9 Plot of the sensitivity under the second hypothesis. Figure S10 Plot of the sensitivity under the third hypothesis. Figure S11 Plot of the ROC curve and the AUC value under the second hypothesis. Figure S12 Plot of the ROC under the third hypothesis. The DSLD2 method is developed in this paper. Figure S13 Precision-recall plot under the second hypothesis. Figure S14 Precision-recall plot under the third hypothesis. Figure S15 Bias plot of 6 meta-analysis methods when τ2 is set to 1.0 and SMD is chosen as the effect size measure. Figure S16 RMSE plot of 6 meta-analysis methods when τ2 is set to 1.0 and SMD is chosen as the effect size measure. Figure S17 Bias plot of 6 meta-analysis methods when τ2 is set to 1.0 and MD is chosen as the effect size measure. Figure S18 RMSE plot of 6 meta-analysis methods when τ2 is set to 1.0 and MD is chosen as the effect size measure. Figure S19 Mean of I2 plot of 6 meta-analysis methods when τ2 is set to 1.0 and SMD is chosen as the effect size measure. Figure S20 Mean of I2 plot of 6 meta-analysis methods when τ2 is set to 1.0 and MD is chosen as the effect size measure. [file 12864_2020_6500_MOESM3_ESM.zip › Figure S19.pdf]

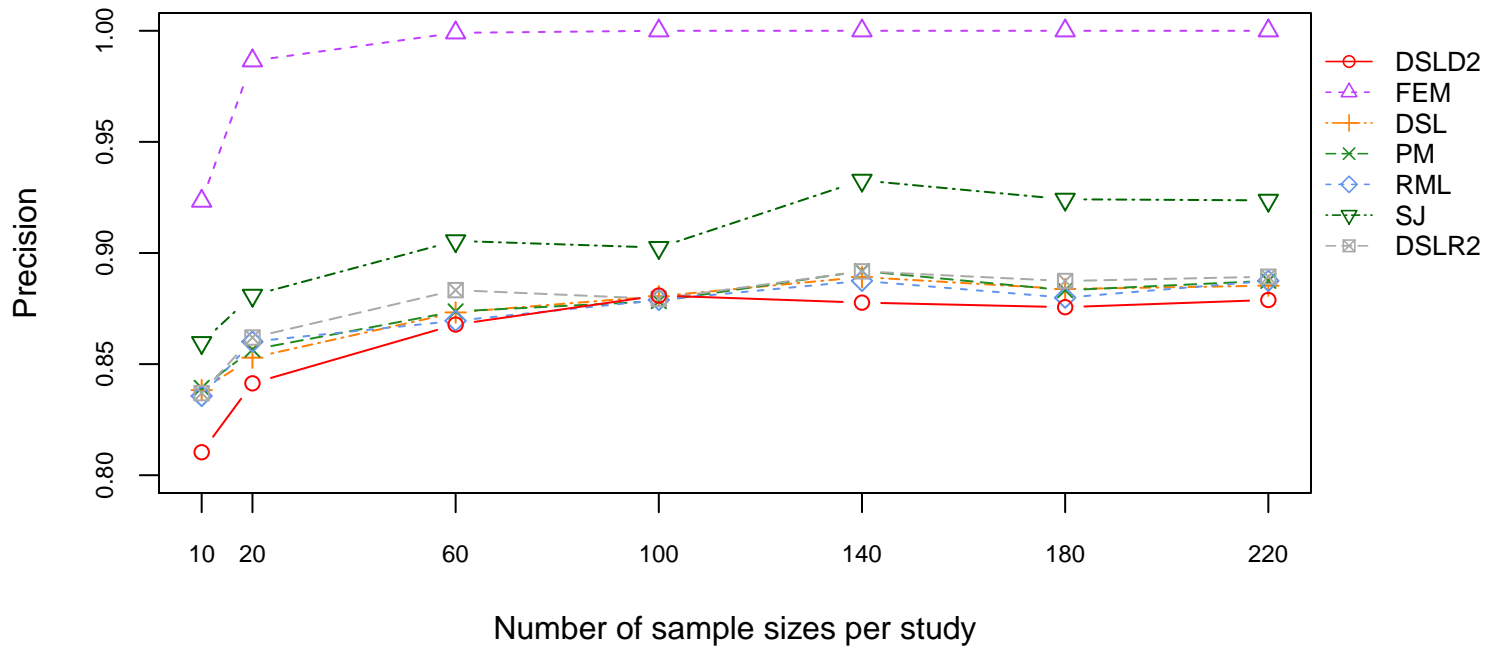

Supplement: Supplementary file 3 — Additional file 3 Supplementary figures. Figure S1 Plot of the precision under the second hypothesis. Figure S2 Plot of the precision under the third hypothesis. Figure S3 Plot of the accuracy under the second hypothesis. Figure S4 Plot of the accuracy under the third hypothesis. Figure S5 Plot of the FPR under the second hypothesis. Figure S6 Plot of the FPR under the third hypothesis. Figure S7 Plot of the MCC under the second hypothesis. Figure S8 Plot of the MCC under the third hypothesis. Figure S9 Plot of the sensitivity under the second hypothesis. Figure S10 Plot of the sensitivity under the third hypothesis. Figure S11 Plot of the ROC curve and the AUC value under the second hypothesis. Figure S12 Plot of the ROC under the third hypothesis. The DSLD2 method is developed in this paper. Figure S13 Precision-recall plot under the second hypothesis. Figure S14 Precision-recall plot under the third hypothesis. Figure S15 Bias plot of 6 meta-analysis methods when τ2 is set to 1.0 and SMD is chosen as the effect size measure. Figure S16 RMSE plot of 6 meta-analysis methods when τ2 is set to 1.0 and SMD is chosen as the effect size measure. Figure S17 Bias plot of 6 meta-analysis methods when τ2 is set to 1.0 and MD is chosen as the effect size measure. Figure S18 RMSE plot of 6 meta-analysis methods when τ2 is set to 1.0 and MD is chosen as the effect size measure. Figure S19 Mean of I2 plot of 6 meta-analysis methods when τ2 is set to 1.0 and SMD is chosen as the effect size measure. Figure S20 Mean of I2 plot of 6 meta-analysis methods when τ2 is set to 1.0 and MD is chosen as the effect size measure. [file 12864_2020_6500_MOESM3_ESM.zip › Figure S2.pdf]

Mean of I2 for meta-analytic variance estimator

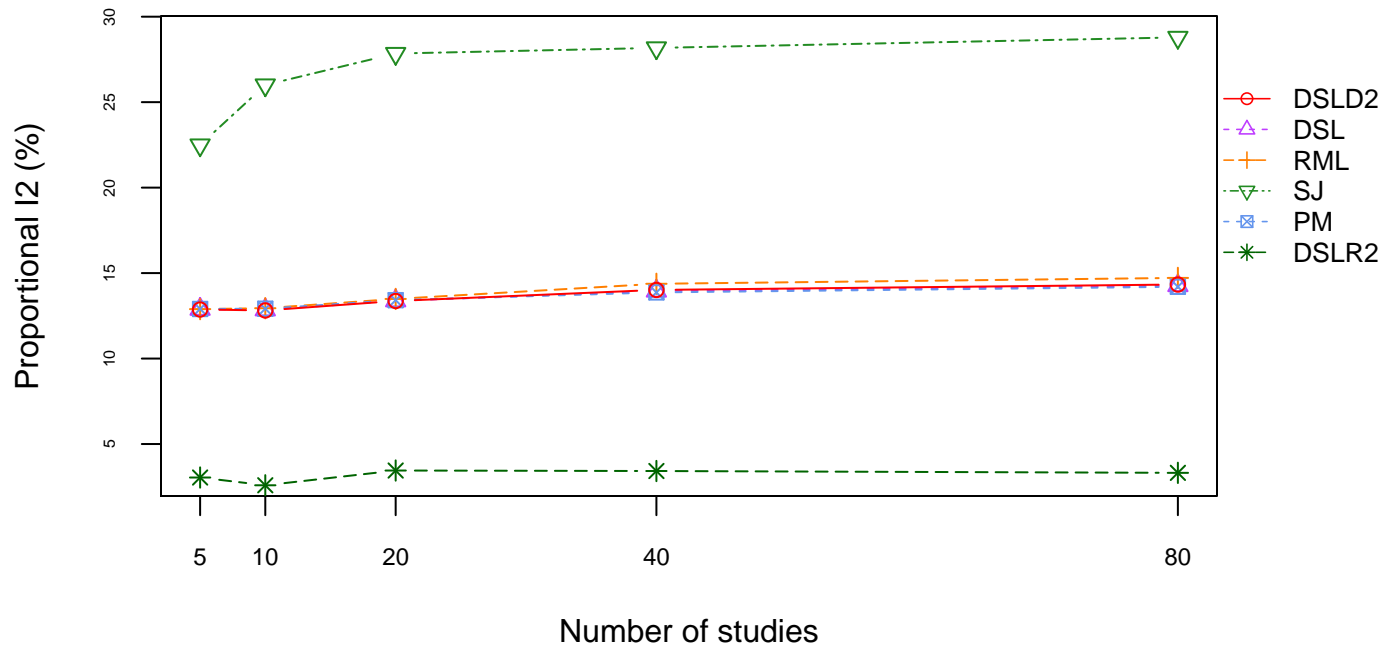

Supplement: Supplementary file 3 — Additional file 3 Supplementary figures. Figure S1 Plot of the precision under the second hypothesis. Figure S2 Plot of the precision under the third hypothesis. Figure S3 Plot of the accuracy under the second hypothesis. Figure S4 Plot of the accuracy under the third hypothesis. Figure S5 Plot of the FPR under the second hypothesis. Figure S6 Plot of the FPR under the third hypothesis. Figure S7 Plot of the MCC under the second hypothesis. Figure S8 Plot of the MCC under the third hypothesis. Figure S9 Plot of the sensitivity under the second hypothesis. Figure S10 Plot of the sensitivity under the third hypothesis. Figure S11 Plot of the ROC curve and the AUC value under the second hypothesis. Figure S12 Plot of the ROC under the third hypothesis. The DSLD2 method is developed in this paper. Figure S13 Precision-recall plot under the second hypothesis. Figure S14 Precision-recall plot under the third hypothesis. Figure S15 Bias plot of 6 meta-analysis methods when τ2 is set to 1.0 and SMD is chosen as the effect size measure. Figure S16 RMSE plot of 6 meta-analysis methods when τ2 is set to 1.0 and SMD is chosen as the effect size measure. Figure S17 Bias plot of 6 meta-analysis methods when τ2 is set to 1.0 and MD is chosen as the effect size measure. Figure S18 RMSE plot of 6 meta-analysis methods when τ2 is set to 1.0 and MD is chosen as the effect size measure. Figure S19 Mean of I2 plot of 6 meta-analysis methods when τ2 is set to 1.0 and SMD is chosen as the effect size measure. Figure S20 Mean of I2 plot of 6 meta-analysis methods when τ2 is set to 1.0 and MD is chosen as the effect size measure. [file 12864_2020_6500_MOESM3_ESM.zip › Figure S20.pdf]

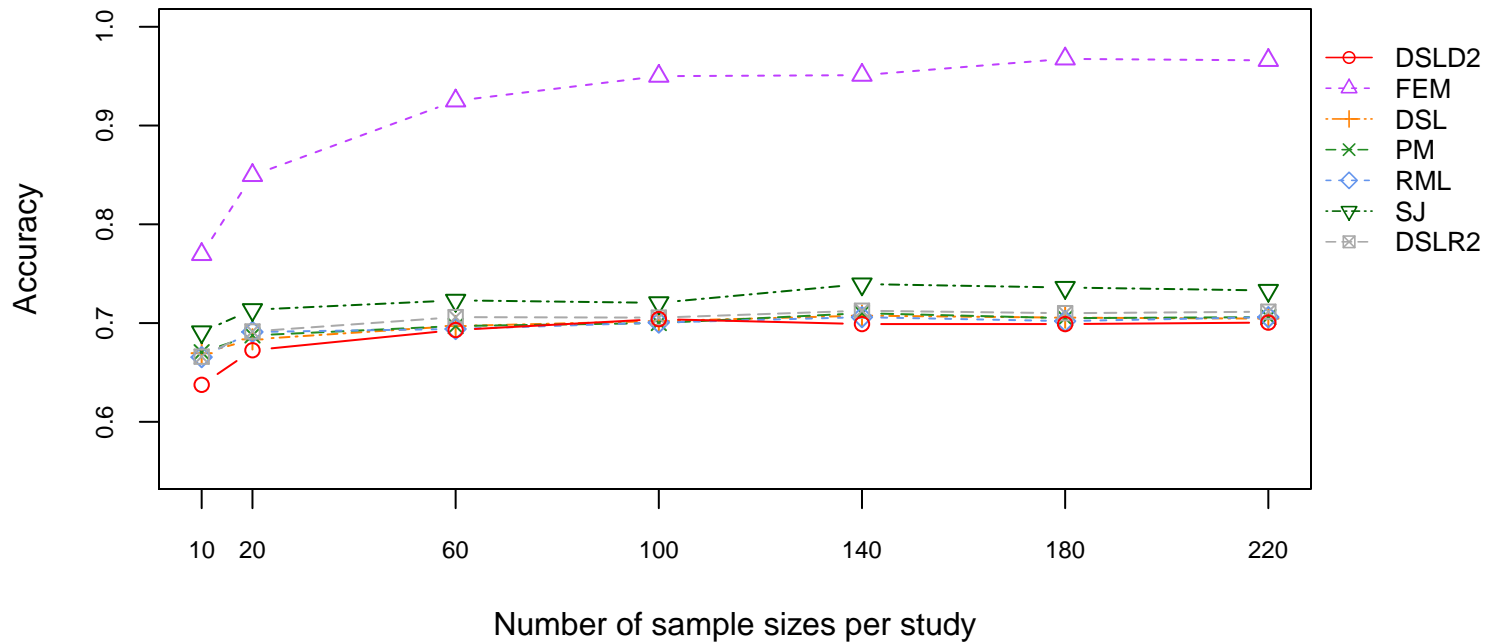

Supplement: Supplementary file 3 — Additional file 3 Supplementary figures. Figure S1 Plot of the precision under the second hypothesis. Figure S2 Plot of the precision under the third hypothesis. Figure S3 Plot of the accuracy under the second hypothesis. Figure S4 Plot of the accuracy under the third hypothesis. Figure S5 Plot of the FPR under the second hypothesis. Figure S6 Plot of the FPR under the third hypothesis. Figure S7 Plot of the MCC under the second hypothesis. Figure S8 Plot of the MCC under the third hypothesis. Figure S9 Plot of the sensitivity under the second hypothesis. Figure S10 Plot of the sensitivity under the third hypothesis. Figure S11 Plot of the ROC curve and the AUC value under the second hypothesis. Figure S12 Plot of the ROC under the third hypothesis. The DSLD2 method is developed in this paper. Figure S13 Precision-recall plot under the second hypothesis. Figure S14 Precision-recall plot under the third hypothesis. Figure S15 Bias plot of 6 meta-analysis methods when τ2 is set to 1.0 and SMD is chosen as the effect size measure. Figure S16 RMSE plot of 6 meta-analysis methods when τ2 is set to 1.0 and SMD is chosen as the effect size measure. Figure S17 Bias plot of 6 meta-analysis methods when τ2 is set to 1.0 and MD is chosen as the effect size measure. Figure S18 RMSE plot of 6 meta-analysis methods when τ2 is set to 1.0 and MD is chosen as the effect size measure. Figure S19 Mean of I2 plot of 6 meta-analysis methods when τ2 is set to 1.0 and SMD is chosen as the effect size measure. Figure S20 Mean of I2 plot of 6 meta-analysis methods when τ2 is set to 1.0 and MD is chosen as the effect size measure. [file 12864_2020_6500_MOESM3_ESM.zip › Figure S3.pdf]

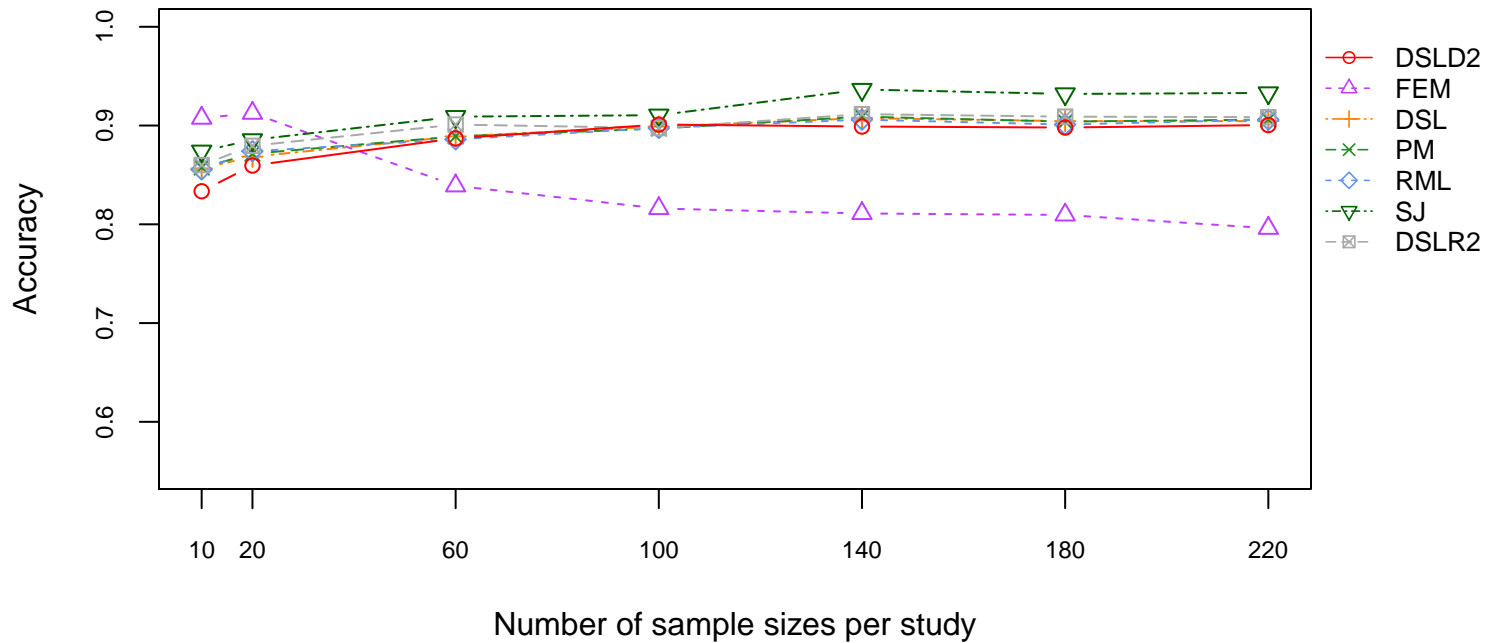

Supplement: Supplementary file 3 — Additional file 3 Supplementary figures. Figure S1 Plot of the precision under the second hypothesis. Figure S2 Plot of the precision under the third hypothesis. Figure S3 Plot of the accuracy under the second hypothesis. Figure S4 Plot of the accuracy under the third hypothesis. Figure S5 Plot of the FPR under the second hypothesis. Figure S6 Plot of the FPR under the third hypothesis. Figure S7 Plot of the MCC under the second hypothesis. Figure S8 Plot of the MCC under the third hypothesis. Figure S9 Plot of the sensitivity under the second hypothesis. Figure S10 Plot of the sensitivity under the third hypothesis. Figure S11 Plot of the ROC curve and the AUC value under the second hypothesis. Figure S12 Plot of the ROC under the third hypothesis. The DSLD2 method is developed in this paper. Figure S13 Precision-recall plot under the second hypothesis. Figure S14 Precision-recall plot under the third hypothesis. Figure S15 Bias plot of 6 meta-analysis methods when τ2 is set to 1.0 and SMD is chosen as the effect size measure. Figure S16 RMSE plot of 6 meta-analysis methods when τ2 is set to 1.0 and SMD is chosen as the effect size measure. Figure S17 Bias plot of 6 meta-analysis methods when τ2 is set to 1.0 and MD is chosen as the effect size measure. Figure S18 RMSE plot of 6 meta-analysis methods when τ2 is set to 1.0 and MD is chosen as the effect size measure. Figure S19 Mean of I2 plot of 6 meta-analysis methods when τ2 is set to 1.0 and SMD is chosen as the effect size measure. Figure S20 Mean of I2 plot of 6 meta-analysis methods when τ2 is set to 1.0 and MD is chosen as the effect size measure. [file 12864_2020_6500_MOESM3_ESM.zip › Figure S4.pdf]

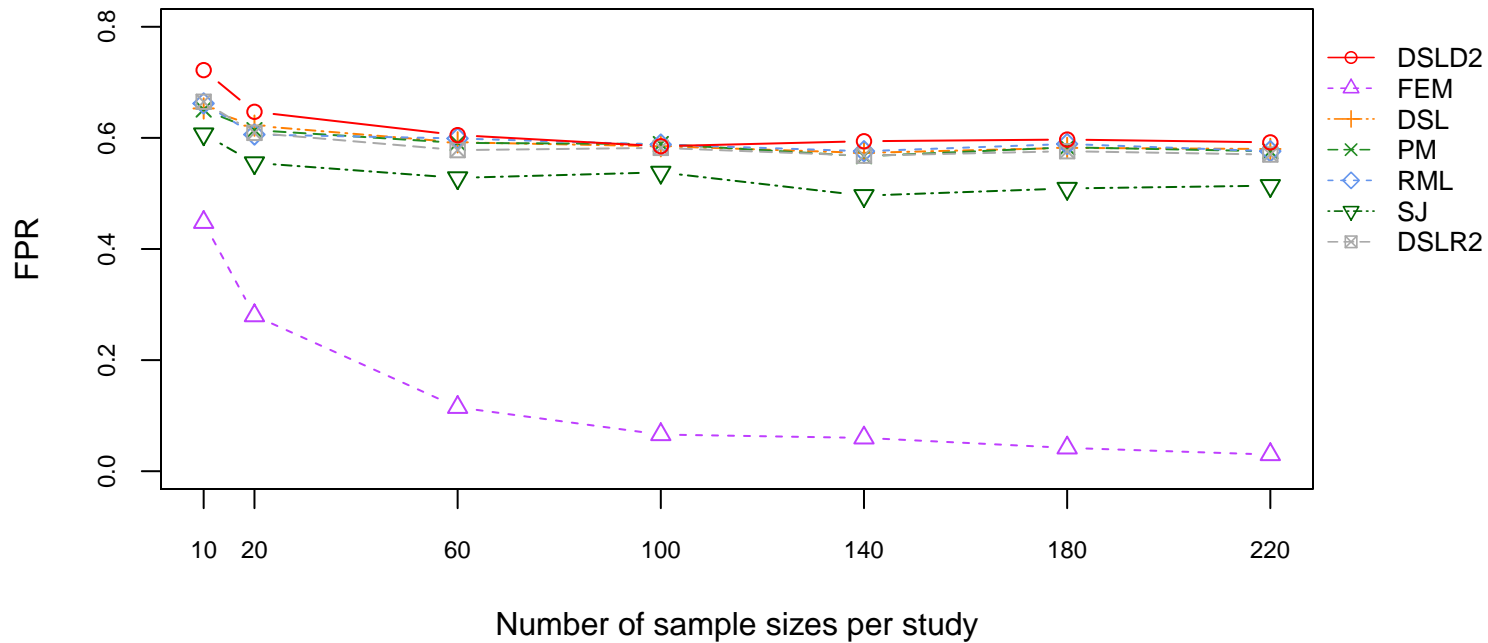

Supplement: Supplementary file 3 — Additional file 3 Supplementary figures. Figure S1 Plot of the precision under the second hypothesis. Figure S2 Plot of the precision under the third hypothesis. Figure S3 Plot of the accuracy under the second hypothesis. Figure S4 Plot of the accuracy under the third hypothesis. Figure S5 Plot of the FPR under the second hypothesis. Figure S6 Plot of the FPR under the third hypothesis. Figure S7 Plot of the MCC under the second hypothesis. Figure S8 Plot of the MCC under the third hypothesis. Figure S9 Plot of the sensitivity under the second hypothesis. Figure S10 Plot of the sensitivity under the third hypothesis. Figure S11 Plot of the ROC curve and the AUC value under the second hypothesis. Figure S12 Plot of the ROC under the third hypothesis. The DSLD2 method is developed in this paper. Figure S13 Precision-recall plot under the second hypothesis. Figure S14 Precision-recall plot under the third hypothesis. Figure S15 Bias plot of 6 meta-analysis methods when τ2 is set to 1.0 and SMD is chosen as the effect size measure. Figure S16 RMSE plot of 6 meta-analysis methods when τ2 is set to 1.0 and SMD is chosen as the effect size measure. Figure S17 Bias plot of 6 meta-analysis methods when τ2 is set to 1.0 and MD is chosen as the effect size measure. Figure S18 RMSE plot of 6 meta-analysis methods when τ2 is set to 1.0 and MD is chosen as the effect size measure. Figure S19 Mean of I2 plot of 6 meta-analysis methods when τ2 is set to 1.0 and SMD is chosen as the effect size measure. Figure S20 Mean of I2 plot of 6 meta-analysis methods when τ2 is set to 1.0 and MD is chosen as the effect size measure. [file 12864_2020_6500_MOESM3_ESM.zip › Figure S5.pdf]

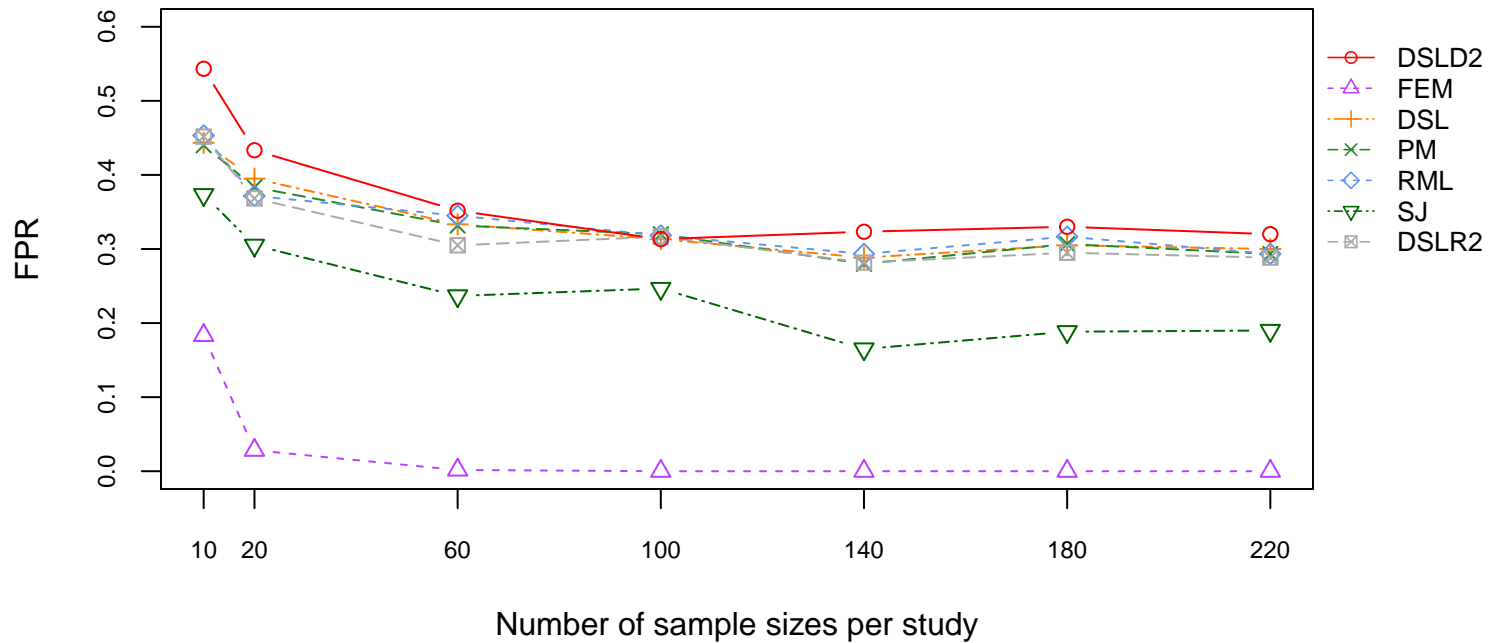

Supplement: Supplementary file 3 — Additional file 3 Supplementary figures. Figure S1 Plot of the precision under the second hypothesis. Figure S2 Plot of the precision under the third hypothesis. Figure S3 Plot of the accuracy under the second hypothesis. Figure S4 Plot of the accuracy under the third hypothesis. Figure S5 Plot of the FPR under the second hypothesis. Figure S6 Plot of the FPR under the third hypothesis. Figure S7 Plot of the MCC under the second hypothesis. Figure S8 Plot of the MCC under the third hypothesis. Figure S9 Plot of the sensitivity under the second hypothesis. Figure S10 Plot of the sensitivity under the third hypothesis. Figure S11 Plot of the ROC curve and the AUC value under the second hypothesis. Figure S12 Plot of the ROC under the third hypothesis. The DSLD2 method is developed in this paper. Figure S13 Precision-recall plot under the second hypothesis. Figure S14 Precision-recall plot under the third hypothesis. Figure S15 Bias plot of 6 meta-analysis methods when τ2 is set to 1.0 and SMD is chosen as the effect size measure. Figure S16 RMSE plot of 6 meta-analysis methods when τ2 is set to 1.0 and SMD is chosen as the effect size measure. Figure S17 Bias plot of 6 meta-analysis methods when τ2 is set to 1.0 and MD is chosen as the effect size measure. Figure S18 RMSE plot of 6 meta-analysis methods when τ2 is set to 1.0 and MD is chosen as the effect size measure. Figure S19 Mean of I2 plot of 6 meta-analysis methods when τ2 is set to 1.0 and SMD is chosen as the effect size measure. Figure S20 Mean of I2 plot of 6 meta-analysis methods when τ2 is set to 1.0 and MD is chosen as the effect size measure. [file 12864_2020_6500_MOESM3_ESM.zip › Figure S6.pdf]

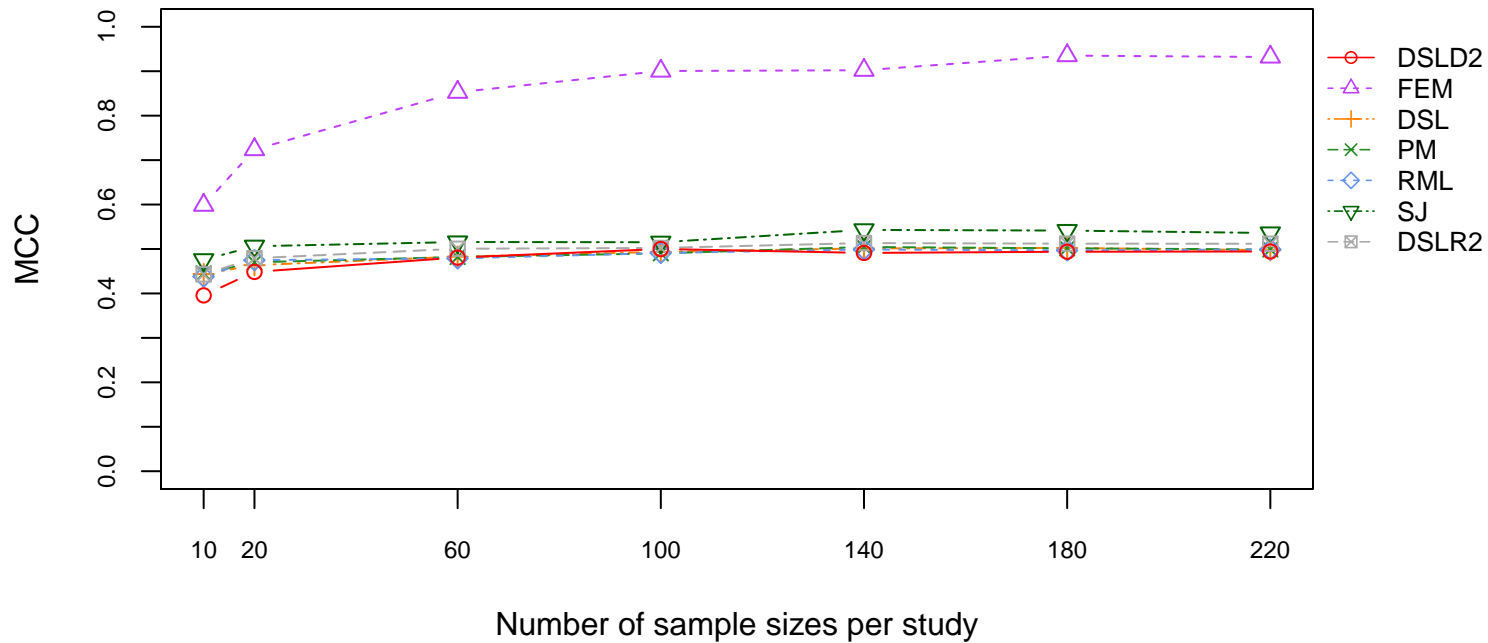

Supplement: Supplementary file 3 — Additional file 3 Supplementary figures. Figure S1 Plot of the precision under the second hypothesis. Figure S2 Plot of the precision under the third hypothesis. Figure S3 Plot of the accuracy under the second hypothesis. Figure S4 Plot of the accuracy under the third hypothesis. Figure S5 Plot of the FPR under the second hypothesis. Figure S6 Plot of the FPR under the third hypothesis. Figure S7 Plot of the MCC under the second hypothesis. Figure S8 Plot of the MCC under the third hypothesis. Figure S9 Plot of the sensitivity under the second hypothesis. Figure S10 Plot of the sensitivity under the third hypothesis. Figure S11 Plot of the ROC curve and the AUC value under the second hypothesis. Figure S12 Plot of the ROC under the third hypothesis. The DSLD2 method is developed in this paper. Figure S13 Precision-recall plot under the second hypothesis. Figure S14 Precision-recall plot under the third hypothesis. Figure S15 Bias plot of 6 meta-analysis methods when τ2 is set to 1.0 and SMD is chosen as the effect size measure. Figure S16 RMSE plot of 6 meta-analysis methods when τ2 is set to 1.0 and SMD is chosen as the effect size measure. Figure S17 Bias plot of 6 meta-analysis methods when τ2 is set to 1.0 and MD is chosen as the effect size measure. Figure S18 RMSE plot of 6 meta-analysis methods when τ2 is set to 1.0 and MD is chosen as the effect size measure. Figure S19 Mean of I2 plot of 6 meta-analysis methods when τ2 is set to 1.0 and SMD is chosen as the effect size measure. Figure S20 Mean of I2 plot of 6 meta-analysis methods when τ2 is set to 1.0 and MD is chosen as the effect size measure. [file 12864_2020_6500_MOESM3_ESM.zip › Figure S7.pdf]

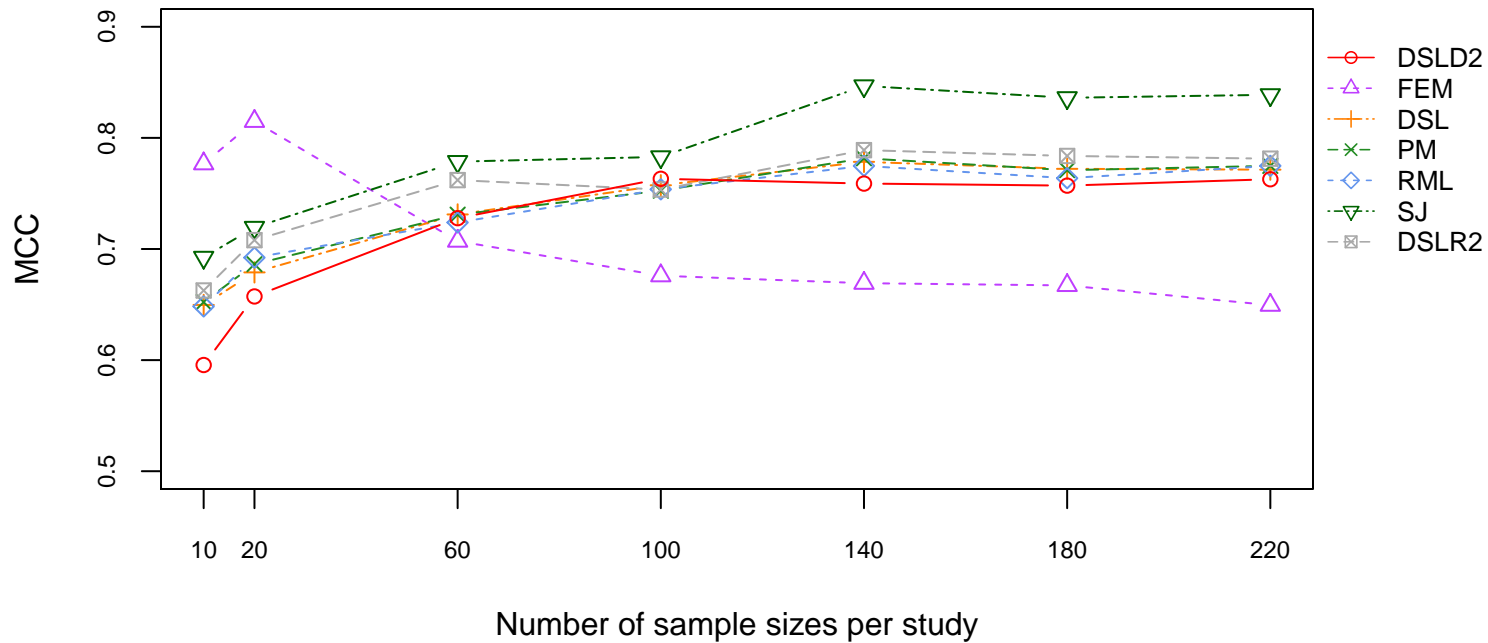

Supplement: Supplementary file 3 — Additional file 3 Supplementary figures. Figure S1 Plot of the precision under the second hypothesis. Figure S2 Plot of the precision under the third hypothesis. Figure S3 Plot of the accuracy under the second hypothesis. Figure S4 Plot of the accuracy under the third hypothesis. Figure S5 Plot of the FPR under the second hypothesis. Figure S6 Plot of the FPR under the third hypothesis. Figure S7 Plot of the MCC under the second hypothesis. Figure S8 Plot of the MCC under the third hypothesis. Figure S9 Plot of the sensitivity under the second hypothesis. Figure S10 Plot of the sensitivity under the third hypothesis. Figure S11 Plot of the ROC curve and the AUC value under the second hypothesis. Figure S12 Plot of the ROC under the third hypothesis. The DSLD2 method is developed in this paper. Figure S13 Precision-recall plot under the second hypothesis. Figure S14 Precision-recall plot under the third hypothesis. Figure S15 Bias plot of 6 meta-analysis methods when τ2 is set to 1.0 and SMD is chosen as the effect size measure. Figure S16 RMSE plot of 6 meta-analysis methods when τ2 is set to 1.0 and SMD is chosen as the effect size measure. Figure S17 Bias plot of 6 meta-analysis methods when τ2 is set to 1.0 and MD is chosen as the effect size measure. Figure S18 RMSE plot of 6 meta-analysis methods when τ2 is set to 1.0 and MD is chosen as the effect size measure. Figure S19 Mean of I2 plot of 6 meta-analysis methods when τ2 is set to 1.0 and SMD is chosen as the effect size measure. Figure S20 Mean of I2 plot of 6 meta-analysis methods when τ2 is set to 1.0 and MD is chosen as the effect size measure. [file 12864_2020_6500_MOESM3_ESM.zip › Figure S8.pdf]

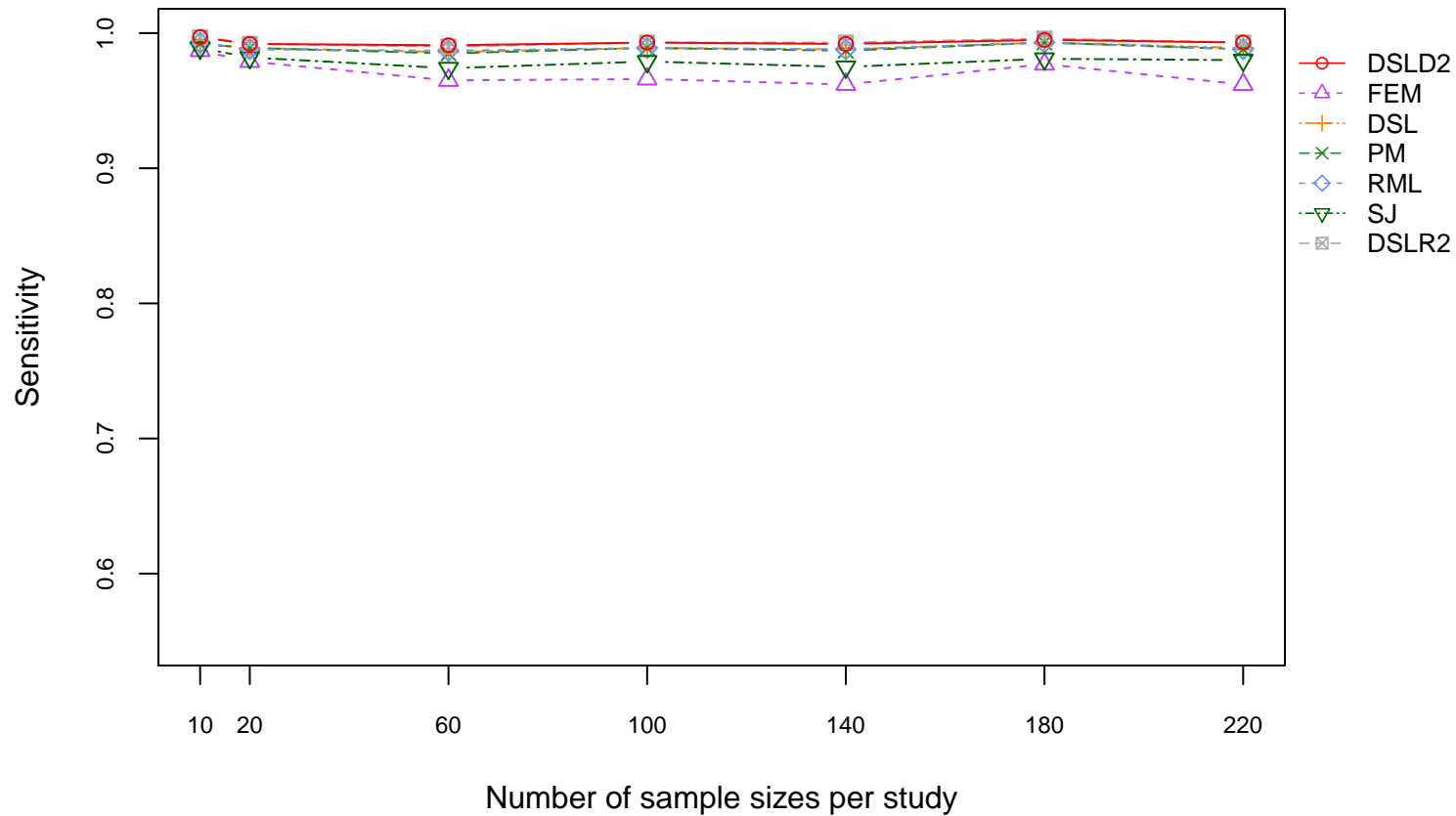

Supplement: Supplementary file 3 — Additional file 3 Supplementary figures. Figure S1 Plot of the precision under the second hypothesis. Figure S2 Plot of the precision under the third hypothesis. Figure S3 Plot of the accuracy under the second hypothesis. Figure S4 Plot of the accuracy under the third hypothesis. Figure S5 Plot of the FPR under the second hypothesis. Figure S6 Plot of the FPR under the third hypothesis. Figure S7 Plot of the MCC under the second hypothesis. Figure S8 Plot of the MCC under the third hypothesis. Figure S9 Plot of the sensitivity under the second hypothesis. Figure S10 Plot of the sensitivity under the third hypothesis. Figure S11 Plot of the ROC curve and the AUC value under the second hypothesis. Figure S12 Plot of the ROC under the third hypothesis. The DSLD2 method is developed in this paper. Figure S13 Precision-recall plot under the second hypothesis. Figure S14 Precision-recall plot under the third hypothesis. Figure S15 Bias plot of 6 meta-analysis methods when τ2 is set to 1.0 and SMD is chosen as the effect size measure. Figure S16 RMSE plot of 6 meta-analysis methods when τ2 is set to 1.0 and SMD is chosen as the effect size measure. Figure S17 Bias plot of 6 meta-analysis methods when τ2 is set to 1.0 and MD is chosen as the effect size measure. Figure S18 RMSE plot of 6 meta-analysis methods when τ2 is set to 1.0 and MD is chosen as the effect size measure. Figure S19 Mean of I2 plot of 6 meta-analysis methods when τ2 is set to 1.0 and SMD is chosen as the effect size measure. Figure S20 Mean of I2 plot of 6 meta-analysis methods when τ2 is set to 1.0 and MD is chosen as the effect size measure. [file 12864_2020_6500_MOESM3_ESM.zip › Figure S9.pdf]
