# Supplementary material for: A novel estimator of between-study variance in random-effects models
Source: BMC Genomics. 2020 Feb 11;21:149. doi: 10.1186/s12864-020-6500-9 (PMC7014785; doi:10.1186/s12864-020-6500-9)
Supplement: Supplementary file 4 — Additional file 4 Tables. Additional file 4 is the tables of the precision, accuracy, FPR, MCC and sensitivity under three hypothesis and tables of bias, RMSE and mean of I2. [file 12864_2020_6500_MOESM4_ESM.docx]

Additional file 4

Nan Wang^1†^, Jun Zhang^2†^, Li Xu^3†^, Jing Qi^1^, Beibei Liu^1^, Yiyang Tang^4^, Yinan Jiang^5^, Liang Cheng^6^, Qinghua Jiang^7^, Xunbo Yin^1^ and Shuilin Jin^1*^

1. Department of Mathematics, Harbin Institute of Technology, Harbin, Heilongjiang, China

2. College of Computer Science and Technology, Harbin Engineering University, Harbin, China

3. School of Life Science and Technology, Harbin Institute of Technology, Harbin, China

† Equally contributed to the work

* To whom all correspondence should be addressed

*Corresponding author:

Shuilin Jin

School of Mathematics, Harbin Institute of Technology, Harbin, Heilongjiang, China

E-mail:jinsl@hit.edu.cn

Table S1. The precision under the first hypothesis of 7 meta-analysis methods

| Method\Samples | 10 | 20 | 60 | 100 | 140 | 180 | 220 |
| --- | --- | --- | --- | --- | --- | --- | --- |
| DSLD2 | 0.977 | 0.992 | 1 | 1 | 1 | 1 | 1 |
| DSLR2 | 0.981 | 0.992 | 0.998 | 0.998 | 0.997 | 0.994 | 0.998 |
| FEM | 0.995 | 1 | 1 | 1 | 1 | 1 | 1 |
| DSL | 0.983 | 0.991 | 0.999 | 0.999 | 1 | 1 | 1 |
| PM | 0.983 | 0.991 | 0.999 | 0.999 | 1 | 1 | 1 |
| RML | 0.981 | 0.992 | 0.999 | 0.999 | 1 | 1 | 1 |
| SJ | 0.987 | 0.992 | 1 | 0.999 | 1 | 1 | 1 |

Note: DSLD2 represents the two-step estimation starting with the DSL estimate and the $D^{2}$ in the second step that this paper developed; DSLR2 is the two-step estimation starting with the DSL estimate and the $R^{2}$ in the second step; DSL denotes the standard random-effects model; FEM is the fixed-effects model.

Table S2. The precision under the second hypothesis of 7 meta-analysis methods.

| Method\Samples | 10 | 20 | 60 | 100 | 140 | 180 | 220 |
| --- | --- | --- | --- | --- | --- | --- | --- |
| DSLD2 | 0.579 | 0.605 | 0.620 | 0.629 | 0.625 | 0.625 | 0.62 |
| DSLR2 | 0.599 | 0.619 | 0.631 | 0.630 | 0.636 | 0.633 | 0.635 |
| FEM | 0.687 | 0.777 | 0.893 | 0.936 | 0.941 | 0.958 | 0.969 |
| DSL | 0.603 | 0.613 | 0.624 | 0.628 | 0.632 | 0.630 | 0.630 |
| PM | 0.603 | 0.616 | 0.625 | 0.626 | 0.635 | 0.630 | 0.631 |
| RML | 0.6 | 0.619 | 0.622 | 0.627 | 0.631 | 0.627 | 0.631 |
| SJ | 0.619 | 0.638 | 0.648 | 0.645 | 0.662 | 0.658 | 0.655 |

Note: DSLD2 represents the two-step estimation starting with the DSL estimate and the $D^{2}$ in the second step that this paper developed; DSLR2 is the two-step estimation starting with the DSL estimate and the $R^{2}$ in the second step; DSL denotes the standard random-effects model; FEM is the fixed-effects model.

Table S3. The precision under the third hypothesis of 7 meta-analysis methods.

| Method\Samples | 10 | 20 | 60 | 100 | 140 | 180 | 220 |
| --- | --- | --- | --- | --- | --- | --- | --- |
| DSLD2 | 0.810 | 0.841 | 0.867 | 0.880 | 0.877 | 0.875 | 0.878 |
| DSLR2 | 0.836 | 0.861 | 0.883 | 0.879 | 0.891 | 0.887 | 0.889 |
| FEM | 0.923 | 0.986 | 0.999 | 1 | 1 | 1 | 1 |
| DSL | 0.838 | 0.852 | 0.873 | 0.880 | 0.889 | 0.883 | 0.885 |
| PM | 0.839 | 0.856 | 0.873 | 0.878 | 0.891 | 0.883 | 0.887 |
| RML | 0.835 | 0.860 | 0.869 | 0.878 | 0.887 | 0.879 | 0.887 |
| SJ | 0.859 | 0.880 | 0.905 | 0.902 | 0.932 | 0.924 | 0.923 |

Note: DSLD2 represents the two-step estimation starting with the DSL estimate and the $D^{2}$ in the second step that this paper developed; DSLR2 is the two-step estimation starting with the DSL estimate and the $R^{2}$ in the second step; DSL denotes the standard random-effects model; FEM is the fixed-effects model.

Table S4: The accuracy under the first hypothesis of 7 meta-analysis methods.

| Method\Samples | 10 | 20 | 60 | 100 | 140 | 180 | 220 |
| --- | --- | --- | --- | --- | --- | --- | --- |
| DSLD2 | 0.9205 | 0.9065 | 0.898 | 0.889 | 0.893 | 0.896 | 0.8925 |
| DSLR2 | 0.9 | 0.8885 | 0.881 | 0.8845 | 0.8765 | 0.877 | 0.8785 |
| FEM | 0.8105 | 0.7295 | 0.64 | 0.616 | 0.611 | 0.6095 | 0.596 |
| DSL | 0.8955 | 0.8925 | 0.888 | 0.886 | 0.8805 | 0.8875 | 0.8845 |
| PM | 0.8945 | 0.8885 | 0.887 | 0.888 | 0.877 | 0.888 | 0.882 |
| RML | 0.8975 | 0.885 | 0.892 | 0.8875 | 0.882 | 0.891 | 0.882 |
| SJ | 0.878 | 0.8575 | 0.851 | 0.8575 | 0.8355 | 0.845 | 0.847 |

Note: DSLD2 represents the two-step estimation starting with the DSL estimate and the $D^{2}$ in the second step that this paper developed; DSLR2 is the two-step estimation starting with the DSL estimate and the $R^{2}$ in the second step; DSL denotes the standard random-effects model; FEM is the fixed-effects model.

Table S5. The accuracy under the second hypothesis of 7 meta-analysis methods.

| Method\Samples | 10 | 20 | 60 | 100 | 140 | 180 | 220 |
| --- | --- | --- | --- | --- | --- | --- | --- |
| DSLD2 | 0.6375 | 0.6725 | 0.693 | 0.704 | 0.699 | 0.699 | 0.7005 |
| DSLR2 | 0.666 | 0.6915 | 0.706 | 0.7055 | 0.7125 | 0.71 | 0.7115 |
| FEM | 0.7695 | 0.8495 | 0.925 | 0.95 | 0.951 | 0.9675 | 0.966 |
| DSL | 0.6695 | 0.6835 | 0.697 | 0.702 | 0.7075 | 0.7055 | 0.7045 |
| PM | 0.6705 | 0.6875 | 0.697 | 0.7 | 0.71 | 0.705 | 0.706 |
| RML | 0.6655 | 0.691 | 0.694 | 0.7005 | 0.706 | 0.702 | 0.706 |
| SJ | 0.691 | 0.7135 | 0.723 | 0.7205 | 0.7395 | 0.736 | 0.733 |

Note: DSLD2 represents the two-step estimation starting with the DSL estimate and the $D^{2}$ in the second step that this paper developed; DSLR2 is the two-step estimation starting with the DSL estimate and the $R^{2}$ in the second step; DSL denotes the standard random-effects model; FEM was the fixed-effects model.

Table S6. The accuracy under the third hypothesis of 7 meta-analysis methods.

| Method\Samples | 10 | 20 | 60 | 100 | 140 | 180 | 220 |
| --- | --- | --- | --- | --- | --- | --- | --- |
| DSLD2 | 0.8335 | 0.8595 | 0.887 | 0.901 | 0.899 | 0.898 | 0.9005 |
| DSLR2 | 0.86 | 0.8795 | 0.901 | 0.8975 | 0.9115 | 0.909 | 0.9085 |
| FEM | 0.9075 | 0.9125 | 0.839 | 0.816 | 0.811 | 0.8095 | 0.796 |
| DSL | 0.8565 | 0.8685 | 0.889 | 0.899 | 0.9075 | 0.9045 | 0.9045 |
| PM | 0.8575 | 0.8715 | 0.889 | 0.897 | 0.909 | 0.904 | 0.906 |
| RML | 0.8555 | 0.874 | 0.886 | 0.8975 | 0.906 | 0.901 | 0.906 |
| SJ | 0.874 | 0.8855 | 0.909 | 0.9105 | 0.9365 | 0.932 | 0.933 |

Note: DSLD2 represents the two-step estimation starting with the DSL estimate and the $D^{2}$ in the second step that this paper developed; DSLR2 is the two-step estimation starting with the DSL estimate and the $R^{2}$ in the second step; DSL denotes the standard random-effects model; FEM was the fixed-effects model.

Table S7: The FPR under the first hypothesis of 7 meta-analysis methods.

| Method\Samples | 10 | 20 | 60 | 100 | 140 | 180 | 220 |
| --- | --- | --- | --- | --- | --- | --- | --- |
| DSLD2 | 0.195 | 0.065 | 0 | 0 | 0 | 0 | 0 |
| DSLR2 | 0.155 | 0.06 | 0.015 | 0.015 | 0.02 | 0.045 | 0.015 |
| FEM | 0.035 | 0 | 0 | 0 | 0 | 0 | 0 |
| DSL | 0.135 | 0.065 | 0.005 | 0.005 | 0 | 0 | 0 |
| PM | 0.135 | 0.065 | 0.005 | 0.005 | 0 | 0 | 0 |
| RML | 0.15 | 0.06 | 0.005 | 0.005 | 0 | 0 | 0 |
| SJ | 0.1 | 0.055 | 0 | 0.005 | 0 | 0 | 0 |

Note: DSLD2 represents the two-step estimation starting with the DSL estimate and the $D^{2}$ in the second step that this paper developed; DSLR2 is the two-step estimation starting with the DSL estimate and the $R^{2}$ in the second step; DSL denotes the standard random-effects model; FEM was the fixed-effects model.

Table S8. The FPR under the second hypothesis of 7 meta-analysis methods.

| Method\Samples | 10 | 20 | 60 | 100 | 140 | 180 | 220 |
| --- | --- | --- | --- | --- | --- | --- | --- |
| DSLD2 | 0.722 | 0.647 | 0.605 | 0.585 | 0.594 | 0.597 | 0.592 |
| DSLR2 | 0.665 | 0.609 | 0.578 | 0.582 | 0.568 | 0.576 | 0.57 |
| FEM | 0.448 | 0.28 | 0.115 | 0.066 | 0.06 | 0.042 | 0.03 |
| DSL | 0.653 | 0.622 | 0.592 | 0.585 | 0.573 | 0.582 | 0.58 |
| PM | 0.651 | 0.614 | 0.591 | 0.589 | 0.567 | 0.583 | 0.576 |
| RML | 0.662 | 0.606 | 0.599 | 0.588 | 0.576 | 0.589 | 0.576 |
| SJ | 0.607 | 0.555 | 0.528 | 0.538 | 0.496 | 0.509 | 0.514 |

Note: DSLD2 represents the two-step estimation starting with the DSL estimate and the $D^{2}$ in the second step that this paper developed; DSLR2 is the two-step estimation starting with the DSL estimate and the $R^{2}$ in the second step; DSL denotes the standard random-effects model; FEM was the fixed-effects model.

Table S9. The FPR under the third hypothesis of 7 meta-analysis methods.

| Method\Samples | 10 | 20 | 60 | 100 | 140 | 180 | 220 |
| --- | --- | --- | --- | --- | --- | --- | --- |
| DSLD2 | 0.543 | 0.433 | 0.351 | 0.313 | 0.323 | 0.33 | 0.32 |
| DSLR2 | 0.451 | 0.368 | 0.305 | 0.316 | 0.281 | 0.295 | 0.288 |
| FEM | 0.183 | 0.028 | 0.001 | 0 | 0 | 0 | 0 |
| DSL | 0.443 | 0.395 | 0.333 | 0.313 | 0.288 | 0.305 | 0.3 |
| PM | 0.44 | 0.383 | 0.331 | 0.32 | 0.28 | 0.306 | 0.293 |
| RML | 0.453 | 0.371 | 0.345 | 0.318 | 0.293 | 0.316 | 0.293 |
| SJ | 0.373 | 0.305 | 0.236 | 0.246 | 0.165 | 0.188 | 0.19 |

Note: DSLD2 represents the two-step estimation starting with the DSL estimate and the $D^{2}$ in the second step that this paper developed; DSLR2 is the two-step estimation starting with the DSL estimate and the $R^{2}$ in the second step; DSL denotes the standard random-effects model; FEM was the fixed-effects model.

Table S10. The MCC under the first hypothesis of 7 meta-analysis methods.

| Method\Samples | 10 | 20 | 60 | 100 | 140 | 180 | 220 |
| --- | --- | --- | --- | --- | --- | --- | --- |
| DSLD2 | 0.637 | 0.653 | 0.662 | 0.644 | 0.652 | 0.658 | 0.651 |
| DSLR2 | 0.601 | 0.617 | 0.622 | 0.629 | 0.612 | 0.602 | 0.618 |
| FEM | 0.505 | 0.434 | 0.361 | 0.344 | 0.340 | 0.339 | 0.330 |
| DSL | 0.599 | 0.623 | 0.640 | 0.636 | 0.628 | 0.641 | 0.635 |
| PM | 0.597 | 0.6154 | 0.638 | 0.640 | 0.622 | 0.642 | 0.631 |
| RML | 0.597 | 0.610 | 0.648 | 0.639 | 0.631 | 0.648 | 0.631 |
| SJ | 0.579 | 0.563 | 0.578 | 0.586 | 0.555 | 0.569 | 0.572 |

Note: DSLD2 represents the two-step estimation starting with the DSL estimate and the $D^{2}$ in the second step that this paper developed; DSLR2 is the two-step estimation starting with the DSL estimate and the $R^{2}$ in the second step; DSL denotes the standard random-effects model; FEM was the fixed-effects model.

Table S11. The MCC under the second hypothesis of 7 meta-analysis methods.

| Method\Samples | 10 | 20 | 60 | 100 | 140 | 180 | 220 |
| --- | --- | --- | --- | --- | --- | --- | --- |
| DSLD2 | 0.395 | 0.448 | 0.480 | 0.499 | 0.491 | 0.493 | 0.494 |
| DSLR2 | 0.442 | 0.479 | 0.500 | 0.502 | 0.513 | 0.512 | 0.511 |
| FEM | 0.598 | 0.723 | 0.852 | 0.900 | 0.902 | 0.935 | 0.932 |
| DSL | 0.443 | 0.463 | 0.482 | 0.493 | 0.501 | 0.502 | 0.497 |
| PM | 0.445 | 0.470 | 0.481 | 0.490 | 0.504 | 0.501 | 0.498 |
| RML | 0.438 | 0.474 | 0.478 | 0.490 | 0.498 | 0.496 | 0.498 |
| SJ | 0.475 | 0.506 | 0.515 | 0.515 | 0.543 | 0.541 | 0.535 |

Note: DSLD2 represents the two-step estimation starting with the DSL estimate and the $D^{2}$ in the second step that this paper developed; DSLR2 is the two-step estimation starting with the DSL estimate and the $R^{2}$ in the second step; DSL denotes the standard random-effects model; FEM was the fixed-effects model.

Table S12. The MCC under the third hypothesis of 7 meta-analysis methods.

| Method\Samples | 10 | 20 | 60 | 100 | 140 | 180 | 220 |
| --- | --- | --- | --- | --- | --- | --- | --- |
| DSLD2 | 0.595 | 0.657 | 0.727 | 0.763 | 0.758 | 0.757 | 0.762 |
| DSLR2 | 0.662 | 0.707 | 0.762 | 0.753 | 0.788 | 0.783 | 0.781 |
| FEM | 0.776 | 0.814 | 0.707 | 0.675 | 0.669 | 0.667 | 0.649 |
| DSL | 0.649 | 0.678 | 0.731 | 0.757 | 0.778 | 0.772 | 0.771 |
| PM | 0.652 | 0.686 | 0.730 | 0.752 | 0.781 | 0.770 | 0.774 |
| RML | 0.648 | 0.692 | 0.723 | 0.753 | 0.774 | 0.763 | 0.774 |
| SJ | 0.692 | 0.719 | 0.778 | 0.782 | 0.846 | 0.836 | 0.838 |

Note: DSLD2 represents the two-step estimation starting with the DSL estimate and the $D^{2}$ in the second step that this paper developed; DSLR2 is the two-step estimation starting with the DSL estimate and the $R^{2}$ in the second step; DSL denotes the standard random-effects model; FEM was the fixed-effects model.

Table S13. The sensitivity under the first hypothesis of 7 meta-analysis methods.

| Method\Samples | 10 | 20 | 60 | 100 | 140 | 180 | 220 |
| --- | --- | --- | --- | --- | --- | --- | --- |
| DSLD2 | 0.933 | 0.903 | 0.886 | 0.876 | 0.881 | 0.884 | 0.880 |
| DSLR2 | 0.906 | 0.882 | 0.869 | 0.873 | 0.865 | 0.868 | 0.866 |
| FEM | 0.793 | 0.699 | 0.6 | 0.573 | 0.567 | 0.566 | 0.551 |
| DSL | 0.898 | 0.887 | 0.876 | 0.873 | 0.867 | 0.875 | 0.871 |
| PM | 0.897 | 0.883 | 0.875 | 0.876 | 0.863 | 0.875 | 0.868 |
| RML | 0.902 | 0.878 | 0.880 | 0.875 | 0.868 | 0.878 | 0.868 |
| SJ | 0.875 | 0.847 | 0.834 | 0.842 | 0.817 | 0.827 | 0.83 |

Note: DSLD2 represents the two-step estimation starting with the DSL estimate and the $D^{2}$ in the second step that this paper developed; DSLR2 is the two-step estimation starting with the DSL estimate and the $R^{2}$ in the second step; DSL denotes the standard random-effects model; FEM was the fixed-effects model.

Table S14. The sensitivity under the second hypothesis of 7 meta-analysis methods.

| Method\Samples | 10 | 20 | 60 | 100 | 140 | 180 | 220 |
| --- | --- | --- | --- | --- | --- | --- | --- |
| DSLD2 | 0.997 | 0.992 | 0.991 | 0.993 | 0.992 | 0.995 | 0.993 |
| DSLR2 | 0.997 | 0.992 | 0.99 | 0.993 | 0.993 | 0.996 | 0.993 |
| FEM | 0.987 | 0.979 | 0.965 | 0.966 | 0.962 | 0.977 | 0.962 |
| DSL | 0.992 | 0.989 | 0.986 | 0.989 | 0.988 | 0.993 | 0.989 |
| PM | 0.992 | 0.989 | 0.985 | 0.989 | 0.987 | 0.993 | 0.988 |
| RML | 0.993 | 0.988 | 0.987 | 0.989 | 0.988 | 0.993 | 0.988 |
| SJ | 0.989 | 0.982 | 0.974 | 0.979 | 0.975 | 0.981 | 0.98 |

Note: DSLD2 represents the two-step estimation starting with the DSL estimate and the $D^{2}$ in the second step that this paper developed; DSLR2 is the two-step estimation starting with the DSL estimate and the $R^{2}$ in the second step; DSL denotes the standard random-effects model; FEM was the fixed-effects model.

Table S15. The sensitivity under the third hypothesis of 7 meta-analysis methods.

| Method\Samples | 10 | 20 | 60 | 100 | 140 | 180 | 220 |
| --- | --- | --- | --- | --- | --- | --- | --- |
| DSLD2 | 0.995 | 0.985 | 0.989 | 0.992 | 0.994 | 0.995 | 0.995 |
| DSLR2 | 0.993 | 0.985 | 0.989 | 0.989 | 0.994 | 0.996 | 0.992 |
| FEM | 0.946 | 0.887 | 0.770 | 0.737 | 0.73 | 0.727 | 0.708 |
| DSL | 0.985 | 0.981 | 0.9842 | 0.99 | 0.991 | 0.994 | 0.992 |
| PM | 0.985 | 0.980 | 0.983 | 0.99 | 0.99 | 0.994 | 0.991 |
| RML | 0.987 | 0.979 | 0.985 | 0.99 | 0.991 | 0.994 | 0.991 |
| SJ | 0.98 | 0.967 | 0.971 | 0.977 | 0.98 | 0.983 | 0.985 |

Note: DSLD2 represents the two-step estimation starting with the DSL estimate and the $D^{2}$ in the second step that this paper developed; DSLR2 is the two-step estimation starting with the DSL estimate and the $R^{2}$ in the second step; DSL denotes the standard random-effects model; FEM was the fixed-effects model.

Table S16. The bias of 6 methods when $\tau^{2}$ is set to 0.0 and SMD is chosen as the eﬀect size measure

| Method\Studies | 5 | 10 | 20 | 40 | 80 |
| --- | --- | --- | --- | --- | --- |
| DSLD2 | 0.01357 | 0.00827 | 0.00583 | 0.00391 | 0.00251 |
| DSLR2 | 0.12392 | 0.10075 | 0.08138 | 0.05844 | 0.04210 |
| DSL | 0.01416 | 0.00846 | 0.00610 | 0.00401 | 0.00254 |
| PM | 0.01644 | 0.00893 | 0.00667 | 0.00419 | 0.00259 |
| RML | 0.01381 | 0.00818 | 0.00553 | 0.00369 | 0.00248 |
| SJ | 0.03364 | 0.02645 | 0.03175 | 0.02949 | 0.02842 |

Note: DSLD2 represents the two-step estimation starting with the DSL estimate and the $D^{2}$ in the second step that this paper developed; DSLR2 is the two-step estimation starting with the DSL estimate and the $R^{2}$ in the second step; DSL denotes the standard random-effects model.

Table S17. The RMSE of 6 methods when $\tau^{2}$ is set to 0.0 and SMD is chosen as the eﬀect size measure

| Method\Studies | 5 | 10 | 20 | 40 | 80 |
| --- | --- | --- | --- | --- | --- |
| DSLD2 | 0.02883 | 0.01753 | 0.01145 | 0.00769 | 0.00495 |
| DSLR2 | 0.22736 | 0.18525 | 0.14594 | 0.10704 | 0.07942 |
| DSL | 0.03001 | 0.01792 | 0.01198 | 0.00792 | 0.00502 |
| PM | 0.03778 | 0.01922 | 0.01356 | 0.00836 | 0.00515 |
| RML | 0.03053 | 0.01780 | 0.01130 | 0.00751 | 0.00487 |
| SJ | 0.05043 | 0.03295 | 0.03703 | 0.03131 | 0.02947 |

Note: DSLD2 represents the two-step estimation starting with the DSL estimate and the $D^{2}$ in the second step that this paper developed; DSLR2 is the two-step estimation starting with the DSL estimate and the $R^{2}$ in the second step; DSL denotes the standard random-effects model.

Table S18. The bias of 6 methods when $\tau^{2}$ is set to 0.0 and MD is chosen as the eﬀect size measure

| Method\Studies | 5 | 10 | 20 | 40 | 80 |
| --- | --- | --- | --- | --- | --- |
| DSLD2 | 1.51416 | 0.93916 | 0.75783 | 0.54485 | 0.73019 |
| DSLR2 | 0.13960 | 0.11562 | 0.10464 | 0.08222 | 0.09223 |
| DSL | 1.56460 | 0.94450 | 0.78659 | 0.54611 | 0.70341 |
| PM | 1.76337 | 0.96821 | 0.85867 | 0.55234 | 0.56675 |
| RML | 1.56872 | 0.95043 | 0.74078 | 0.55792 | 0.585258 |
| SJ | 3.41444 | 2.67228 | 3.25929 | 2.98763 | 3.077457 |

Note: DSLD2 represents the two-step estimation starting with the DSL estimate and the $D^{2}$ in the second step that this paper developed; DSLR2 is the two-step estimation starting with the DSL estimate and the $R^{2}$ in the second step; DSL denotes the standard random-effects model.

Table S19. The RMSE of 6 methods when $\tau^{2}$ is set to 0.0 and MD is chosen as the eﬀect size measure

| Method\Studies | 5 | 10 | 20 | 40 | 80 |
| --- | --- | --- | --- | --- | --- |
| DSLD2 | 3.13503 | 1.91765 | 1.39230 | 0.96112 | 2.64841 |
| DSLR2 | 0.24702 | 0.20476 | 0.17454 | 0.13496 | 0.16027 |
| DSL | 3.24225 | 1.92810 | 1.44828 | 0.96703 | 2.42802 |
| PM | 3.89410 | 2.00369 | 1.66868 | 0.99133 | 1.05756 |
| RML | 3.36972 | 1.93166 | 1.38300 | 0.97625 | 1.12261 |
| SJ | 5.04097 | 3.31415 | 3.77961 | 3.16523 | 3.22527 |

Note: DSLD2 represents the two-step estimation starting with the DSL estimate and the $D^{2}$ in the second step that this paper developed; DSLR2 is the two-step estimation starting with the DSL estimate and the $R^{2}$ in the second step; DSL denotes the standard random-effects model.

Table S20. The bias of 6 methods when $\tau^{2}$ is set to 1.0 and SMD is chosen as the eﬀect size measure

| Method\Studies | 5 | 10 | 20 | 40 | 80 |
| --- | --- | --- | --- | --- | --- |
| DSLD2 | -0.97827 | -0.98300 | -0.98818 | -0.98949 | -0.99022 |
| DSLR2 | -0.82186 | -0.84791 | -0.84238 | -0.84775 | -0.85611 |
| DSL | -0.97803 | -0.98269 | -0.98794 | -0.98931 | -0.99006 |
| PM | -0.97718 | -0.98153 | -0.98729 | -0.98902 | -0.98980 |
| RML | -0.97791 | -0.98279 | -0.98823 | -0.98975 | -0.99054 |
| SJ | -0.96360 | -0.95408 | -0.96475 | -0.96654 | -0.96349 |

Note: DSLD2 represents the two-step estimation starting with the DSL estimate and the $D^{2}$ in the second step that this paper developed; DSLR2 is the two-step estimation starting with the DSL estimate and the $R^{2}$ in the second step; DSL denotes the standard random-effects model.

Table S21. The RMSE of 6 methods when $\tau^{2}$ is set to 1.0 and SMD is chosen as the eﬀect size measure

| Method\Studies | 5 | 10 | 20 | 40 | 80 |
| --- | --- | --- | --- | --- | --- |
| DSLD2 | 0.97896 | 0.98331 | 0.98829 | 0.98954 | 0.99026 |
| DSLR2 | 0.85386 | 0.86760 | 0.85834 | 0.85812 | 0.86302 |
| DSL | 0.97874 | 0.98302 | 0.98806 | 0.98937 | 0.99010 |
| PM | 0.97800 | 0.98193 | 0.98743 | 0.98909 | 0.98984 |
| RML | 0.97868 | 0.98312 | 0.98834 | 0.98981 | 0.99058 |
| SJ | 0.96441 | 0.95469 | 0.96492 | 0.96660 | 0.96353 |

Note: DSLD2 represents the two-step estimation starting with the DSL estimate and the $D^{2}$ in the second step that this paper developed; DSLR2 is the two-step estimation starting with the DSL estimate and the $R^{2}$ in the second step; DSL denotes the standard random-effects model.

Table S22. The bias of 6 methods when $\tau^{2}$ is set to 1.0 and MD is chosen as the eﬀect size measure

| Method\Studies | 5 | 10 | 20 | 40 | 80 |
| --- | --- | --- | --- | --- | --- |
| DSLD2 | 1.30590 | 1.02903 | 0.37061 | 0.25805 | 0.24167 |
| DSLR2 | -0.80860 | -0.81786 | -0.81594 | -0.81717 | -0.81722 |
| DSL | 1.30959 | 1.03792 | 0.37533 | 0.24985 | 0.23471 |
| PM | 1.34145 | 1.10711 | 0.40614 | 0.24306 | 0.22944 |
| RML | 1.31896 | 1.04866 | 0.39125 | 0.28691 | 0.27225 |
| SJ | 2.65551 | 3.64716 | 2.54630 | 2.37535 | 2.69533 |

Note: DSLD2 represents the two-step estimation starting with the DSL estimate and the $D^{2}$ in the second step that this paper developed; DSLR2 is the two-step estimation starting with the DSL estimate and the $R^{2}$ in the second step; DSL denotes the standard random-effects model.

Table S23. The RMSE of 6 methods when $\tau^{2}$ is set to 1.0 and MD is chosen as the eﬀect size measure

| Method\Studies | 5 | 10 | 20 | 40 | 80 |
| --- | --- | --- | --- | --- | --- |
| DSLD**2** | 4.04836 | 2.95230 | 1.64700 | 1.19915 | 0.95848 |
| DSLR2 | 0.84401 | 0.84210 | 0.83481 | 0.82966 | 0.82554 |
| DSL | 4.05035 | 2.95725 | 1.66109 | 1.19395 | 0.95524 |
| PM | 4.16663 | 3.17824 | 1.76087 | 1.19967 | 0.96117 |
| RML | 4.10717 | 2.96613 | 1.68572 | 1.19747 | 0.95006 |
| SJ | 4.69424 | 4.87483 | 3.08290 | 2.62029 | 2.83441 |

Note: DSLD2 represents the two-step estimation starting with the DSL estimate and the $D^{2}$ in the second step that this paper developed; DSLR2 is the two-step estimation starting with the DSL estimate and the $R^{2}$ in the second step; DSL denotes the standard random-effects model.

Table S24. Mean of $I^{2}$ statistic of 6 methods when $\tau^{2}$ is set to 0.0 and SMD is chosen as the eﬀect size measure

| Method\Studies | 5 | 10 | 20 | 40 | 80 |
| --- | --- | --- | --- | --- | --- |
| DSLD2 | 0.08747 | 0.07556 | 0.06409 | 0.04865 | 0.03643 |
| DSLR2 | 0.17089 | 0.17250 | 0.17381 | 0.16340 | 0.14517 |
| DSL | 0.08922 | 0.07652 | 0.06586 | 0.04948 | 0.03673 |
| PM | 0.09201 | 0.07799 | 0.06851 | 0.05065 | 0.03713 |
| RML | 0.08644 | 0.07383 | 0.06079 | 0.04577 | 0.03616 |
| SJ | 0.20813 | 0.22444 | 0.25160 | 0.25107 | 0.25614 |

Note: DSLD2 represents the two-step estimation starting with the DSL estimate and the $D^{2}$ in the second step that this paper developed; DSLR2 is the two-step estimation starting with the DSL estimate and the $R^{2}$ in the second step; DSL denotes the standard random-effects model.

Table S25. Mean of $I^{2}$ statistic of 6 methods when $\tau^{2}$ is set to 0.0 and MD is chosen as the eﬀect size measure

| Method\Studies | 5 | 10 | 20 | 40 | 80 |
| --- | --- | --- | --- | --- | --- |
| DSLD2 | 0.09695 | 0.08476 | 0.08038 | 0.06658 | 0.07243 |
| DSLR2 | 0.02200 | 0.02072 | 0.01902 | 0.01488 | 0.01719 |
| DSL | 0.09828 | 0.08501 | 0.08199 | 0.06660 | 0.07217 |
| PM | 0.10042 | 0.08544 | 0.08452 | 0.06676 | 0.07090 |
| RML | 0.09643 | 0.08614 | 0.07897 | 0.06798 | 0.07285 |
| SJ | 0.21338 | 0.22844 | 0.25821 | 0.25616 | 0.26811 |

Note: DSLD2 represents the two-step estimation starting with the DSL estimate and the $D^{2}$ in the second step that this paper developed; DSLR2 is the two-step estimation starting with the DSL estimate and the $R^{2}$ in the second step; DSL denotes the standard random-effects model.

Table S26. Mean of $I^{2}$ statistic of 6 methods when $\tau^{2}$ is set to 1.0 and SMD is chosen as the eﬀect size measure

| Method\Studies | 5 | 10 | 20 | 40 | 80 |
| --- | --- | --- | --- | --- | --- |
| DSLD2 | 0.12171 | 0.10970 | 0.11712 | 0.12008 | 0.11650 |
| DSLR2 | 0.21254 | 0.22638 | 0.27363 | 0.31389 | 0.33546 |
| DSL | 0.12233 | 0.11076 | 0.11824 | 0.12119 | 0.11765 |
| PM | 0.12326 | 0.11265 | 0.12011 | 0.12277 | 0.11926 |
| RML | 0.12104 | 0.11013 | 0.11508 | 0.11703 | 0.11326 |
| SJ | 0.22136 | 0.25303 | 0.27396 | 0.27791 | 0.28323 |

Note: DSLD2 represents the two-step estimation starting with the DSL estimate and the $D^{2}$ in the second step that this paper developed; DSLR2 is the two-step estimation starting with the DSL estimate and the $R^{2}$ in the second step; DSL denotes the standard random-effects model.

Table S27. Mean of $I^{2}$ statistic of 6 methods when $\tau^{2}$ is set to 1.0 and MD is chosen as the eﬀect size measure

| Method\Studies | 5 | 10 | 20 | 40 | 80 |
| --- | --- | --- | --- | --- | --- |
| DSLD2 | 0.12870 | 0.12815 | 0.13369 | 0.14014 | 0.14332 |
| DSLR2 | 0.03040 | 0.02586 | 0.03450 | 0.03421 | 0.03314 |
| DSL | 0.12886 | 0.12850 | 0.13379 | 0.13949 | 0.14269 |
| PM | 0.12900 | 0.12916 | 0.13414 | 0.13869 | 0.14199 |
| RML | 0.12892 | 0.12945 | 0.13488 | 0.14373 | 0.14718 |
| SJ | 0.22491 | 0.26003 | 0.27844 | 0.28173 | 0.28787 |

Note: DSLD2 represents the two-step estimation starting with the DSL estimate and the $D^{2}$ in the second step that this paper developed; DSLR2 is the two-step estimation starting with the DSL estimate and the $R^{2}$ in the second step; DSL denotes the standard random-effects model.
