# Supplementary material for: A novel estimator of between-study variance in random-effects models
Source: BMC Genomics. 2020 Feb 11;21:149. doi: 10.1186/s12864-020-6500-9 (PMC7014785; doi:10.1186/s12864-020-6500-9)
Supplement: Supplementary file 5 — Additional file 5 The r code. Additional file 5 is the code of the simulation setting for gene expression levels, the code of the simulation data using Monte Carlo method and the R code of DSLD2 method. [file 12864_2020_6500_MOESM5_ESM.docx]

Additional file 5

Nan Wang^1†^, Jun Zhang^2†^, Li Xu^3†^, Jing Qi^1^, Beibei Liu^1^, Yiyang Tang^4^, Yinan Jiang^5^, Liang Cheng^6^, Qinghua Jiang^7^, Xunbo Yin^1^ and Shuilin Jin^1*^

1. Department of Mathematics, Harbin Institute of Technology, Harbin, Heilongjiang, China

2. College of Computer Science and Technology, Harbin Engineering University, Harbin, China

3. School of Life Science and Technology, Harbin Institute of Technology, Harbin, China

† Equally contributed to the work

* To whom all correspondence should be addressed

*Corresponding author:

Shuilin Jin

School of Mathematics, Harbin Institute of Technology, Harbin, Heilongjiang, China

E-mail:jinsl@hit.edu.cn

The code for the simulation setting of gene expression levels:

myfunction<-function(nsam){ #nsam is the number of sample size

pusai<-read.table("pusai.txt",header=FALSE)

class(pusai)

pusai<-data.matrix(pusai)

class(pusai)

library(MCMCpack)

library(stats)

library(tmvtnorm)

Sigma<-runif(5,min=0.8,max=1.2)

Sigma<-as.matrix(Sigma)

for (k in 1:5){

Sigma_e=Sigma[k]

z<-matrix(c(rep(0,20*nsam)),20,nsam)

w<-matrix(c(rep(0,1200)),1200,1)

for (c in 1:40){

sig_1<-riwish(60,pusai)

write.table(sig_1,file=paste("sig_1_",c,"_",k,".txt"), sep="\t")

standardy=matrix(c(rep(0,400)),20,20)

for (i in 1:20){

standardy[i,i]=1.0/sqrt(sig_1[i,i])

}

write.table(standardy,file="standardy.txt", sep="\t")

sig=standardy%*%sig_1%*%standardy

write.table(sig,file=paste("sig_",c,"_",k,".txt"), sep="\t")

y=matrix(c(rep(0,20)),20,1)

for (n in 1:nsam){

x<-rmvnorm(n=1,mean=c(rep(0,20)),sigma=sig,method="chol")

x<-t(x)

y<-cbind(y,x)

}

y<-y[,-1]

y<-as.matrix(y)

z<-rbind(z,y)

}

z<-z[-(1:20),]

z<-as.matrix(z)

write.table(z,file=paste("simple_800",k,".txt"),sep="\t")

for (j in 1:nsam){

number_1200<-rnorm(1200,mean = 0,sd =Sigma_e )

number_1200<-as.matrix(number_1200)

w<-cbind(w,number_1200)

}

w<-w[,-1]

result<-rbind(z,w)

write.table(result,file=paste("simple_2000",k,".txt"),sep="\t")

}

A<-matrix(c(rep(0,5)),1,5)

for (j in 1:5){

for (i in 1:200){

derta_1<-rep(0,5)

a<-sample(1:5,j)

derta_1[a]<-1

A<-rbind(A,derta_1)

}

}

A<-A[-1,]

write.table(A,file= "3.derta.txt", row.names=c(1:1000), sep="\t")

a<-read.table("simple_2000 1 .txt",header=TRUE,row.names=1,sep="\t")

b<-read.table("simple_2000 2 .txt",header=TRUE,row.names=1,sep="\t")

c<-read.table("simple_2000 3 .txt",header=TRUE,row.names=1,sep="\t")

d<-read.table("simple_2000 4 .txt",header=TRUE,row.names=1,sep="\t")

e<-read.table("simple_2000 5 .txt",header=TRUE,row.names=1,sep="\t")

A<-read.table("3.derta.txt",header=TRUE,sep="\t")

a<-data.matrix(a)

b<-data.matrix(b)

c<-data.matrix(c)

d<-data.matrix(d)

e<-data.matrix(e)

A<-data.matrix(A)

for(i in 1:5){

if (i==1) matrix<-a

if (i==2) matrix<-b

if (i==3) matrix<-c

if (i==4) matrix<-d

if (i==5) matrix<-e

u =runif(1000,min=0.5,max=3)

u<-as.vector(u)

col<-A[,i]

col<-as.vector(col)

increase<-u*col

for (j in 1:(nsam/2)){

matrix[1:1000,(nsam/2)+j]=matrix[1:1000,(nsam/2)+j]+increase

}

write.table(matrix,file=paste("sample_2000_result_",i,".txt"),sep="\t")

}

}

The code for the simulation setting using Monte Carlo method:

#k is the number of studies, mu_sita is the overall mean value, sigma_sita is the between study variance, n_i_bar is the mean value of normal distribution.

simulation <- function(k, mu_sita, sigma_sita, n_i_bar){

n <- ceiling(rnorm(k, n_i_bar, n_i_bar/3))

for (i in 1:k){

n_i <- n[i]

x_i<-matrix(rep(NA, g*n_i*2), g, n_i*2)

for (j in 1:g){

x_i_ctrl <- rnorm(n_i,0,10)

mu_i_case <- rnorm(1 ,mu_sita, sigma_sita)

x_i_case <-rnorm(n_i, mu_i_case,10)

x_i[j,1:n_i] <- x_i_ctrl

x_i[j,(n_i+1):(2*n_i)] <- x_i_case

}

colname <- c(paste("ctrl", 1:n_i), paste("case", 1:n_i))

rowname <- paste("g", 1:g, sep="")

write.table(x_i, file=paste("study_", as.character(i), ".txt",sep=""), sep="\t", col.names = colname, row.names = rowname)

}

}

The R code of DSLD2 method:

#x is a list with components.

#ES: The observed effect sizes.

#Var: The observed variances corresponding to ES

. #perm.ES: The effect sizes calculated from permutations, perm.ES is NULL if the argument nperm is set as NULL.

#perm.Var: The corresponding variances calculated from permutations. perm.Var is NULL if the argument nperm is set as NULL.

library(impute)

library(Biobase)

library(combinat)

Meta<-function(x, meta.method = "DSLD2"){

meta.method<-match.arg(meta.method)

K<-ncol(x$ES)

res<-get.REM(x$ES,x$Var,pe=x$perm.ES,pv=x$perm.Var)

tempFDR<-matrix(res$FDR,ncol=1)

rownames(tempFDR)<-rownames(x$ES)

colnames(tempFDR)<-meta.method

meta.res<-list(mu.hat=res$mu.hat,mu.var=res$mu.var,Qval=res$Qval,Qpval=res$Qpval,tau2=res$tau2,zval=res$zval,pval=res$pval,FDR=tempFDR, I2 = res$I2, H2=res$H2)

attr(meta.res,"nstudy")<-K

attr(meta.res,"meta.method")<-meta.method

class(meta.res)<-"MetaDE.ES"

return(meta.res)

}

get.Q<-function(em,vm){

wt <- 1/vm

temp1 <- wt * em

mu.hat <- rowSums(temp1)/rowSums(wt)

Q <- rowSums(wt * (em - mu.hat)^2)

return(Q)

}

get.tau2.DSL<-function(Q,vm,k){

wt<-1/vm

s1 <- rowSums(wt)

s2 <- rowSums(wt^2)

temp<- (Q - (k - 1))/(s1 - (s2/s1))

tau2<-pmax(temp,0)

return(tau2)

}

get.SMM <-function(em,vm){

k<-ncol(em)

Q.val<-get.Q(em,vm)

tau2<-get.tau2.DSL(Q.val,vm,k)

temp.wt<-1/(vm+tau2)

mu.hat<-rowSums(temp.wt*em)/rowSums(temp.wt)

SMM <- rowSums(temp.wt * (em - mu.hat)^2)

return(SMM)

}

get.tau2<-function(Q,SMM,vm){

wt<-1/vm

s1 <- rowSums(wt)

s2 <- rowSums(wt^2)

temp<- (Q - SMM)/(s1 - (s2/s1))

tau2<-pmax(temp,0)

return(tau2)

}

get.REM2<-function(em,vm){

k<-ncol(em)

Q.val<-get.Q(em,vm)

SMM<-get.SMM(em,vm)

tau2<-get.tau2(Q.val,SMM, vm)

temp.wt<-1/(vm+tau2)

mu.hat<-rowSums(temp.wt*em)/rowSums(temp.wt)

mu.var<-1/rowSums(temp.wt)

Qpval <- pchisq(Q.val, df = k - 1, lower.tail = FALSE)

z.score<-mu.hat/sqrt(mu.var)

z.p<-2*(1-pnorm(abs(z.score)))

qval<-p.adjust(z.p,method="BH")

res<-list(mu.hat=mu.hat,mu.var=mu.var,Qval=Q.val,Qpval=Qpval,tau2=tau2,zval=z.score,pval=z.p,FDR=qval)

return(res)

}

get.REM<-function(em,vm,pe=NULL,pv=NULL){

k<-ncol(em)

Q.val<-get.Q(em,vm)

SMM<-get.SMM(em,vm)

tau2<-get.tau2(Q.val,SMM,vm)

temp.wt<-1/(vm+tau2)

mu.hat<-rowSums(temp.wt*em)/rowSums(temp.wt)

mu.var<-1/rowSums(temp.wt)

Qpval <- pchisq(Q.val, df = k - 1, lower.tail = FALSE)

z.score<-get.REM2(em,vm)$zval

if(!is.null(pe)&!is.null(pv)){

rnum<-which(apply(em,1,function(x) !any(is.na(x))))

Z0<-matrix(get.REM2(pe,pv)$zval,nrow(em),nrow(pe)/nrow(em))

z.p<-rep(NA,nrow(em))

z.p[rnum]<-perm.p(z.score[rnum],Z0[rnum,],"abs")

}else{

z.p<-2*(1-pnorm(abs(z.score)))

}

qval<-p.adjust(z.p,method="BH")

I2<-get.I2(em,vm)

H2<-get.H2(em,vm)

res<-list(mu.hat=mu.hat,mu.var=mu.var,Qval=Q.val,Qpval=Qpval,tau2=tau2,zval=z.score,pval=z.p,FDR=qval, I2=I2, H2=H2)

return(res)

}

perm.p<-function(stat,perm,tail) {

G<-length(stat)

B<-length(perm)/G

if(tail=="low"){

r = rank(c(stat, as.vector(perm)),ties.method="max")[1:G]

r2 = rank(c(stat),ties.method="max")

r3 = r - r2

p = r3/(B*G)

}

if(tail=="high"){

r = rank(c(stat, as.vector(perm)),ties.method="min")[1:G]

r2 = rank(stat,ties.method="max")

r3 = r - r2

p = 1-r3/(B*G)

}

if(tail=="abs"){

r = rank(c(abs(stat), abs(as.vector(perm))),ties.method="min")[1:G]

r2 = rank(c(abs(stat)),ties.method="max")

r3 = r - r2

p = 1-r3/(B*G)

}

p[p==0]<-1e-20

p[p==1]<-1-1e-10

return(p)

}

get.I2 <- function(em,vm){

k<-ncol(em)

Q.val<-get.Q(em,vm)

SMM<-get.SMM(em,vm)

tau2<-get.tau2(Q.val,SMM,vm)

temp.wt<-1/(vm+tau2)

#vi.avg <- 1/mean(wi)

vi.avg <- (k-1) / (apply(temp.wt, 1, sum) - apply(temp.wt^2, 1, sum)/apply(temp.wt, 1, sum))

I2 <- tau2 / (vi.avg + tau2)

return(I2)

}

get.H2 <- function(em,vm){

k<-ncol(em)

Q.val<-get.Q(em,vm)

SMM<-get.SMM(em,vm)

tau2<-get.tau2(Q.val,SMM,vm)

temp.wt<-1/(vm+tau2)

vi.avg <- (k-1) / (apply(temp.wt, 1, sum) - apply(temp.wt^2, 1, sum)/apply(temp.wt, 1, sum))

H2 <- tau2 / vi.avg + 1

return(H2)

}
